# Between the Cape Fold Mountains and the deep blue sea: Comparative phylogeography of selected codistributed ectotherms reveals asynchronous cladogenesis

**DOI:** 10.1111/eva.13493

**Published:** 2022-10-27

**Authors:** Angus Macgregor Myburgh, Savel Regan Daniels

**Affiliations:** ^1^ Department of Botany & Zoology University of Stellenbosch Stellenbosch South Africa

**Keywords:** climatic fluctuations, divergence time estimation, Greater Cape Floristic Region, phylogeographic concordance, phylogeography, South Africa

## Abstract

We compare the phylogeographic structure of 13 codistributed ectotherms including four reptiles (a snake, a legless skink and two tortoise species) and nine invertebrates (six freshwater crabs and three velvet worm species) to test the presence of congruent evolutionary histories. Phylogenies were estimated and dated using maximum likelihood and Bayesian methods with combined mitochondrial and nuclear DNA sequence datasets. All taxa demonstrated a marked east/west phylogeographic division, separated by the Cape Fold Mountain range. Phylogeographic concordance factors were calculated to assess the degree of evolutionary congruence among the study species and generally supported a shared pattern of diversification along the east/west longitudinal axis. Testing simultaneous divergence between the eastern and western phylogeographic regions indicated pseudocongruent evolutionary histories among the study taxa, with at least three separate divergence events throughout the Mio/Plio/Pleistocene epochs. Climatic refugia were identified for each species using climatic niche modelling, demonstrating taxon‐specific responses to climatic fluctuations. Climate and the Cape Fold Mountain barrier explained the highest proportion of genetic diversity in all taxa, while climate was the most significant individual abiotic variable. This study highlights the complex interactions between the evolutionary history of fauna, the Cape Fold Mountains and past climatic oscillations during the Mio/Plio/Pleistocene. The congruent east/west phylogeographic division observed in all taxa lends support to the conclusion that the longitudinal climatic gradient within the Greater Cape Floristic Region, mediated in part by the barrier to dispersal posed by the Cape Fold Mountains, plays a major role in lineage diversification and population differentiation.

## 1 INTRODUCTION

The Greater Cape Floristic Region (GCFR), a global biodiversity hotspot, has undergone considerable geological and environmental change, which dramatically impacted the habitat types and distribution of faunal and floral species (Tolley et al., 2014). Ancient climatic fluctuations during the early and late Miocene (23‐5 Ma) resulted in at least three major cycles of glacial advance and retreat (Hannah, 2006), causing marine transgressions of up to 150 m above the present level (Rogers, 1987). These cycles, paired with continental uplift (Partridge & Maude, 2000), produced a mosaic of different soil types in the present‐day Cape, increasing habitat diversity (Cowling et al., 2009). The Miocene was characterized by relatively warmer and wetter climatic regimes and saw increasing deterioration (cooling and drying) until the beginning of the Pliocene (5–2.5 Ma), a process which played a major role in the diversification within GCFR floral lineages (Bakker et al., 2000; Linder, 2003; Verboom et al., 2003). This aridification was largely driven by the development of the proto‐Benguela upwelling system along the Cape west coast, which brought cooler waters to the Cape, resulting in decreased evaporation and precipitation and which reached a climax during the transition into the Pliocene (Marlow et al., 2000). The rapid cooling coincided with renewed glaciation in Antarctica, which lead to decreased sea levels (Tyson & Partridge, 2000) and contributed towards the progressive aridification in the Cape, reducing ocean surface area and decreasing the total solar radiation absorbed, thereby reducing precipitation (Theron, 1983). During the Pliocene, southern Africa experienced a second tectonic uplift event, which lasted from 5 to 3 Mya (Partridge & Maude, 1987). While the east of the country saw uplift as great as 900 m, the GCFR experienced uplift of 200–300 m in the east, and 150 m in the west (Partridge & Maude, 2000). This tectonic activity and increased glaciation caused the coastline to recede even further, leading to the exposure of clay‐rich substrata and stimulating the diversification of lowland lineages (Cowling et al., 2009). While the middle Pliocene saw a temporary return to wetter, warmer conditions (Fedorov et al., 2006), the Pleistocene (2.5 Mya–20 ka) experienced several icehouse‐hothouse cycles (Tyson & Partridge, 2000), with an overall progressive aridification (Fedorov et al., 2006). These alternating glacial and interglacial periods caused sea levels to fluctuate dramatically, periodically exposing large tracts of coastal land (Dingle & Rogers, 1972). Much of the diversity within the GCFR has been the result of relatively recent divergences during these periods, resulting in shallow genetic lineages (Tolley et al., 2014), and it is likely that the climatic oscillations during this time served as a major driver of cladogenesis.

**FIGURE 1:**
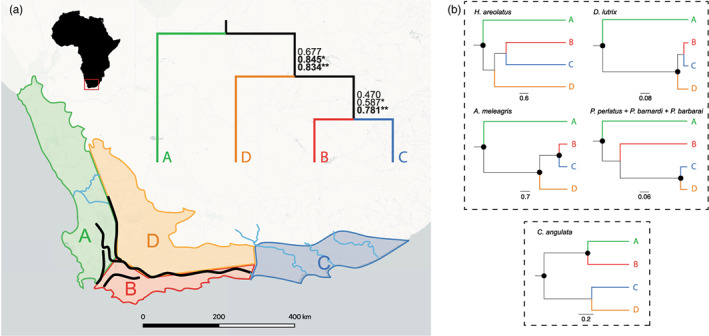
Greater Cape Floristic Region (GCFR) of South Africa (a), demonstrating the known biogeographical barriers and divided into broad subregions based on the phylogeographic results of the present study. The Cape Fold Mountain (CFM) range is indicated with dark lines and separates the western GCFR (A) from the southeastern GCFR (B) and the central GCFR (D). The eastern GCFR (C) is indicated in blue. Region‐scale phylogenetic concordance factors (PCFs) are shown, with values indicating high levels of congruence (>0.71) highlighted in bold. Uppermost values include all taxa indicated in (b), while values with an asterisk (*) exclude *C. angulata*, and values with two asterisks (**) exclude both *C. angulata* as well as the combined *P. perlatus*, *P. barnardi* and *P. barbarai*. Taxon‐specific phylogeographic relationships between the GCFR subregions are shown on the right.

These historical climatic changes have resulted in several biotic and abiotic differences between the eastern and western regions of the GCFR, with the Cape Fold Mountains (CFM) acting as a broad interface between these two regions (Figure 1). Aridification during the Miocene led to strengthened rainfall seasonality in the west (Hoffmann et al., 2015; Neumann & Bamford, 2015), while the east experienced a major overall reduction in rainfall during glacial times (Cowling et al., 1999). The western GCFR is thought to have remained relatively stable since the end of the Miocene (Linder, 2003); as stable environments are able to act as refugia, extinction rates are expected to be lower, thus increasing genetic diversity and species richness (Tolley et al., 2014). Indeed, it has been suggested that this stability directly contributed towards the unusually high levels of diversity and endemism within the GCFR (Cowling et al., 2009), with several species demonstrating higher levels of phylogenetic structure and genetic diversity in the western than in the eastern region of the GCFR (Barlow et al., 2013; McDonald & Daniels, 2012; Tolley et al., 2006). In comparison with the gradual increase in species richness in the west, many clades in the east are either extremely recent (as a result of colonization events during the late Pleistocene; Smit et al., 2007), or else extremely ancient, potentially due to a number of deep valleys in the east which may have acted as ancient refugia (Forest et al., 2007). The steep climatic gradient between the east and west is reflected in changes in vegetation, which has been posited as a major driver of local adaptations and therefore diversification (Hofmeyr et al., 2020).

Indeed, previous single‐species phylogeographic studies of taxa distributed throughout the GCFR corroborated the east/west phylogenetic division, with the CFM representing a major barrier to gene flow in both vertebrates and invertebrates (Daniels et al., 2007; Smit et al., 2007; Swart et al., 2009; Tolley et al., 2006; Willows‐Munro & Matthee, 2011) (Figure 1). Divergence time estimations date the cladogenesis in the aforementioned species to the Mio/Plio/Pleistocene (Daniels et al., 2006; Engelbrecht et al., 2013; Hofmeyr et al., 2020; Phiri & Daniels, 2014). The ancient cladogenic events resulting in the east and western clade divergence were attributed to niche exploitation during historical periods of marine regressions during the Mio/Plio/Pleistocene due to the appearance of a coastal plain facilitating migration around the CFM, followed by the differentiation of populations after marine transgressions removed these dispersal corridors. Ectotherms are particularly vulnerable to reductions in minimum annual temperature, which potentially limit their dispersal and survival capabilities, and so the climatic changes during this period would have served to isolate populations from one another, resulting in cladogenesis (Damgaard et al., 2008; Daniels et al., 2007, 2009; Engelbrecht et al., 2013; Hofmeyr et al., 2020; Kulenkampff et al., 2019; McDonald & Daniels, 2012; Myburgh & Daniels, 2015; Phiri & Daniels, 2014; Price et al., 2007; Wood & Daniels, 2016).

Broadly congruent estimates of divergence times among codistributed species across the GCFR suggest that shared factors are likely causal to the observed pattern of cladogenesis. Similarities in phylogeographic patterning were historically determined by visual comparison of tree topologies; however, these similarities can arise from congruent spatial and temporal patterns, independent responses to climate or multiple responses all producing identical divergence patterns. Recent, novel approaches, such as the use of phylogeographic concordance factors (PCFs), enable researchers to statistically test the degree of congruence between phylogeographic patterns (Satler & Carstens, 2016). Similarly, software packages have been designed to specifically assess synchronicity between cladogenesis events among codistributed taxa (Oaks, 2019). During the present study, we investigated and compared the evolutionary histories of 13 species (in seven taxon groups) codistributed throughout the GCFR. These included four reptiles (the slug‐eating snake *Duberria lutrix*, the Cape legless skink *Acontias meleagris*, the two tortoises *Homopus areolatus* and *Chersina angulata*), six freshwater crabs (*Potamonautes perlatus*, *Potamonautes barnardi*, *Potamonautes barbarai*, *Potamonautes parvicorpus*, *Potamonautes tuerkayi* and *Potamonautes brincki*), as well as three velvet worm species (*Peripatopsis capensis*, *Peripatopsis lawrencei* and *Peripatopsis overbergiensis*). Our aims in comparing the phylogeographic structure of the 13 aforementioned species are fourfold: (1) to assess and compare the levels of genetic diversity of the study taxa within the eastern and western GCFR; (2) to statistically test the levels of phylogeographic congruence between the 13 taxa using PCFs; (3) to statistically compare divergence time estimates between western and eastern lineages within these taxa; and (4) to assess the CFM and climatic gradients as drivers of cladogenesis and differentiation. We hypothesize that (1) these species will display high levels of phylogeographic congruence, indicating that the same topographical changes and climatic changes throughout the Plio‐ and Pleistocene underlie their evolutionary histories; (2) divergence time estimates between these species will indicate pseudocongruent evolutionary histories, likely due to partially shared responses to climatic fluctuations; (3) the CFM acts as a significant barrier to gene flow and thus serves as a driver of cladogenesis, while environmental gradients between the eastern and western GCFR contributed to the observed phylogenetic patterns of each species.

## 2 MATERIALS AND METHODS

### 2.1 Study taxa and sequence data

Both nuclear (nuDNA) and mitochondrial (mtDNA) DNA sequence data for the 13 codistributed study species generated in previous studies were downloaded from GenBank, along with the most appropriate outgroups for each species (Daniels et al., 2005, 2006, 2007, 2009; Engelbrecht et al., 2013; Hofmeyr et al., 2020; Kulenkampff et al., 2019; Wood & Daniels, 2016). GenBank accession numbers for each of the samples for each species per loci are listed in Appendix S2: Table S2, while the loci and number of samples used per taxon are summarized in Appendix S3: Table S3.1. Four mtDNA markers were used in the present study, namely the nicotinamide adenine dinucleotide dehydrogenase 4 gene (ND4; *H. areolatus*, *C. angulata and D. lutrix*), the cytochrome b gene (cytb; *C. angulata* and *D. lutrix*), the 16S rRNA gene (16S rRNA; *P. perlatus*, *P. barnardi* and *P. barbarai*) and the cytochrome *c* oxidase subunit I locus (COI; *A. meleagris*, *P. brincki*, *P. parvicorpus*, *P. tuerkayi*, *P. perlatus*, *P. barnardi*, *P. barbarai*, *Per. capensis*, *Per. lawrencei* and *Per. overbergiensis*). Nuclear DNA markers included the prolactin receptor gene (PRLR; *H. areolatus*), the exophilin‐5 gene (EXPH5; *A. meleagris*) and spectrin beta, nonerythrocytic locus one (SPTBN1; *D. lutrix*). Although the 18S nDNA marker was available for *Peripatopsis*, initial analyses indicated high levels of substitution saturation and thus it was not included in downstream analyses. In order to compare eastern and western populations within the study taxa, the three *Peripatopsis* sister species were combined and treated as a single monophyletic taxon (McDonald & Daniels, 2012). Similarly, the six *Potamonautes* species were combined into two separate monophyletic taxon groups, with one group comprised of *P. perlatus* + *P. barbarai* + *P. barnardi* (Phiri & Daniels, 2014) and the other consisting of *P. brincki* + *P. parvicorpus* + *P. tuerkayi* (Wood & Daniels, 2016).

### 2.2 Mitochondrial phylogenies and divergence time estimation

Both maximum likelihood (ML) and Bayesian inference (BI) methods were used to estimate the gene and species trees using the respective DNA data for each taxon. The saturation for each gene locus was assessed using DAMBE 7.0.58 (Xia, 2017), and loci showing high levels of saturation were excluded from downstream analyses (specifically the 18S nuDNA locus for *Per. capensis*, *Per. lawrencei* and *Per. overbergiensis*, as well as the COI mtDNA locus for *C. angulata*). The online tool W‐IQ‐TREE (Trifinopoulos et al., 2016) was used to estimate the ML phylogenies, as well as to determine the best‐fit substitution model for each gene under the Bayesian information criterion (BIC) using the ‘auto’ option (Appendix S3: Table S3.3). The ultrafast bootstrap approximation method was used with 10,000 alignments in order to assess nodal support (Hoang et al., 2018), with bootstrap values ≥75% being considered as well‐supported. BI phylogenies were estimated using BEAST 2.6.3 (Bouckaert et al., 2019) and were dated using mtDNA mutation rates due to the lack of fossil calibration points for the study taxa, with nodes with posterior probability (pP) ≥0.95 considered to be highly supported. The constancy of mutation rates among the clades in each topology was assessed using Tajima's relative rate test implemented in Mega X 10.0.5 (Kumar et al., 2018). Each BI phylogeny was estimated using the best‐fit substitution model determined by IQ‐TREE (including the gamma shape and proportion invariant values), with a coalescent tree prior assuming constant population sizes and with a relaxed lognormal molecular clock. Each analysis was run for 20 × 10^6^ generations and sampled every 1000 generations. Due to the use of both nuclear and mitochondrial markers, as well as the range of taxa studied, a number of mutation rates were used to date each phylogeny (see Appendix S3: Table S3.2 and Appendix S1 for details).

Phylogenies were estimated using multiple mtDNA loci where possible, and therefore, the molecular clocks were unlinked during the analyses to allow for independent mutation rates for each gene. Loci in which IQ‐TREE retrieved substitution models unsupported by BEAST 2.6.3 were run using the most complex GTR model. Chain convergence was assessed using Tracer 1.6 (Rambaut et al., 2018) to ensure that effective sample sizes (ESS) remained over 200. Any analyses displaying ESS values lower than 200 were re‐run for an additional 20 × 10^6^ generations, after which the log files were combined using LogCombiner and reassessed. TreeAnnotator was used to discard 20% of each run as burn‐in, as well as to determine the maximum clade credibility tree. Tree topologies were edited using FigTree 1.4.3 (Rambaut, 2008).

### 2.3 Population divergence time

Denim 3.1 (Jones, 2018) was used to estimate both population divergence times and phylogenetic relationships among the populations based on the combined mtDNA and nDNA datasets for each species. Populations were separated based on the clades identified by the mtDNA phylogenies, which were either genetically divergent intraspecific populations or else represented separate described species based on previous phylogenetic work (McDonald & Daniels, 2012). The mtDNA priors used were the same as those specified in the BEAST analyses. The HKY substitution model was used for the nDNA loci in order to prevent over‐parameterization, and the mutation rates were estimated with a starting mean of one, using a strict molecular clock with a lognormal distribution. The simple model for migration was used in the analyses, with an exponential prior and a mean of 0.001, and was run for 500 × 10^6^ generations (sampling every 10,000 generations). Chain convergence (which was considered when the ESS values exceeded 200) was assessed using Tracer, and Treeannotator was used to remove the first 20% of the sampled trees as burn‐in, as well as to identify the maximum clade credible tree.

### 2.4 Population genetic analyses

TCS 1.21 (Clement et al., 2000) was used to construct haplotype networks, to investigate the genetic relationships among the sample localities in each of the study taxa, using statistical parsimony with 95%. Popart (Leigh & Bryant, 2015) and Adobe Illustrator (Adobe Inc, 2019) were used to create the final haplotype network figures. A hierarchical AMOVA was performed using Arlequin 3.1 (Excoffier et al., 2005) for each mtDNA locus for each species across the eastern and western regions, as well as each sampling locality in order to investigate the distribution of genetic variation. Furthermore, an AMOVA was conducted for each population separately in order to deduce the genetic variation within each region. DnaSP 6.12 (Rozas et al., 2017) was used to calculate the standard descriptive statistics and demographic changes for each population and species, including the number of haplotypes (H), haplotype diversity (h), number of segregating sites (S), Tajima's D (Tajima, 1989) and Fu's Fs (Fu, 1997).

### 2.5 Phylogeographic concordance factors

In order to investigate the degree of phylogeographic congruence among the seven study taxa, phylogeographic concordance factors (PCFs) were calculated. PCFs statistically determine the prevailing phylogeographic pattern and the species which share this pattern (Satler & Carstens, 2016). As PCF analyses require sequences from every locality, we performed two approaches. In the primary analysis, five broad geographical subregions within the GCFR were delineated based on a combination of natural geographical barriers (such as the Cape Fold Belt, and the Olifants and Gamtoos rivers), floral biome interfaces (specifically between the Fynbos and Succulent Karoo biomes) as well as the distribution of the taxon clades resolved from the BEAST analyses (Figure 1a,b). This PCF analysis included only the species *H. areolatus*, *D. lutrix*, *A. meleagris*, *C. angulata*, as well as the combined *P. perlatus* + *P. barnardi* + *P. barbarai*. In the second approach, we performed a number of analyses with reduced species combinations, each containing samples from localities shared by each taxon (within a maximum distance of 10 km; Figure S1A–F). The mtDNA datasets for each taxon were used in *BEAST (Heled & Drummond, 2010) to construct the posterior trees. The species tree approach was used to assign samples to localities (or locality clusters), using the HKY substitution model for the reduced mtDNA datasets with a coalescent tree prior, a strict molecular clock, and assuming a constant population size. The *BEAST analyses were run for 20 × 10^6^ generations, sampling every 1000 generations, after which Tracer was used to assess the chain convergence (ESS > 200). The PCF values were calculated from the posterior species tree distributions using a python script by Satler & Carstens (2016; PCFs.py; https://github.com/jordansatler/PhylogeographicConcordanceFactors). Burn‐in was set at 10%, and each species tree distribution was summarized using MBSUM (Larget et al., 2010). BUCKy (Ané et al., 2007) was used to process the tree summary files in order to construct a concordance tree with PCF values, as well as to determine the average PCF values among the taxa. In accordance with the guidelines set out by Satler and Carstens (2016), phylogeographic history congruity is reached when PCF ≥ 0.71.

### 2.6 Tests for simultaneous divergence

We tested for simultaneous divergence between the western and eastern phylogeographic regions, using the single mtDNA loci, combined mtDNA and total evidence datasets where available. Divergence times were estimated using *BEAST, using the same substitution models and relative mutation rates as those used in the initial BEAST analyses, and populations were defined based on the previously resolved eastern and western monophyletic clades. While *BEAST takes into account uncertainty in the mutation rate, this often results in broad confidence intervals which would overestimate the overlap in divergence times between species. Consequently, we also specifically tested for simultaneous divergence using a single mtDNA locus per taxon, as well as combined mtDNA and a total evidence approach with ecoevolity (Oaks, 2019), which specifically assesses synchronous divergence. For more detailed information, see Appendix S1.2.

### 2.7 Climatic niche modelling

In order to determine whether the major phylogenetic patterns uncovered by this study aligned with congruent climatic refugia among the study taxa, a niche modelling approach was used. Maxent 3.4.1 (Phillips et al., 2006, 2017) was used to identify persistent Pleistocene refugia based on several contemporary and paleoclimatic scenarios, namely the current (1950–2000), mid‐Holocene (6 ka), last glacial maximum (22 ka) and last interglacial (120–140 ka) time periods. All 19 bioclimatic variables were downloaded from the WorldClim database (Hijmans et al., 2005) and cropped to the geographical extent of South Africa using ArcGIS (ESRI, 2012). Correlated variables (with a Pearson's correlation coefficient >0.7) for each species were assessed using the r package CorrPlot (Wei & Simko, 2021) in RStudio 2021.09.1 (RStudio Team, 2020) and were removed, preferentially retaining variables known to influence the distribution of each taxon (Bucklin et al., 2015; Cunningham et al., 2016; El‐Gabbas et al., 2016; Hijmans et al., 2005; Lawson, 2010; Leaché et al., 2019; Parvizi et al., 2018, 2019) and the remaining limiting climatic variables were used to generate the models (Appendix S3: Table S3.8). A custom R script was used to specify a fixed distance buffer of 50 km based on the occurrence records for model building in order to correct the models for overprediction, as well as to produce minimum convex polygons of the distribution for each taxon. ENMeval 2.0 (Kass et al., 2021) was used to determine the optimum feature class and regularization multiplier for model construction, after which Maxent 3.4.1 (Phillips et al., 2004, 2006) was used to generate the SDMs. The models were converted from continuous probability surfaces to binary models representing either suitable or unsuitable climatic conditions based on a 10% training presence cloglog threshold using QGIS 3.24.2 (QGIS.org, 2022). Possible climatic refugia were inferred for each taxon by overlaying the binary models from the various time periods, thereby identifying areas which remained within the habitat requirements of the taxa over time. Finally, the species‐specific refugia were intersected in order to determine the shared regions of climatic stability. The final maps were generated using QGIS 3.24.2.

### 2.8 Climatic niche comparison

Variations in climate among populations for each of the study species between the two geographic regions were explored as potential contributors towards genetic diversity. All 19 bioclimatic variables were used to statistically test the differences in climate between the genetically divergent populations of each taxon. Climatic data were extracted for each locality, with duplicate or nearby localities which shared the same climatic variables removed from the analyses. The variables were all standardized for the multivariate analysis, and the relative climates of each population were visualized using a principal component analysis (PCA). A permutational multivariate analysis of variance (PERMANOVA) with 9999 permutations was used to statistically test the differences in climate among the populations, and a Kruskal–Wallis test was conducted on each variable in order to identify the significantly different variables which contributed to the overall variation. Finally, PAST 4.02 (Hammer et al., 2001) was used to conduct a Spearman's rank correlation test in order to determine whether the variation in each climatic variable was correlated with longitude.

### 2.9 Abiotic associations with genetic structure

Correlations between genetic variation and abiotic variables (such as the geographic distance between localities, the current climate as well as the location of the CFM) were assessed using redundancy analyses (RDA). A normalized genetic distance matrix was generated for each species based on the most‐sampled mtDNA locus per taxon using an R script (Myers et al., 2017), after which a principal coordinate analysis (PCoA) was performed. The response variables for the RDAs consisted of the genetic distance PCoA matrices, while the explanatory variables were constituted by the current climate, geographic distances between the sampling localities, as well as populations west or east of the CFM. These analyses were repeated with different combinations of the explanatory variables (resulting in 49 analyses), controlling for the variables excluded from each analysis.

## 3 RESULTS

### 3.1 Phylogenetic relationships and divergence time

**FIGURE 2:**
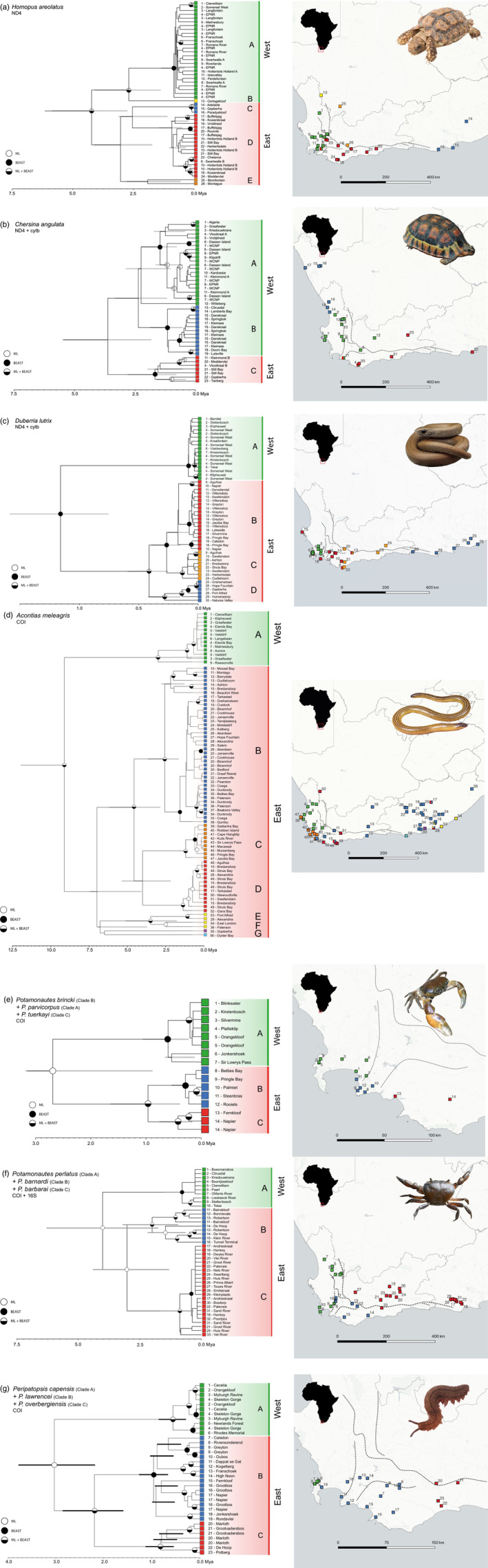
Bayesian phylograms (left) and corresponding sampling locality maps (right) for the sample taxa, namely *H. areolatus* (a), *C. angulata* (b), *D. lutrix* (c), *A. meleagris* (d), *P. brincki* + *P. parvicorpus* + *P. tuerkayi* (e), *P. perlatus* + *P. barbarai* + *P. barnardi* (f), and *per. Capensis* + *per. Lawrencei* + *per. Overbergiensis* (g). Node symbols indicate high statistical support, with black nodes indicating pP >0.95, and white nodes indicating ML bootstrap values >75%. Divergence time estimations are shown as million years ago (Mya), and 95% highest posterior density bars are indicated in grey. Dotted lines indicate the position of the CFM range.

The BEAST and ML topologies were largely congruent and retrieved two statistically well‐supported western and southeastern clades where the Hottentots Holland Mountain was retrieved as the main phylogeographic barrier in all the codistibuted species (Figure 2a–g). In addition, the southeastern clade generally consisted of several additional, monophyletic clades which clearly grouped into separate geographical areas extending from the Western Cape Province into the Eastern Cape Province, while the western clade consisted of a single monophyletic clade per species (Figure S2). Exceptions to the latter pattern included two reptiles, the angulated tortoise, *C. angulata*, which consisted of two western clades (a southwestern and northwestern clade), and the Cape legless skink, *A. meleagris*, which featured a clade exclusively located in the western region that was sister to the southern and eastern clade.

Divergence time estimation in BEAST based on the mtDNA datasets revealed that, within the species with populations on both sides of the CFM, *A. meleagris* harboured the earliest divergence between the southwestern and eastern clades at 8.31 Mya (95% HPD: 5.12–12.52) during the Miocene, followed by *H. areolatus* 4.22 Mya (95% HPD: 2.74–5.86), *C. angulata* Mya 3.62 (95% HPD: 1.82–5.49), and finally most recently *D. lutrix* 1.16 Mya (95% HPD: 0.75–1.59) all of which occurred during the Plio/Pleistocene. *Potamonautes perlatus* diverged from the southeastern *P. barnardi* and *P. barbarai* 4.01 Mya (95% HPD: 1.76–6.29), while the latter two species diverged 3.02 Mya (95% HPD 1.31–4.41). The western *P. parvicorpus* diverged from the southeastern *P. brincki* and *P. tuerkayi* 2.69 Mya (95% HPD: 0.88–3.18), after which the southeastern species diverged 0.96 Mya (95% HPD 0.38–1.64). Finally, the western *Per. capensis* diverged from *Per. lawrencei* and *Per. overbergiensis* 3.04 Mya (95% HPD: 2.19–3.78), while the latter species diverged 2.20 Mya (95% HPD 1.60–2.86). Most of the study taxa exhibit deeper phylogeographic structuring within the eastern region, although *C. angulata* harboured deeper divergences within the western region. The DENIM analyses were less well‐supported, but demonstrated the same phylogeographic division between eastern and western clades, although *H. areolatus* and *A. meleagris* showed slight changes in the structure of the eastern clades (Figure S3A–G). The divergence time estimates retrieved from the DENIM analyses were generally more recent, although the *Potamonautes* species displayed earlier divergence time estimates.

### 3.2 Population genetic analyses

The hierarchical AMOVA results demonstrated high levels of genetic structuring across all of the study taxa (Appendix S3: Table S3.4). The greatest percentage of differentiation was consistently found to be among the western and eastern populations (48.69%–86.22%), with similarly high fixation indices (*F*_CT_ = 0.49–0.86). Variation among localities (10.24%–42.52%; *F*_SC_ = 0.62–0.91) was also found to be consistently greater than variation within localities (2.21%–16.29%; *F*_ST_ = 0.84–0.98).

The AMOVA results within each region demonstrated significantly higher variation among localities (57.29%–96.44%) for all species for both regions compared with within localities (3.56%–37.01%) excluding the western clades of *H. areolatus* and *D. lutrix*, which returned insignificant *F*_ST_ values (Appendix S3: Table S3.5). The two western *Potamonautes* species, *P. parvicorpus* and *P. perlatus*, displayed markedly similar levels of variation among localities (96.44% vs. 95.35%) and within localities (3.56% vs 4.65%) for the COI locus. Variation between localities was generally greater in the eastern region, with *H. areolatus*, *D. lutrix* and *A. meleagris* each displaying high levels of variation compared with the western region. Similarly, the two eastern *Peripatopsis* species, *Per. lawrencei* and *Per. overbergiensis*, also displayed greater levels of variation compared with the western *Per. capensis* species. The two western *Potamonautes* species, however, displayed greater levels of variation (Va = 71.6%–96.44%; *F*_ST_ = 0.72–0.96) than the four eastern *Potamonautes* species (Va = 50.5%–81.35%; *F*_ST_ = 0.65–0.90). *Chersina angulata*, meanwhile, displayed comparable levels of variation in both the western (Va = 61.13%–68.10%; *F*_ST_ = 0.61–0.67) and eastern (Va = 57.29%–72.30%; *F*_ST_ = 0.63–0.72) regions.

Appendix S3: Table S3.6 shows the standard diversity indices and neutrality test results for each mitochondrial locus used in the study per clade. *Potamonautes perlatus* demonstrated the lowest haplotype diversity overall (h = 0.695), as well as the lowest nucleotide diversity (π = 0.004). *Peripatopsis lawrencei* displayed the highest haplotype diversity (h = 0.983), while *P. barnardi* demonstrated the highest nucleotide diversity (π = 0.094). The neutrality tests were generally not significant, suggesting that there were no major deviations from the expected equilibrium. However, statistically significant negative values were calculated for the main western clade of *H. areolatus* as well as both western clades for the cytb locus of *C. angulata*, indicating either a recent incidence of positive selection or a population expansion after a recent bottleneck. The combined species *P. brincki* + *P. parvicorpus* + *P. tuerkayi*, conversely, shows a significant positive value for the COI locus, indicating a sudden population contraction.

### 3.3 Phylogeographic concordance factors

The phylogeographic concordance factor analyses revealed broadly congruent patterns of cladogenesis among six of the studied taxa, namely *H. areolatus*, *D. lutrix*, *A. meleagris* and (*P. perlatus* + *P. barbarai* + *P. barnardi*; Figure 1a,b), indicating a clear east/west division which echoes the east/west phylogeographic division evidenced by the previous BEAST analyses. Removal of *C. angulata* from the analyses resulted in high basal nodal support values (>0.71), as well as high overall levels of congruence (>0.71) (Appendix S3: Table S3.22). *Potamonautes brincki*, *P. parvicorpus*, *P. tuerkayi*, *Peripatopsis capensis*, *Per. overbergiensis* and *Per. lawrencei* were excluded from this analysis due to the fact that the PCF approach requires samples to be present in each delineated region. Consequently, a number of subsequent analyses were run, clustering together proximate localities (<10 km apart). Of the six pairwise analyses which were run, two were shown to support congruent phylogeographic patterns (PCF ≥0.71), namely *P. brincki* + *Peripatopsis* spp. (PCF = 0.75; Figure S1A) and *D. lutrix* + (*P. brincki* + *P. parvicorpus* + *P. tuerkayi*) (PCF = 0.89; Figure S1B). Although the *D. lutrix* + (*Per. capensis* + *Per. lawrencei*) comparison did not support an overall congruent divergence (PCF = 0.57; Figure S1C) likely due to the inclusion of additional sampling localities, the clade consisting of localities found throughout the Table Mountain Reserve showed a high degree of congruence between the two species (PCF = 0.93). Similarly, while the *D. lutrix* + *A. meleagris* comparison did not show an overall congruence (PCF = 0.44; Figure S1E), the clade consisting of the easternmost localities (Grahamstown and Port Alfred) showed a high level of congruence between the taxa (PCF = 0.84).

### 3.4 Test for synchronous divergence

**FIGURE 3:**
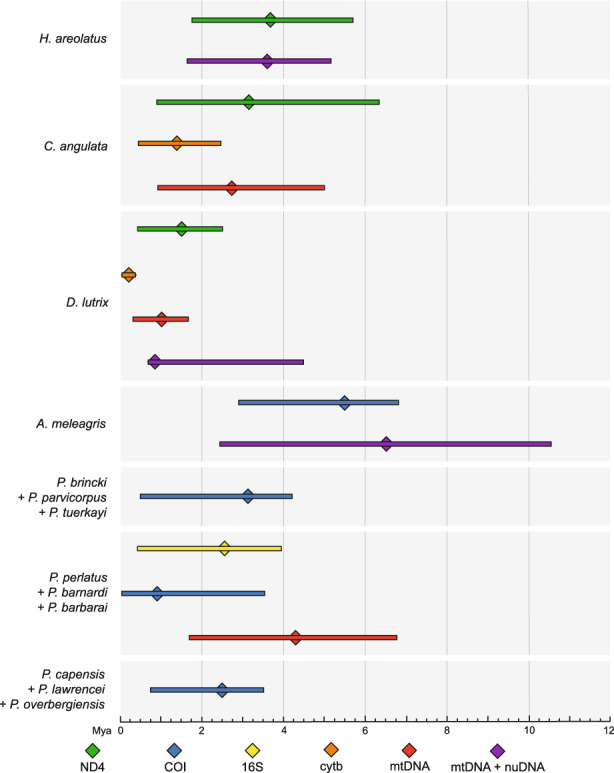
*BEAST divergence time estimations between the western and eastern phylogeographic regions based on single locus datasets, as well as combined mtDNA and total evidence datasets where available. The central diamonds indicate the mean divergence time estimate, while the bars indicate the 95% highest posterior densities.

**FIGURE 4:**
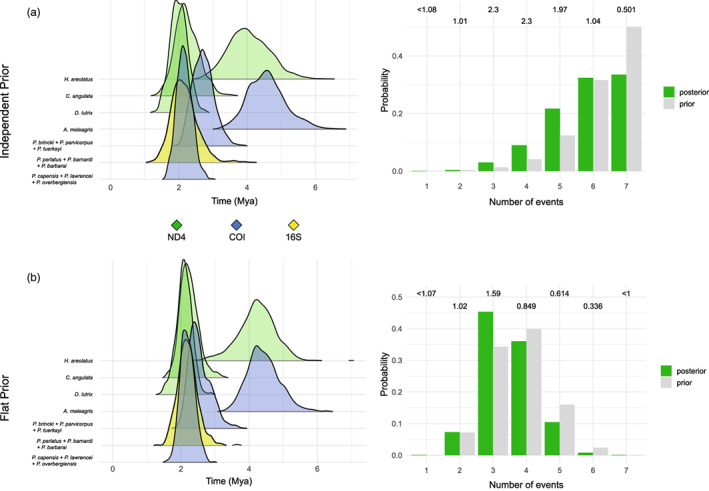
Simultaneous divergence results based on single mtDNA loci as determined by ecoevolity, using both ‘independent’ (a) and ‘flat’ (b) priors. Marginal distribution plots of divergence time in million years are shown on the left, while the bar graphs on the right show the posterior and prior probabilities of the number of events with the corresponding Bayes factors above the bars.

The *BEAST analyses revealed both variation and overlap in the divergence times between the western and eastern phylogeographic regions (Figure 3). *Homopus areolatus*, *C. angulata*, *P. brincki* and *Per. capensis* all seem to have diverged between 2 and 4 Mya based on separate analyses using a single mtDNA locus, combined mtDNA data, as well as a total evidence approach where possible. While the use of the 16S mtDNA locus for *P. perlatus* also places the taxon neatly in this time frame and suggests a simultaneous divergence with the above taxa, the inclusion of the COI mtDNA locus generates a more distant mean divergence estimation, as well as a broader 95% HPD. *Duberria lutrix* demonstrates a more recent divergence (~1.2 Ma) using both a single gene, combined mtDNA and total evidence approach. The ecoevolity analyses generally retrieved more recent divergence time estimates and did not support either complete synchronous or asynchronous divergence (Figure 4a,b). Analyses conducted using a single mtDNA locus per taxon using the ‘independent’ prior showed high support for three (BF = 2.302) independent divergence events (Figure 4a; Appendix S3: Table S3.21A). Analyses using the ‘flat’ prior also showed high support for three divergence events (BF = 1.587) (Figure 4b; Appendix S3: Table S3.21B), with the likeliest model (pP = 0.09, BF = 86.56) indicating that *H. areolatus* and *A. meleagris* diverged simultaneously ~4.5 Mya, followed by *P. brincki* ~ 2.5 Mya, and lastly *C. angulata*, *D. lutrix*, *P. perlatus* and *Per. capensis* ~2 Mya.

### 3.5 Climatic niche modelling

The bioclimatic variable correlation analyses based on the presence data for each taxon resulted in largely different sets of variables being used to construct the species distribution models of each species (Figure S4A–G). All of the species distribution models performed well, with consistently high AUC_TEST_ and AUC_TRAIN_ scores, indicating good model discrimination ability (Appendix S3: Table S3.9). The resulting variable contributions based on permutation importance (Figure S5A–G) indicated that the modelled species distributions of the seven terrestrial ectotherms were predominantly influenced by the Annual Mean Temperature (Bio1), the Temperature of the Coldest Month (Bio6) and the Mean Temperature of the Wettest Quarter (Bio8), whereas the six crab species were influenced by both the Precipitation of the Coldest Quarter (Bio19) and Isothermality (Bio3). The SDMs for each species were differentially affected by past paleoclimatic changes leading up to the present (Figure 6). While the suitable environment for *C. angulata* and *D. lutrix* remained relatively stable since the last interglacial period, *H. areolatus*, *P. brincki*, *P. parvicorpus*, *P. tuerkayi* and the three velvet worm species saw progressive habitat contraction and fragmentation. Several of the SDMs indicated that the central CFM region is either currently unsuitable for habitation (e.g. *H. areolatus*) or has previously acted as an inhospitable area (e.g. *A. meleagris*). Of the two paleoclimatic models used in this study, the CCSM4 model generally resulted in far broader distributions, particularly during the LGM period. All of the study taxa other than *H. areolatus* and *A. meleagris* were shown to have continuous distributions since the last interglacial period, with the SDMs resolving broad areas of stable climatic suitability for most species along the southern and southwestern coasts (Figure S6A–G). Intersecting these areas resulted in a small stretch of coastline along the Cape, which has remained suitable for all of the taxa in this study (Figure S6H).

### 3.6 Climatic niche comparison

**FIGURE 5:**
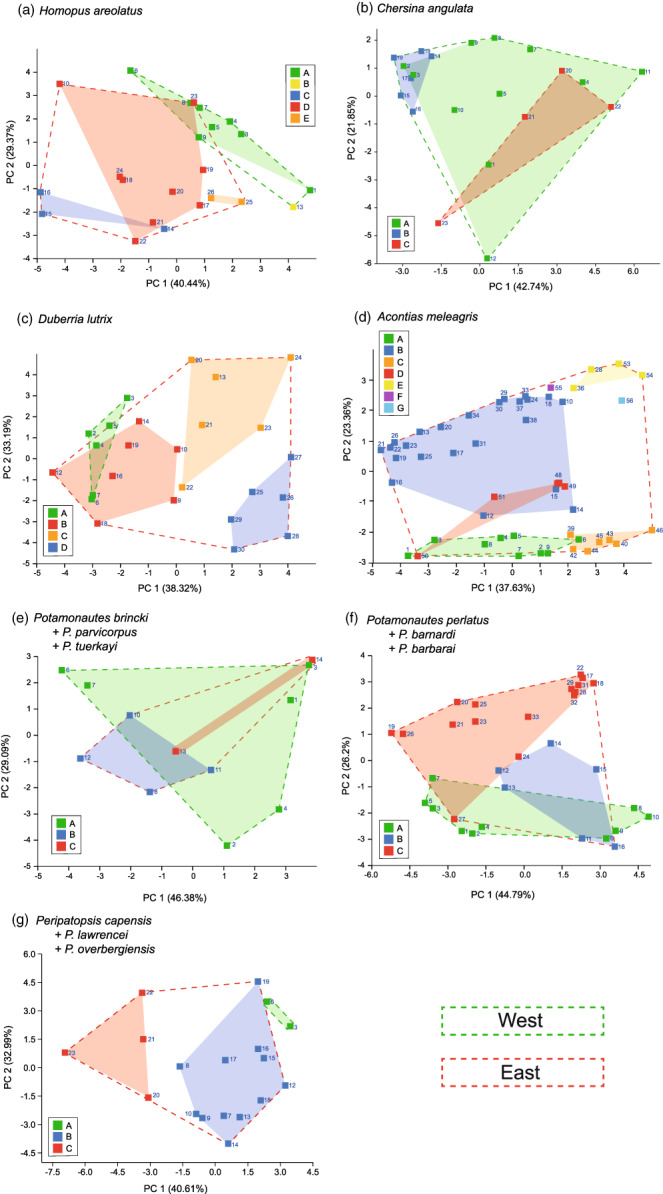
Principal component analysis using the climatic variables at the sampling localities of each species (a–g). Localities were rarefied to include only one datapoint per 5 km^2^. Dashed lines indicate the western (green) and eastern (red) regions, while the solid colours and locality numbers correspond to the clades and localities shown in Figure 2a–g.

The PCA on the bioclimatic variables at the sampling localities (Figure 5a–g) showed minimal overlap between the populations of each species within the eastern phylogeographic region as well as between the two regions, although the eastern and western phylogeographic regions did overlap for *C. angulata* and the *Potamonautes* species. The first two principal components for each taxon explained between 63.99% and 78.04% of the total variance (Appendix S3: Tables S3.10a–S3.16a). The PERMANOVA results demonstrated that the bioclimatic variables were significantly different across both populations as well as phylogeographic regions among the majority of the study taxa, although the three velvet worm species showed no significant difference between the regions and (*P. brincki* + *P. parvicorpus* + *P. tuerkayi*) showed no differences between the regions nor the populations (Appendix S3: Tables S3.10c–S3.16c). The Kruskal–Wallis tests showed that the number of bioclimatic variables which were significantly different among populations varied considerably between taxa (Appendix S3: Tables S3.10e–S3.16e), with precipitation variables (Bio12–Bio19) more frequently differing between populations than temperature variables (Bio1–Bio11), and with Bio15 (precipitaition seasonality) displaying significant differences between populations in all taxa (Table S3.17). Several of the precipitation variables demonstrated significant correlations with longitude (Spearman's correlation; Appendix S3: Tables S3.10b–S3.16b), with the eastward shift from a winter rainfall region to a year‐round rainfall region reflected in significantly decreased precipitation seasonality (Bio15), as well as increased precipitation in the driest (Bio17) and warmest (Bio18) quarters (Appendix S3: Table S3.18).

### 3.7 Abiotic associations with genetic structure

The redundancy analyses (Appendix S3: Table S3.19) all demonstrated that the ‘full’ models (Climate + Distance + CFM) explained the highest percentage of genetic variation across all of the study taxa (74.72%–98.08%). Of the pairwise combined models, the ‘CFM + Climate’ model explained the greatest proportion of variation (24.49%–70.71%), followed by the ‘Distance + Climate’ model (14.66%–65.96%). Climate was shown to be the most significant single explanatory variable in all of the taxa studied (9.36%–40.69%), followed by the CFM barrier (0.49%–9.14%) and finally geographic distance (0.35%–5.11%). All of the models were found to be statistically significant, although the ‘CFM’ model for *P. brincki* was shown to not have a constrained component.

## 4 DISCUSSION

Most of the taxa in the present study exhibited two discrete phylogeographic regions, comprised of an eastern and western clade (Figures 1 and 2a–g). Two of the reptiles, *Chersina angulata* and *A. meleagris*, were notable exceptions, with the former displaying a recent easterly range expansion through the CFM syntaxis zone and the latter featuring a phylogenetically eastern but geographically western clade, indicative of a secondary dispersal event across the CFM (Engelbrecht et al., 2013). The east/west division was less apparent in the montane invertebrates including the freshwater crabs *P. brincki* + *P. parvicorpus* + *P. tuerkayi* and the three forest‐dwelling velvet worm *Peripatopsis* species, the distributions of which include within the CFM syntaxis zone itself. These observations imply the presence of multiple lineage‐specific drivers of cladogenesis within the region, which may act in concert with the geographical barrier to gene flow posed by the CFM. A lack of complete temporal congruence in divergence time estimations between these two regions is likely indicative of species‐specific responses to ancient climatic fluctuations (Figures 3 and 4). The taxa therefore share a pseudocongruent phylogeographic history, with geographic features and climatic gradients both influencing cladogenesis within the GCFR taxa.

### 4.1 Spatial and temporal congruence

The PCF analyses revealed broadly congruent patterns of cladogenesis among four of the studied taxa, namely *H. areolatus*, *D. lutrix*, *A. meleagris* and the combined species *P. perlatus* + *P. barnardi* + *P. barbarai* (Figure 1a,b), indicating a clear east/west division among basal nodes along the Hottentots Holland Mountains in the CFM syntaxis zone, as well as a secondary north/south division along the Langeberg and Overberg (nodal values marked with ‘*’). Patterns within the eastern clades indicate an earlier division between the northern region D and the southern regions, with regions B and C displaying higher levels of relatedness. This may be indicative of an overall eastward dispersal of species within the GCFR, with populations north of the CFM becoming subsequently isolated. *Chersina angulata* did not fit this pattern, due to the occurrence of populations on the eastern side of the HHM which contain mtDNA haplotypes belonging to the western clade, and the inclusion of this species reduced the observed phylogeographic congruence (uppermost nodal values). The presence of these haplotypes in the eastern region is most likely due to a recent range expansion (Daniels et al., 2007), which is supported by the significantly negative Fu's *Fs* values retrieved for both western clades. The *Potamonautes* species demonstrated a unique pattern of divergence within the eastern clade, and the removal of this species from the analysis increased the levels of congruence observed in the remaining species (nodal values marked ‘**’). This analysis excluded the secondary dispersal event observed in *A. meleagris* (Figure 2d, clade C) (Engelbrecht et al., 2013), as this was not observed in the other taxa and would have erroneously grouped together two geographically proximate but genetically divergent clades. The basal concordant divergence contrasts markedly with the more recent patterns of divergence observed among the study taxa, which is indicative of a shared mechanism separating the eastern and western phylogeographic regions within the GCFR, followed by species‐specific responses to each region (Figures 1 and 2a–g; Figures S2 and S3). This is likely due to differences in niche requirements, competition and dispersal ability between the study taxa subsequent to their shared divergence across the CFM.

The subsequent individual pairwise analyses based on geographically proximate localities (Figure S1A–F) again supported the shared east/west division across the HHM and indicated high levels of fine‐scale phylogeographic congruence between *P. brincki* + *Per. lawrencei* + *P. tuerkayi* and the three *Peripatopsis* species. Additionally, *P. brincki* + *Per. lawrencei* + *P. tuerkayi* and *D. lutrix* were also shown to exhibit high levels of phylogenetic congruence, although these results should be interpreted with care due to the limited number of sampling localities. All of the analyses which included sampling localities in the Cape Peninsula (Figure S1A–C) indicated high levels of support for a genetic division between this area and Stellenbosch/Somerset West region, most likely due to their separation by a 60 km stretch of land known as the Cape Flats region (Wishart & Hughes, 2001), an area shown to act as a barrier to dispersal for several other invertebrate taxa (Daniels et al., 2001; Gouws et al., 2004, 2010; Wishart & Hughes, 2001, 2003).

Eastern clades generally displayed significantly greater levels of phylogeographic structuring than western clades (Figure 2a–g), with most taxa demonstrating only a single western clade, and several highly supported eastern clades. Indeed, work by Daniels et al. (2001) and Wood and Daniels (2016) concluded that the eastern clades of *P. brincki* were sufficiently divergent to constitute separate species, a conclusion which was similarly reached for both *Per. capensis* (McDonald & Daniels, 2012) and *P. perlatus* (Phiri & Daniels, 2014). *Chersina angulata* was a notable exception to this pattern, with two western clades separated along a north–south axis, and only a single eastern clade. Historical climatic fluctuations have had a major impact on the direction and structuring of the Berg River drainage system, with the path of the Berg River shifting progressively northward during the Mio/Pliocene (Hendey, 1983). Additionally, during major past regressions, the Berg and Olifants rivers were probably joined into a single river system (Hendey, 1983). These changes may have served to periodically isolate south‐ and northwestern populations of *C. angulata*, resulting in the observed phylogeographic structuring.

Most species did not share haplotypes between regions, suggesting the presence of a barrier to gene flow (Figure S2). This is supported by the AMOVA (Appendix S3: Table S3.4), which generally retrieved high fixation indices indicative of limited dispersal. *Chersina angulata* and *H. areolatus* displayed lower fixation values among localities, likely due to their larger size and dispersal ability (Jenkins et al., 2007), which is supported by significantly negative Fu's *F*s values. In contrast, the high Tajima's *D* and Fu's *F*s values observed in the combined *P. brincki* + *Per. lawrencei* + *P. tuerkayi*, which are characteristic of a recent range contraction or bottleneck, indicate the presence of species‐specific demographic histories in the region. This is corroborated by the results of the comparative phylogeographic analyses, which provide strong evidence for several independent divergence events among the focal taxa (Figure 4a,b, Figure S7A–C) and therefore asynchronous cladogenesis within the GCFR. However, the ecoevolity results did indicate high support for shared, synchronous divergence events among *C. angulata*, *D. lutrix*, *P. perlatus* and the three *Peripatopsis* species ~2 Mya, as well as *H. areolatus* and *A. meleagris* ~4.2 Mya. Both of these estimates coincide with periods of Antarctic cooling during the Neogene (1.8–2.4 Mya and 3.2–4.2 Ma) (Harwood, 1985), the latter of which coincided with Antarctic ice sheet development 3–4 Mya (deMenocal, 2004; Diekmann et al., 2003; Marlow et al., 2000) as well as continental uplift throughout the southern margin of South Africa 3–5 Mya (Linder, 2003) suggesting shared responses to climatic oscillations in GCFR.

### 4.2 The CFM as a driver of cladogenesis

The common phylogeographic division between eastern and western clades among all taxa (Figure 2a–g), synchronous divergence of two groups of the taxa (Figure 4a,b), general absence of shared mtDNA haplotypes between the two regions (Figure S2) and general unsuitability of the HHM to habitation (Figure S6A–G) all indicate that the CFM pose a significant barrier to gene flow within the GCFR.

The CFM are ancient, resulting from the folding and weathering of sediments deposited 510–330 Mya (Compton, 2004), and attain a maximum elevation of 2249 m (Verboom et al., 2015), with the altitude of the southern HHM range fluctuating between 500 and 1590 m. The southwestern GCFR is thought to have been tectonically stable through the Plio/Pleistocene (Verboom et al., 2015), and thus, the HHM have remained a significant geographic feature throughout the time frame of interest in the current study. The rapid change in elevation from the low‐lying areas abutting the CFM could represent a significant barrier to dispersal (Kulenkampff et al., 2019), with similar phylogenetic breaks having been observed in other codistributed reptile species (Barlow et al., 2013; Daniels et al., 2007; Swart et al., 2009; Tolley et al., 2008), invertebrate species (Price et al., 2007), as well as some mammalian species (Smit et al., 2007; Willows‐Munro & Matthee, 2011). However, the recent range expansion of *C. angulata* indicates that this barrier may pose less of a challenge to some species and may serve to limit rather than prevent gene flow.

The CFM may have also played a causal role in other factors which influence the phylogeographic structure of taxa, such as drainage systems, climatic patterns and vegetation types. The CFM syntaxis zone represents the interface between western and southern flowing drainage systems, serving to delineate the Eerste and Berg River catchment areas in the west, and the Breede and Bot River catchments to the southeast (Watson et al., 2021). Price et al. (2010) highlighted the significant impact which watersheds and catchments can play in cladogenesis even in the absence of geographic features as dramatic as the CFM, and high mountain streams have been frequently shown to demonstrate high levels of species richness in invertebrates, often with distinct biota in discrete mountain catchments (Daniels, 2003; Gouws et al., 2004, 2010; Stewart, 1992; Wishart & Hughes, 2001). Indeed, the species *P. perlatus* + *P. barbarai* + *P. barnardi* were found to be genetically separated into southern and western flowing drainage systems (Phiri & Daniels, 2014), and thus, the influence of the CFM on drainage system flow direction may represent a major biogeographic barrier to inland aquatic fauna.

The syntaxis zone of the CFM acts as an environmental transition zone, broadly separating the winter rainfall region in the west, and the all‐year rainfall region in the east (Bradshaw & Cowling, 2014; Cowling et al., 2009; Siesser, 1980; Tolley et al., 2009). While this pattern is influenced by a number of complex factors, the steep elevation of the CFM prevents the eastward movement of cold fronts during the winter months, reducing winter rainfall to around 50% beyond the CFM and resulting in orographic effects, leading to high rainfall (up to 3000 mm) in the upper peaks of the HHM. Differences in rainfall regimes have been implicated in the division of some reptilian clades (Kulenkampff et al., 2019; Tolley et al., 2009, 2014) and the RDA analyses in the present study consistently identified climate as the highest single abiotic variable associated with genetic variance across all taxa (Appendix S3: Table S3.19). The environmental and topographical heterogeneity across the CFM has contributed towards the presence of a variety of different vegetative biomes, with Southwestern Fynbos within the syntaxis zone itself bordered by East Coast and West Coast Renosterveld in the abutting lowland regions (Bergh et al., 2014). As some invertebrate species have been shown to display high levels of habitat specificity (Potgieter, 2013; Price et al., 2007; Tolley et al., 2004, 2006, 2008), this vegetative transitional zone within the CFM may present a significant barrier to gene flow between the eastern and western phyloregions.

### 4.3 Climatic refugia

Cladogenesis within the GCFR is often attributed to the pattern of past climatic oscillations throughout the Mio/Plio/Pleistocene, with ectothermic species experiencing repeated cycles of range expansion and contraction (Tolley et al., 2014), often retreating from the coast to intermontane refugial areas during periods of unfavourable climatic conditions (Daniels et al., 2007; Kulenkampff et al., 2019) or marine transgressions (Partridge & Maude, 1987), exploiting novel, arid coastal plains (Engelbrecht et al., 2013) or becoming isolated due to contractions in river systems (Daniels et al., 2001). The effects of these climatic changes on taxa are thus largely dependent on their specific life histories and habitat requirements, with a range of responses complicating the search for shared patterns of diversification.

Due to the different environmental niche requirements of each taxon in this study, the bioclimatic variables which displayed high levels of correlation differed (Figure S4A–G), resulting in different sets of variables being included in the construction of each SDM (Figure S5A–G), making direct comparisons as to the importance of particular variables difficult. However, similarities in more closely related taxa were evident. *Homopus areolatus* and *C. angulata*, both members of the family Testudinidae, retrieved high levels of permutation importance (PI) for the Mean Temperature of the Wettest Quarter (Bio8), while all taxa from the genus *Potamonautes* were shown to be highly dependent on the Minimum Precipitation of the Coldest Month (Bio19), likely due to their reliance on the presence of freshwater rivers habitats.

**FIGURE 6:**
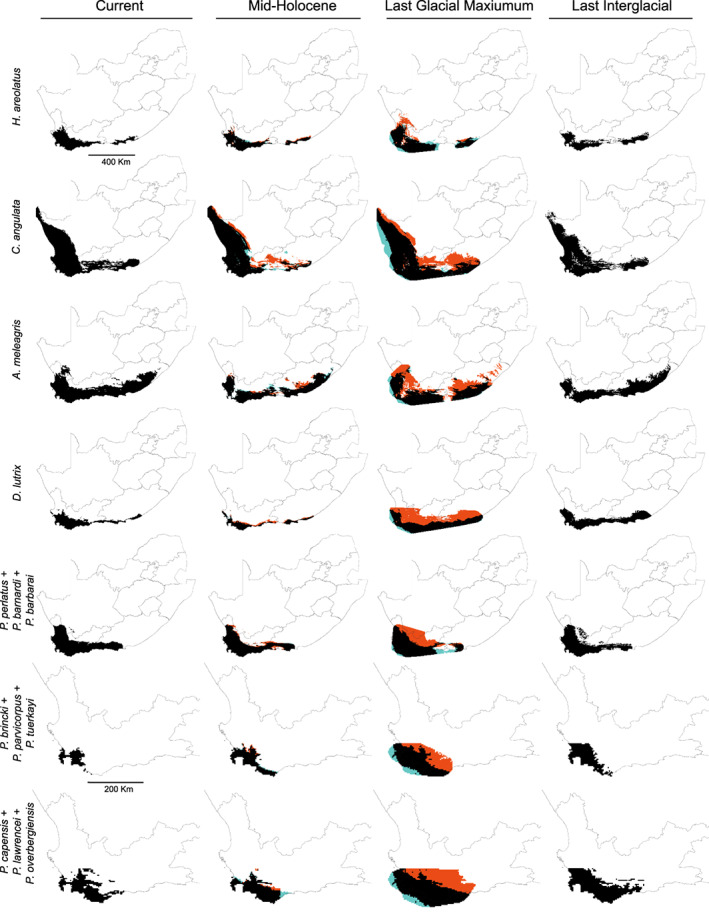
Results of the species distribution models in binary form based on the cloglog 10% omission rate threshold for each taxon under both current and paleoclimatic conditions. MH and LGM distributions were based on both MIROC‐ESM (blue) and CCSM4 (orange) models, with their intersections indicated in black.

Despite building the SDMs from different sets of variables, all of the vertebrate taxa models demonstrated a common region of unsuitable habitat which aligns with the location of the HHM and serves to separate the distribution of the taxa into western and eastern units (Figure S6A–G), particularly during paleoclimatic conditions (Figure 6). Furthermore, while all taxa other than *H. areolatus* displayed continuous distributions under current conditions, all SDMs of the widely sampled taxa displayed fragmented species‐specific refugial regions with discontinuities which broadly correspond to their respective genetic lineages, a result which suggests that paleoclimatic conditions may have isolated populations from one another, driving cladogenesis. Additionally, the SDMs demonstrated that the Agulhas plain, a region stretching southward from the HHM which would have been exposed during periods of reduced sea levels such as the LGM and late Pliocene (Siesser & Dingle, 1981), constituted suitable habitat for all of the taxa in the present study during the LGM, supporting the hypothesis that climatic fluctuations would have facilitated the dispersal of species across the exposed southern coastline around the southern extent of the CFM (Engelbrecht et al., 2013; Hofmeyr et al., 2020; Kulenkampff et al., 2019; Schreiner et al., 2013), resulting in the shared pattern of divergence observed across all taxa.

The intersection of the species‐specific refugial areas (Figure S6H) identified a climatically stable region consisting of a number of fragmented patches along the southwest coastline, including the southern tip of the Cape Peninsula, the Cape Flats, the coastal stretch along the southern syntaxis zone of the CFM from Pringle Bay to Hermanus, the Danger Point Peninsula, and finally the Quorin Point Nature Reserve. However, this result is restricted to the overlap of the distributions of all the focal taxa in this study and therefore excludes climatically stable areas in the eastern GCFR. Due to their climatic stability, these areas should be investigated further and prioritized for future conservation efforts.

### 4.4 Climatic niche differences

The GCFR displays high levels of landscape and climatic heterogeneity (Cowling & Lombard, 2002). The western winter rainfall region is marked by high levels of seasonality driven by circum‐polar cyclonic air masses (Preston‐Whyte & Tyson, 1989) delivering rain during the cooler months, before succumbing to the intensification of the South Atlantic high‐pressure cell during the warmer months, while the eastern regions transition into a progressively year‐round rainfall regime with increasing longitude, driven by both the Cape Fold Belt and the warmer Indian Ocean beyond Cape Agulhas (Bradshaw & Cowling, 2014).

This strong longitudinal climatic gradient (Appendix S3: Tables S3.10b–S3.16b and S3.18) results in a progressive eastwards reduction in both temperature variability and precipitation seasonality and contributes towards both an overall climatic shift between the western and eastern regions within the GCFR (Appendix S3: Tables S3.10C–S3.16C), as well as a generally distinct climatic distribution among clades, particularly between those in the eastern GCFR (Figure 5a–g). As the sampling design of each of the studies which generated the sequence data used in the present study was determined by the abundance of each taxon, the differences in bioclimatic variables observed between populations are likely a reflection of their respective environmental niches.

Environmental gradients have been shown to induce differentiation due to local adaptations even in populations, which have adjacent ranges and which lack major geographical barriers to gene flow (Doebeli & Dieckmann, 2003; Rainey & Travisano, 1998). The establishment of this rainfall gradient is thought to have occurred during the late Miocene roughly 10 Mya (Diekmann et al., 2003; Krammer et al., 2006; Siesser, 1980), prior to the divergence time estimates of the taxa in this study, and so would likely have played a role in niche differentiation throughout the Plio/Pleistocene. Indeed, climate was shown to be the single most important explanatory variable contributing towards genetic variance in every taxon (Appendix S3: Table S3.19), with precipitation seasonality in particular consistently associated with genetic variation in all of the study taxa (Appendix S3: Tables S3.10e–S3.16e and S3.17).

In concert with this climatic gradient, variation in rock and soil types (Bradshaw & Cowling, 2014), as well as marked differences in vegetation (Bergh et al., 2014), has produced a mosaic of ecological niches throughout the GCFR, and it is likely that the populations within each species would have adapted to the specific environmental niches present within their distributions.

### 4.5 Limitations and future directions

The SDMs generated in this study were based on a spatial resolution of 2.5 arc minutes (roughly 5 km × 5 km), which have been used extensively in previous studies (Busschau et al., 2022; Hofmeyr et al., 2020; Spitzweg et al., 2020). While this resolution was sufficient to elucidate the broad‐scale regional trends of interest in this study, a finer‐resolution may have reflected a more accurate picture of the CFM syntaxis zone, given the high levels of climatic and landscape heterogeneity in the region. The SDMs of small‐bodied taxa displaying low levels of vagility, such as *Per. capensis* or *P. brincki*, may have benefitted from this approach, given their distributions within the HHM themselves. The data utilized in this study were sourced from preexisting work and, as such, the sampling regime was not designed with the goals of this study in mind. Specifically, the studies upon which this paper is based did not account a comparative phylogeographic approach and therefore were not sampled across the same distributions, resulting in some species being excluded from the PCF analyses. Future studies would benefit from further fine‐scale sampling around the east/west transitional zone of the CFM within the GCFR in order to isolate the effects of the HHM themselves from confounding variables such as climate and to more accurately gauge the impacts of climatic fluctuations on population‐level responses.

Most of the fine‐scale sampling in this study included only mtDNA loci, as nuclear DNA data were unavailable for every species, or were only sequenced for one individual per sampling locality. Divergence times can vary among genes, thereby introducing uncertainty into the divergence time estimates based on single loci, although this variation should reduce when the number of generations between divergence events is large relative to the effective population size (Thorne & Kishino, 2002). Furthermore, while the expected variation in mutation rates each marker would exhibit due to differential selective pressures across the climatic gradient in the GCFR may introduce error, the relatively recent time scale of the divergences in this study may buffer against these differences. It is also important to make the distinction between gene divergence and population divergence, the latter of which almost always occurs first (Edwards & Beerli, 2000). While this effect may impact the accuracy of the absolute divergence time estimates per taxon, the simultaneous divergence analyses compare relative divergence between taxa and therefore should not be impacted by this discrepancy. While the current study has attempted to utilize preexisting data, the inclusion of rapidly evolving nuDNA loci or genomic data would allow for a more accurate assessment of estimations of gene flow, population divergence patterns and divergence time estimates.

Finally, this study focussed entirely on ectothermic organisms and may not be generalizable to other organisms such as birds, mammals and flora. The inclusion of these types of organisms in future studies may provide a clearer picture of the effect of the CFM as a barrier to gene flow and, thus, as a diver of cladogenesis.

## 5 CONCLUSION

The present study highlights the complex interactions between the CFM within the GCFR and past climatic oscillations during the Plio/Pleistocene, resulting in both lineage‐specific responses as well as pseudocongruent phylogeographic patterns. The congruent east/west phylogeographic division observed in all taxa lends support to the conclusion that the longitudinal climatic gradient within the GCFR, mediated in part by the barrier to dispersal posed by the CFM, plays a major role in lineage diversification and population differentiation.

## CONFLICT OF INTEREST

The authors have no competing interests to declare.

## Supporting information


Figure S1

Figure S2

Figure S3

Figure S4

Figure S5

Figure S6

Figure S7

Appendix S1

Appendix S2

Appendix S3

## Data Availability

The data used in this study are freely available on GenBank (https://www.ncbi.nlm.nih.gov/genbank/), with accession numbers as indicated in Appendix S2: Table S2. The climate data and MaxEnt input files used are available on Dryad (https://doi.org/10.5061/dryad.cc2fqz68k) [The reviewer access link is https://datadryad.org/stash/share/bR0eHmattQPmsAcvvorch66reg‐2n2pEO36UFrPrwpY].
