# Supplementary material for: Between the Cape Fold Mountains and the deep blue sea: Comparative phylogeography of selected codistributed ectotherms reveals asynchronous cladogenesis
Source: Evol Appl. 2022 Oct 27;15(12):1967–87. doi: 10.1111/eva.13493 (PMC9753840; doi:10.1111/eva.13493)
Supplement: Supplementary file 1 — Figure S1 [file EVA-15-1967-s004.pdf]

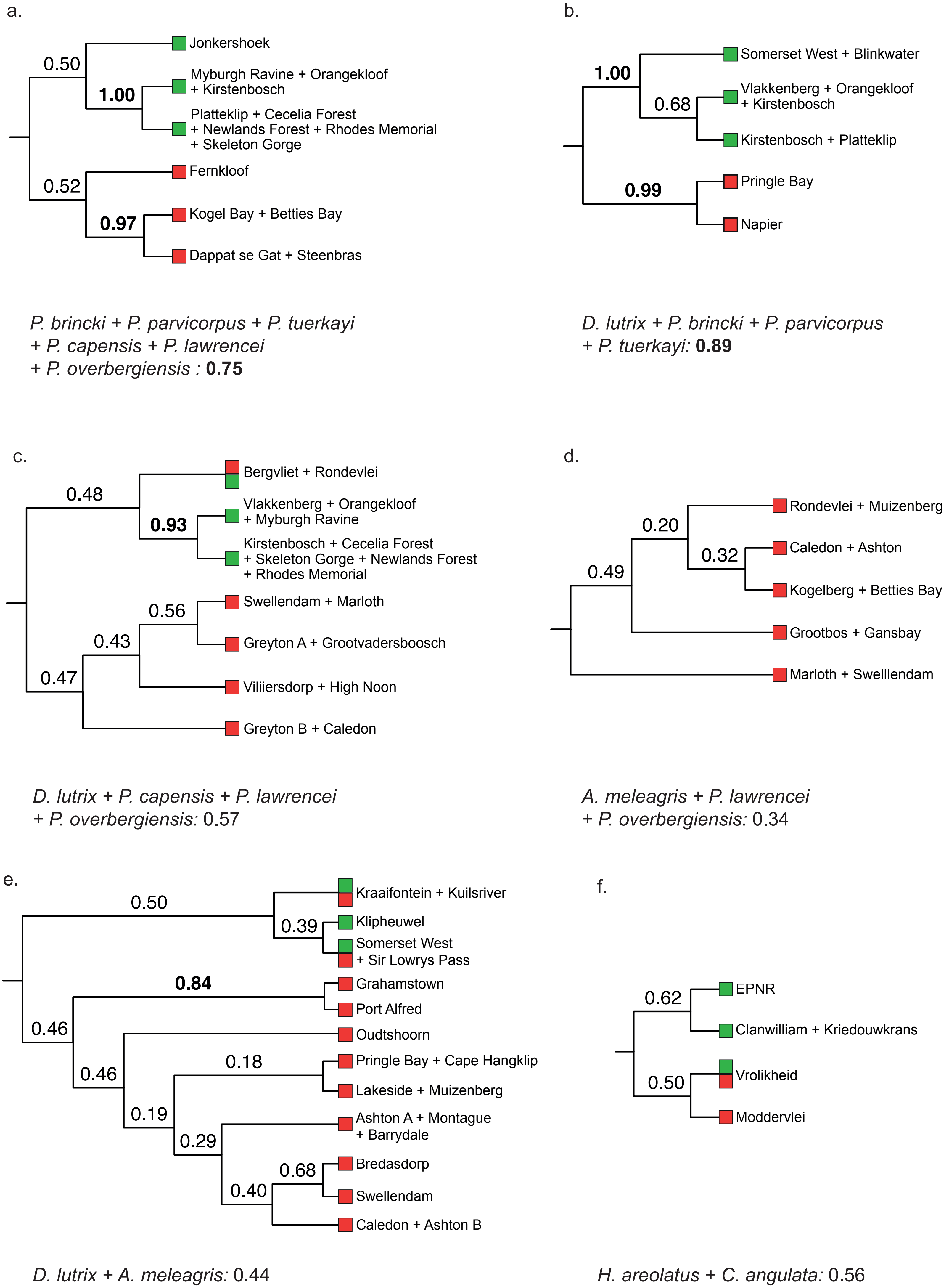
a.
0.50
Jonkershoek
1.00
Myburgh Ravine + Orangekloof + Kirstenbosch
Platteklip + Cecelia Forest + Newlands Forest + Rhodes Memorial + Skeleton Gorge
0.52
Fernkloof
0.97
Kogel Bay + Betties Bay
Dappat se Gat + Steenbras
P. brincki + P. parvicorpus + P. tuerkayi + P. capensis + P. lawrencei + P. overbergiensis : 0.75
b.
1.00
Somerset West + Blinkwater
0.68
Vlakkenberg + Orangekloof + Kirstenbosch
Kirstenbosch + Platteklip
0.99
Pringle Bay
Napier
D. lutrix + P. brincki + P. parvicorpus + P. tuerkayi: 0.89
c.
0.48
Bergvliet + Rondevlei
0.93
Vlakkenberg + Orangekloof + Myburgh Ravine
Kirstenbosch + Cecelia Forest + Skeleton Gorge + Newlands Forest + Rhodes Memorial
0.56
Swellendam + Marloth
0.43
Greyton A + Grootvadersboosch
0.47
Viliiersdorp + High Noon
Greyton B + Caledon
D. lutrix + P. capensis + P. lawrencei + P. overbergiensis: 0.57
d.
0.20
Rondevlei + Muizenberg
0.32
Caledon + Ashton
0.49
Kogelberg + Betties Bay
Grootbos + Gansbay
Marloth + Swelllendam
A. meleagris + P. lawrencei + P. overbergiensis: 0.34
e.
0.50
Kraaifontein + Kuilsriver
0.39
Klipheuwel
Somerset West + Sir Lowrys Pass
0.84
Grahamstown
Port Alfred
0.46
Oudtshoorn
0.46
0.18
Pringle Bay + Cape Hangklip
Lakeside + Muizenberg
0.19
Ashton A + Montague + Barrydale
0.29
0.68
Bredasdorp
Swellendam
0.40
Caledon + Ashton B
D. lutrix + A. meleagris: 0.44
f.
0.62
EPNR
Clanwilliam + Kriedouwkrans
0.50
Vrolikheid
Moddervlei
H. areolatus + C. angulata: 0.56
