# Supplementary material for: Between the Cape Fold Mountains and the deep blue sea: Comparative phylogeography of selected codistributed ectotherms reveals asynchronous cladogenesis
Source: Evol Appl. 2022 Oct 27;15(12):1967–87. doi: 10.1111/eva.13493 (PMC9753840; doi:10.1111/eva.13493)
Supplement: Supplementary file 2 — Figure S2 [file EVA-15-1967-s010.pdf]

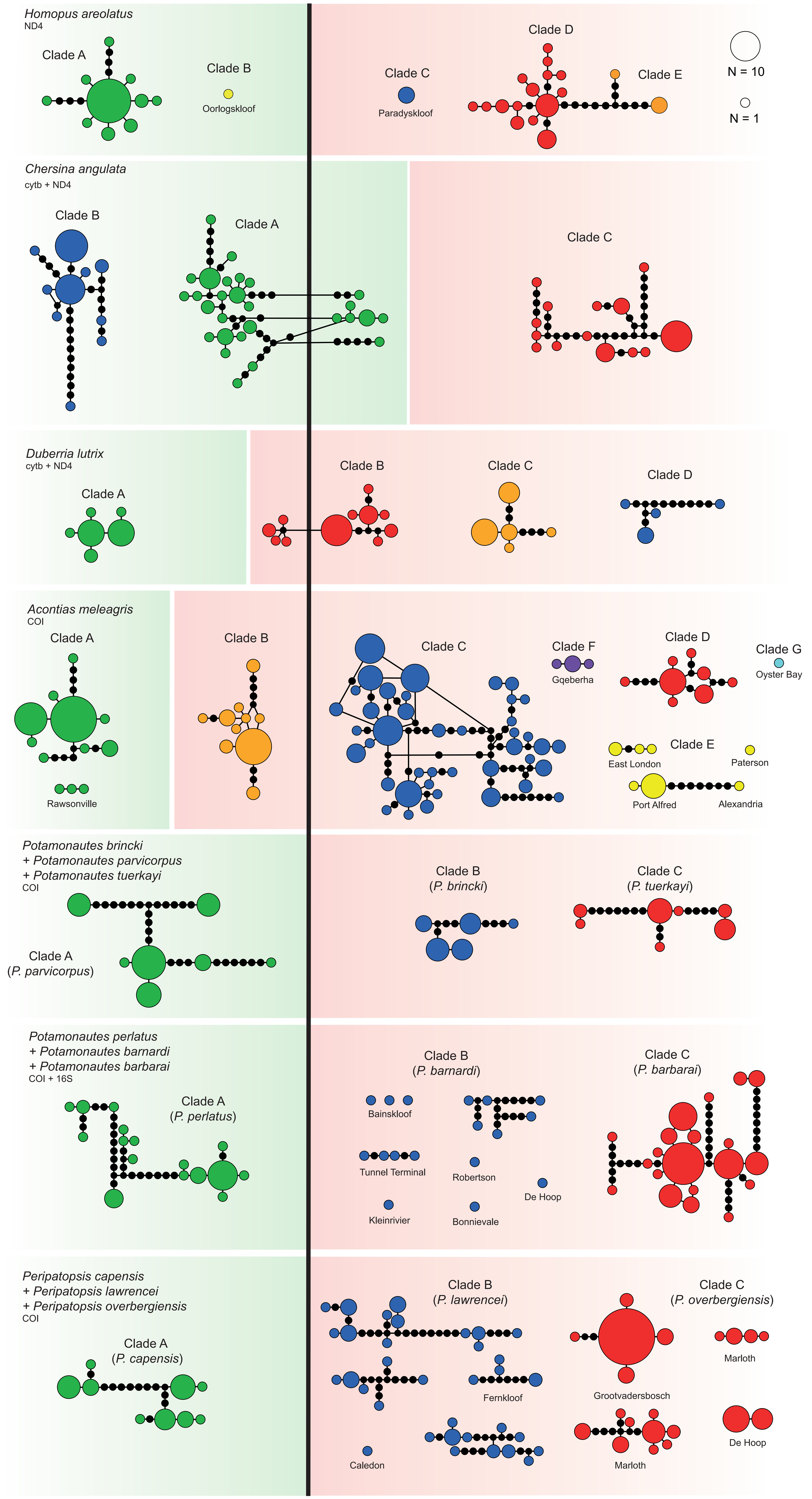
Homopus areolatus
ND4
Clade A
Clade B
Oorlogskloof
Clade C
Paradyskloof
Clade D
Clade E
N = 10
N = 1
Chersina angulata
cytb + ND4
Clade B
Clade A
Clade C
Duberria lutrix
cytb + ND4
Clade A
Clade B
Clade C
Clade D
Acontias meleagris
COI
Clade A
Rawsonville
Clade B
Clade C
Clade F
Gqeberha
Clade D
Clade G
Oyster Bay
Clade E
East London
Paterson
Port Alfred
Alexandria
Potamonautes brincki
+ Potamonautes parvicorpus
+ Potamonautes tuerkayi
COI
Clade A
(P. parvicorpus)
Clade B
(P. brincki)
Clade C
(P. tuerkayi)
Potamonautes perlatus
+ Potamonautes barnardi
+ Potamonautes barbarai
COI + 16S
Clade A
(P. perlatus)
Clade B
(P. barnardi)
Bainskloof
Tunnel Terminal
Robertson
De Hoop
Kleinrivier
Bonnievale
Clade C
(P. barbarai)
Peripatopsis capensis
+ Peripatopsis lawrencei
+ Peripatopsis overbergiensis
COI
Clade A
(P. capensis)
Clade B
(P. lawrencei)
Fernkloof
Caledon
Clade C
(P. overbergiensis)
Grootvadersbosch
Marloth
De Hoop
Marloth
