# Supplementary figures and images for: Between the Cape Fold Mountains and the deep blue sea: Comparative phylogeography of selected codistributed ectotherms reveals asynchronous cladogenesis
Source: Evol Appl. 2022 Oct 27;15(12):1967–87. doi: 10.1111/eva.13493 (PMC9753840; doi:10.1111/eva.13493)

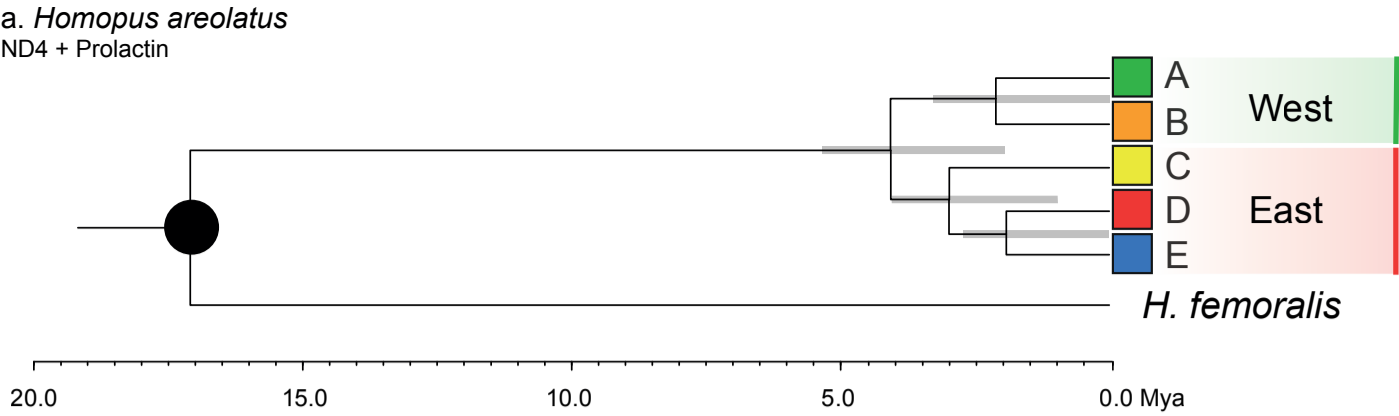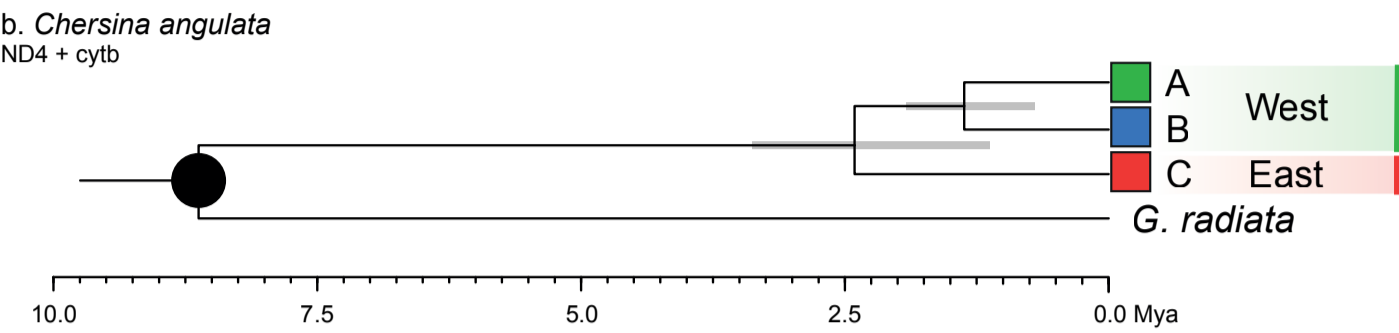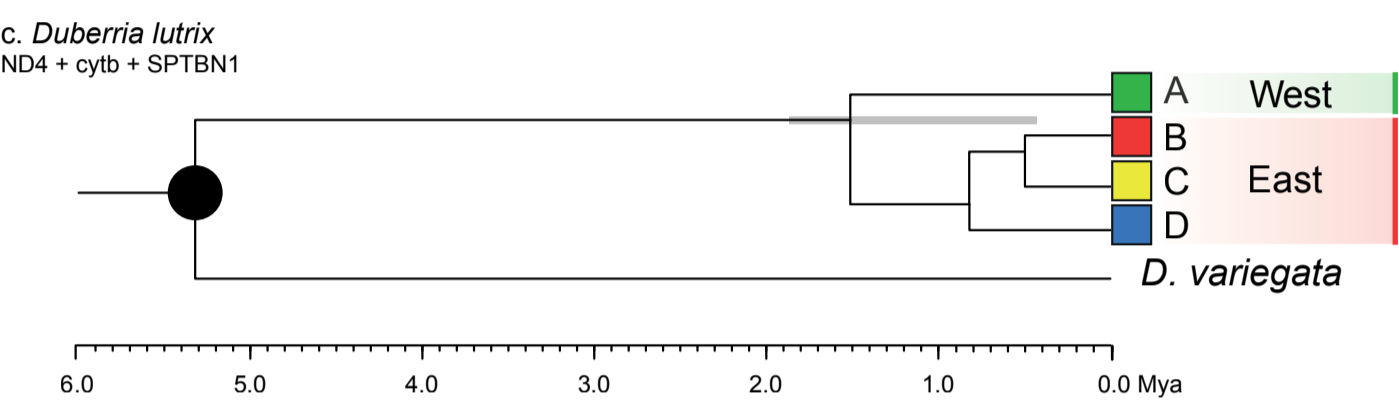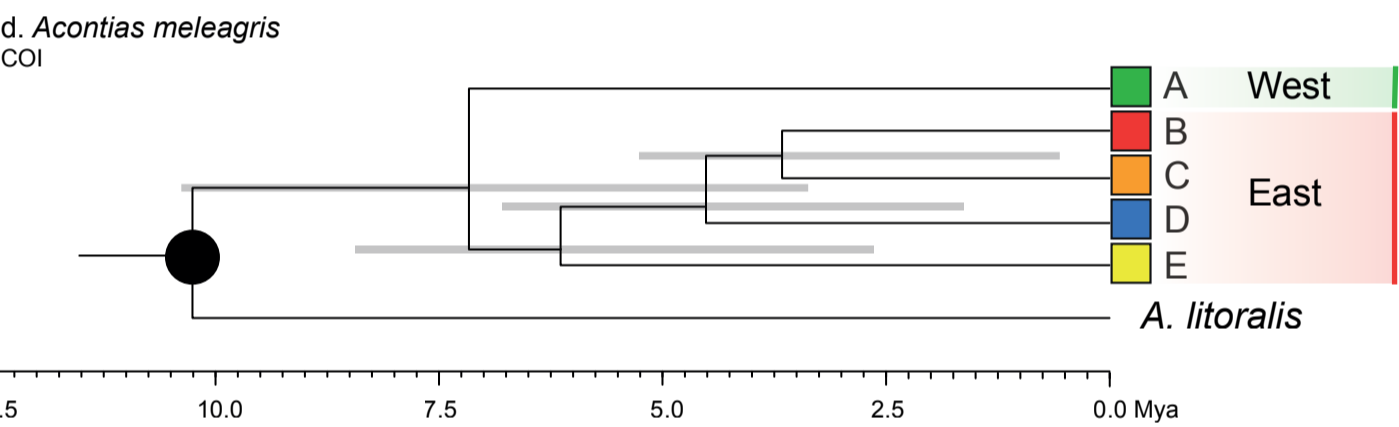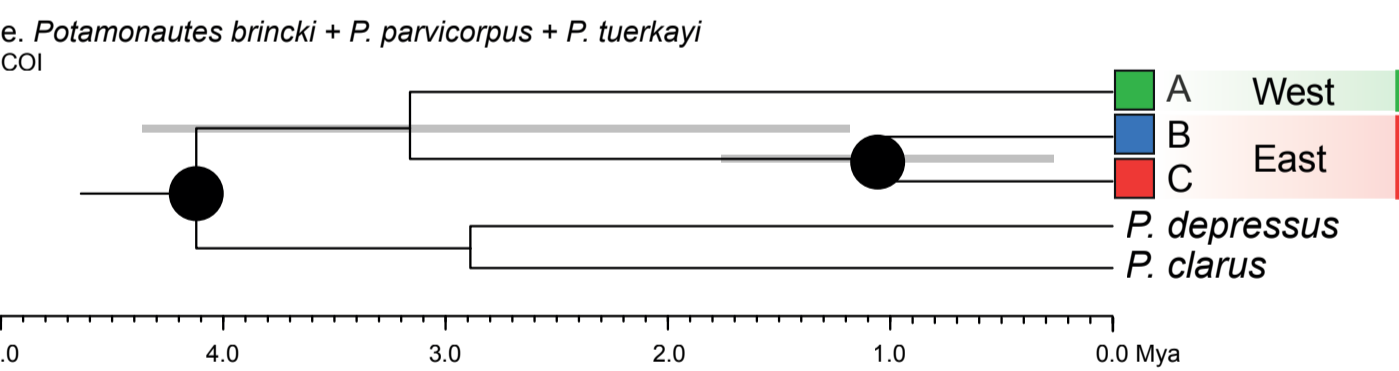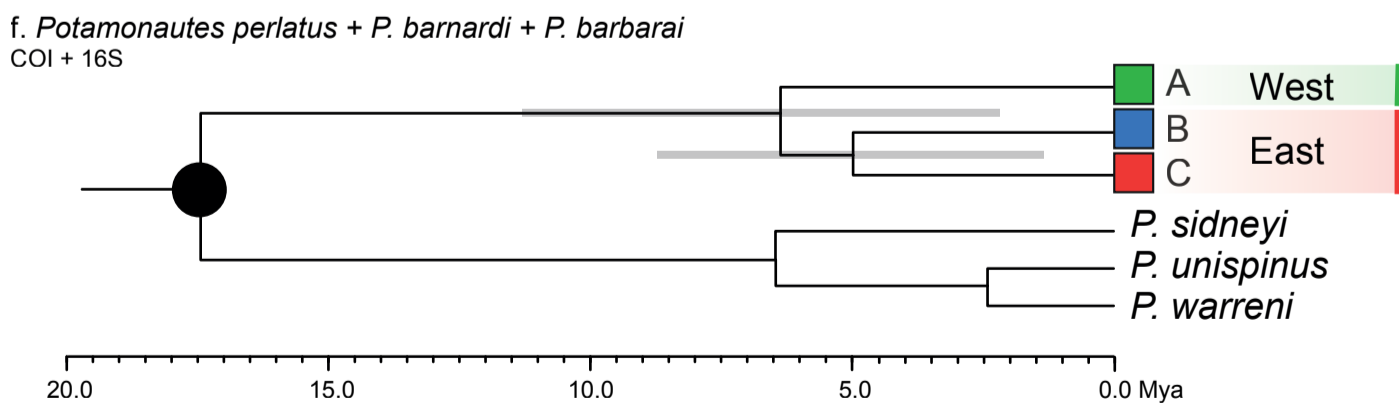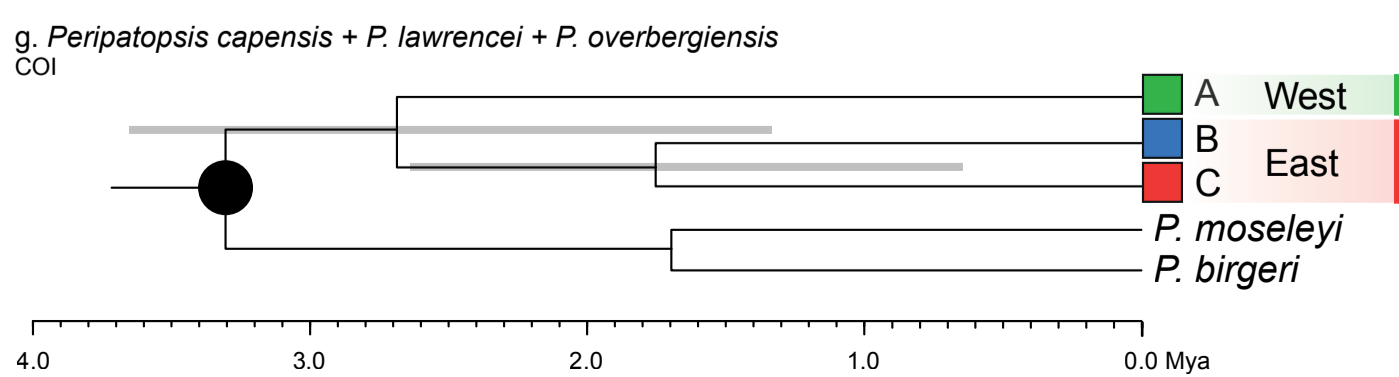

Supplement: Supplementary file 3 — Figure S3 [file EVA-15-1967-s005.pdf]

a. *H. areolatus*

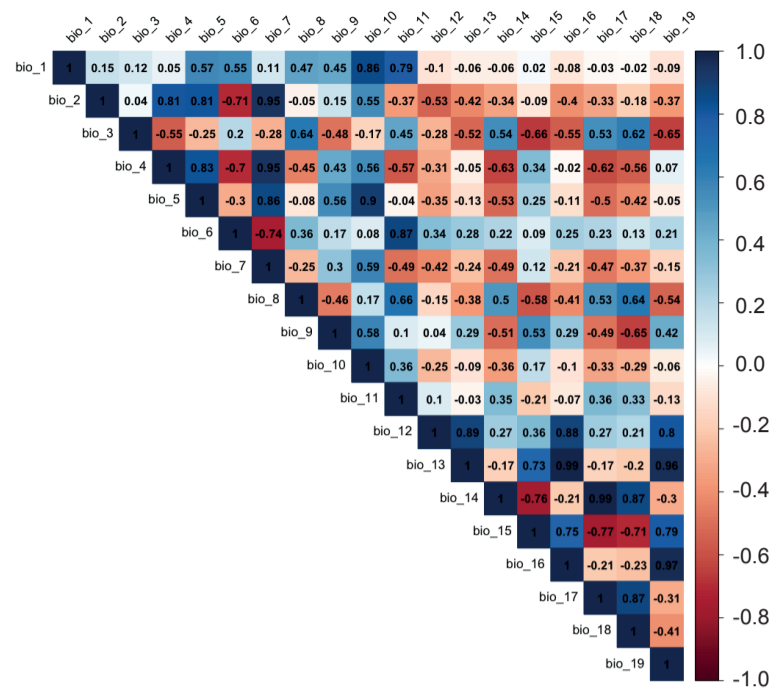

b. *C. angulata*

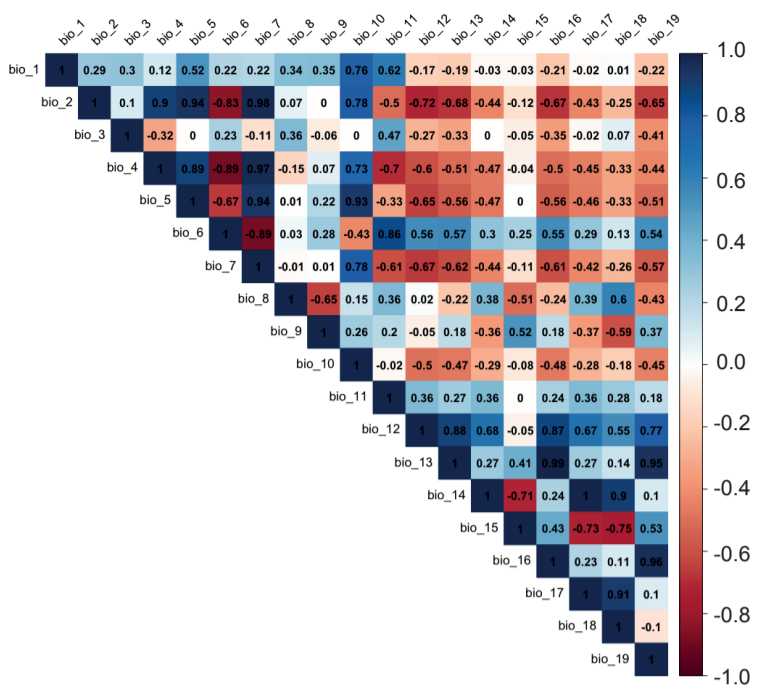

c. *D. lutrix*

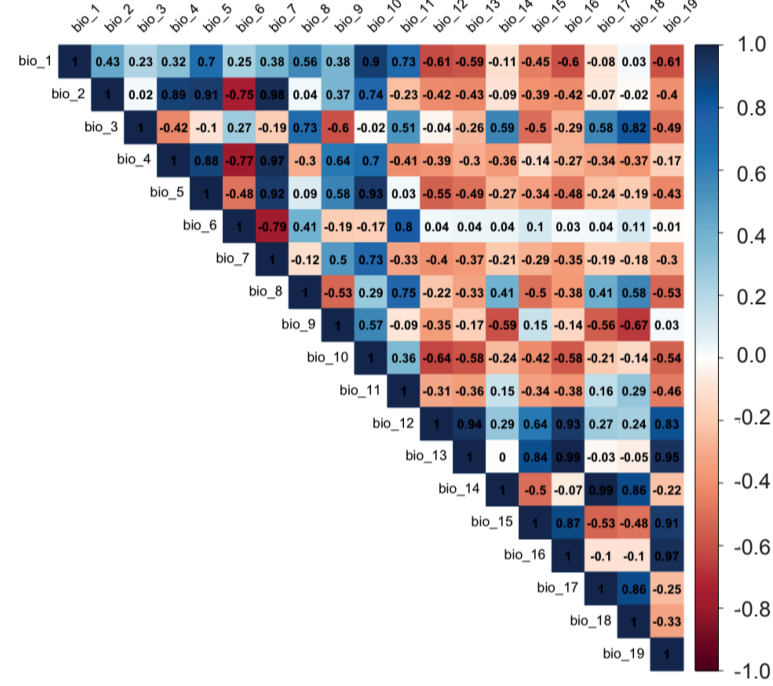

d. *A. meleagris*

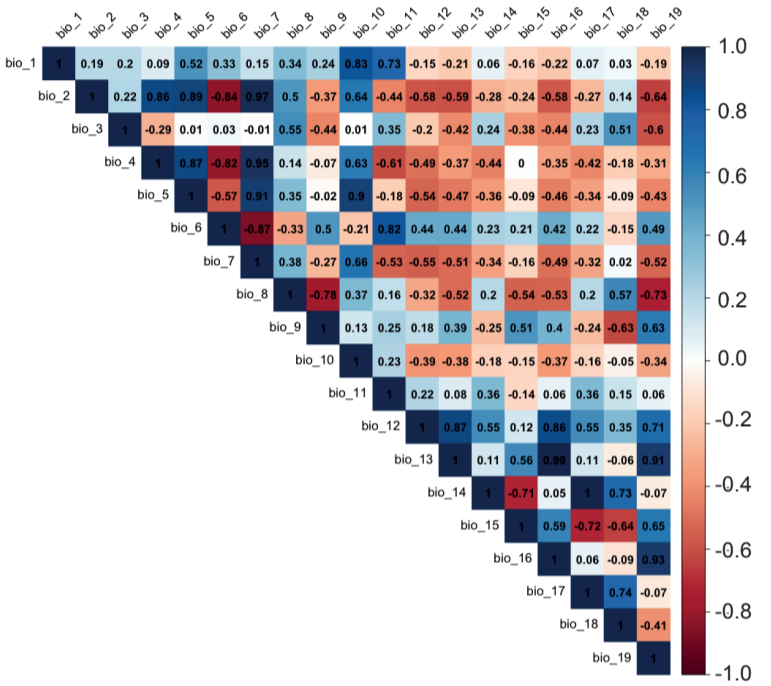

e. *P. brincki* + *P. parvicarpus* + *P. tuerkayi*

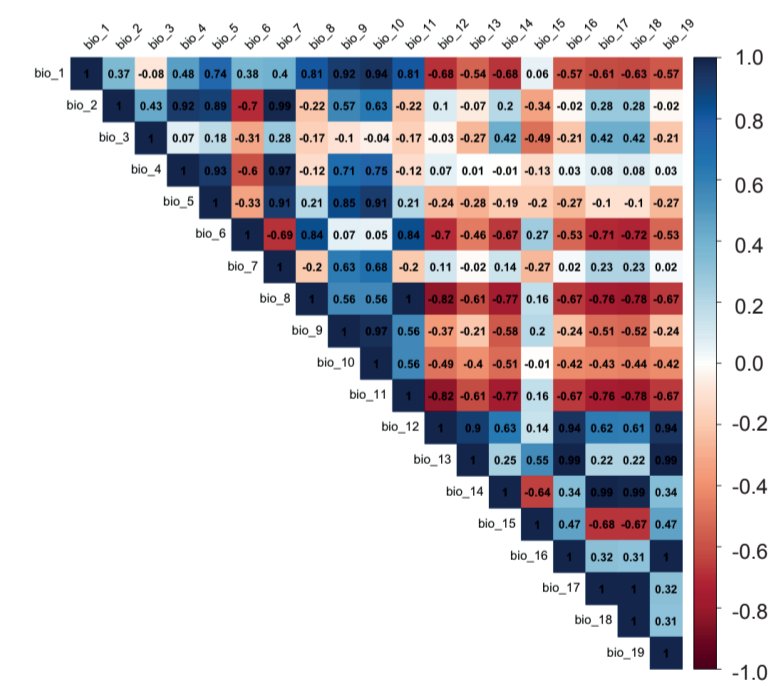

Supplement: Supplementary file 4 — Figure S4 [file EVA-15-1967-s006.pdf]

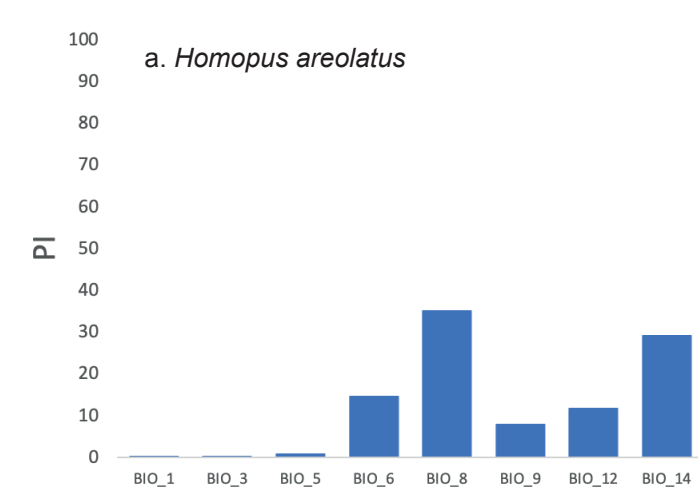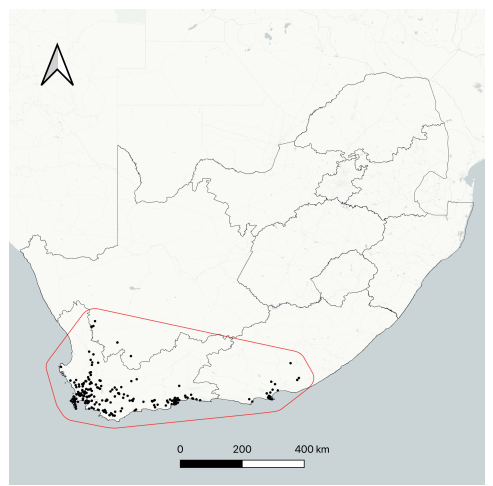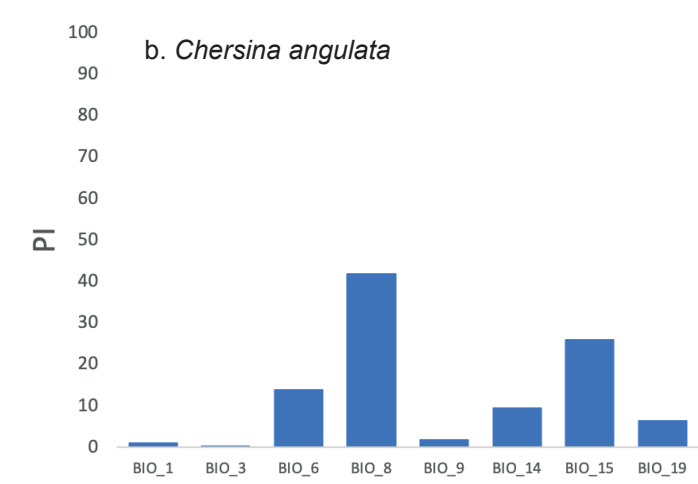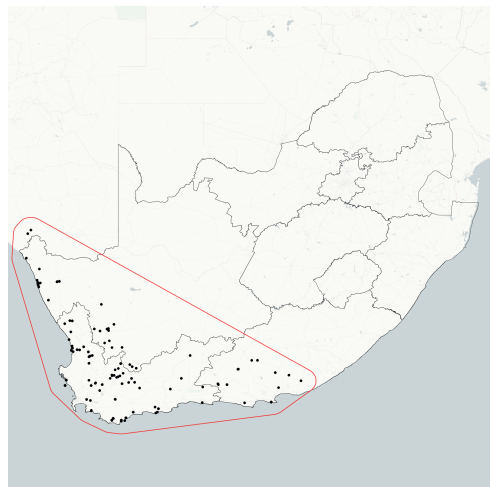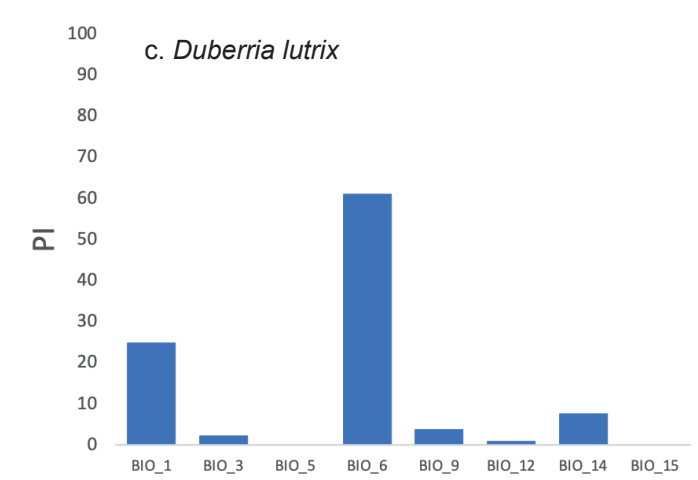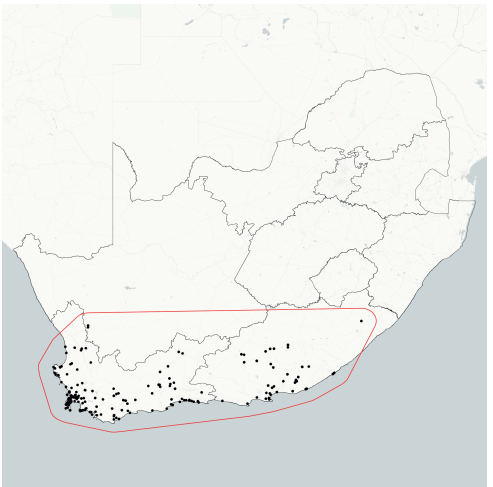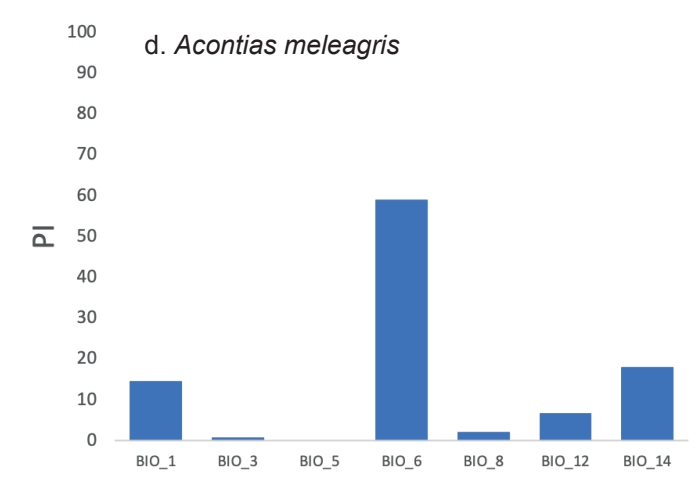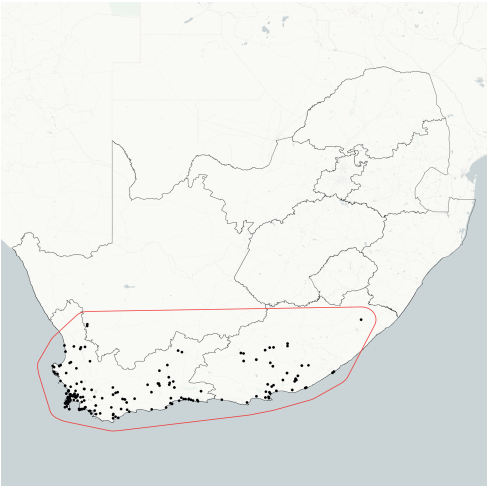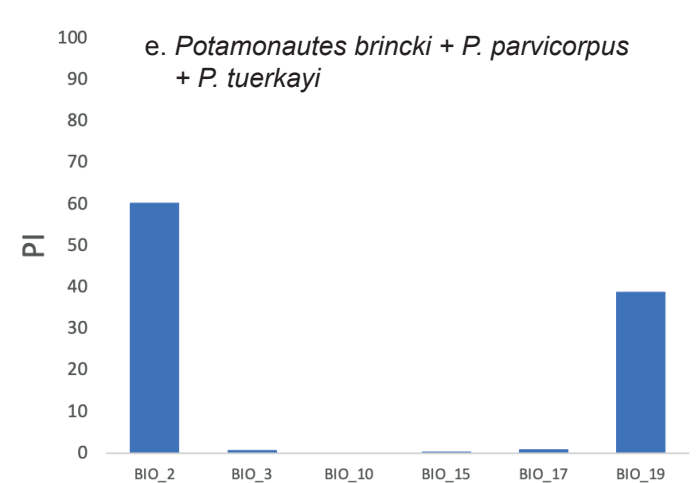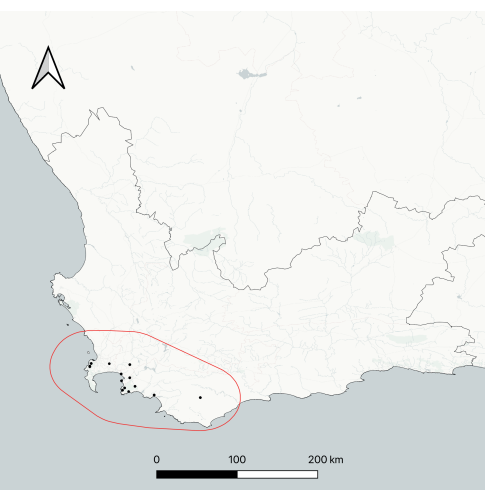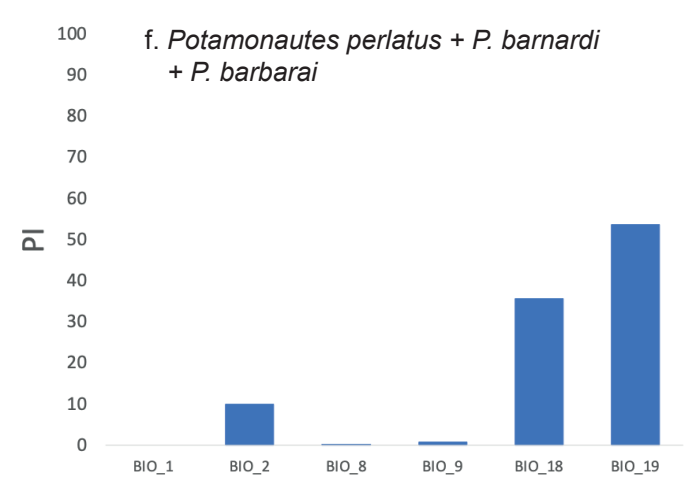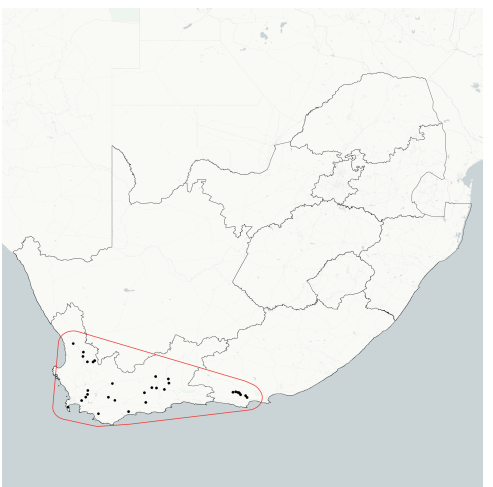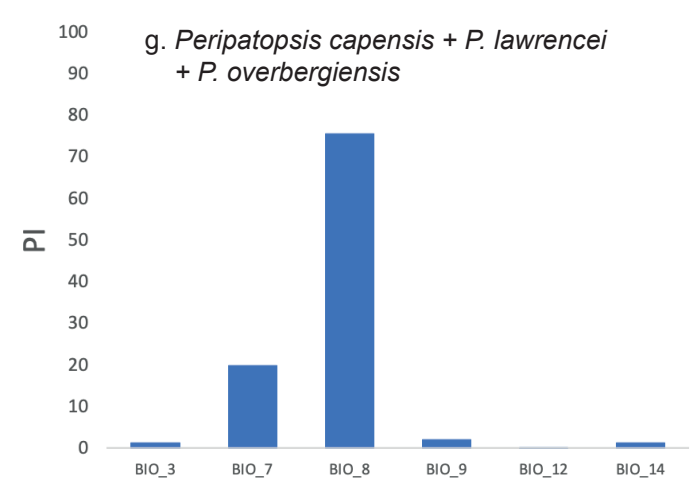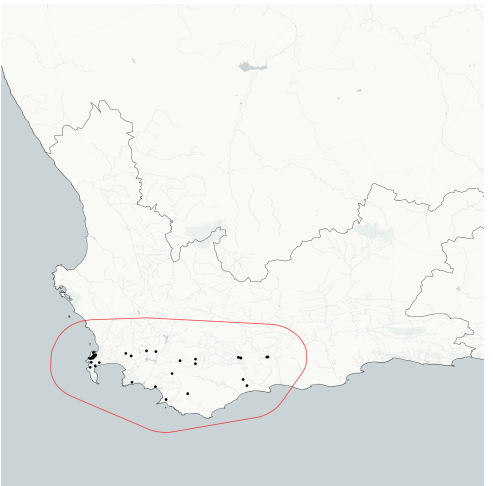

Supplement: Supplementary file 5 — Figure S5 [file EVA-15-1967-s008.pdf]

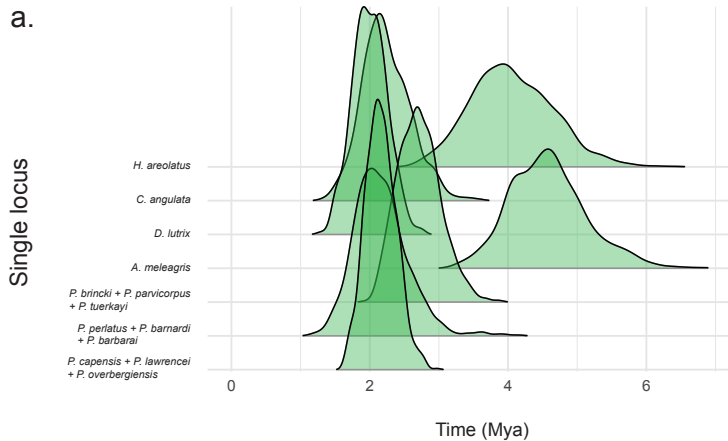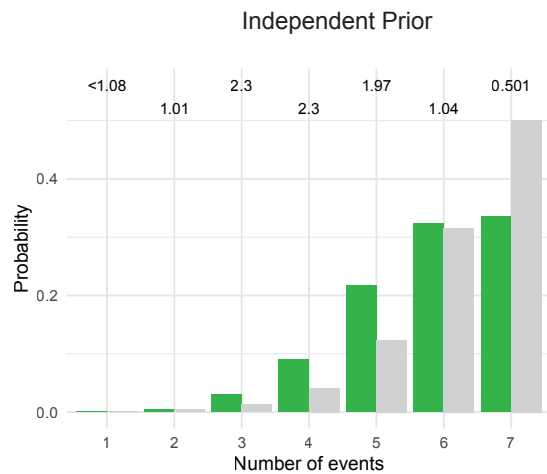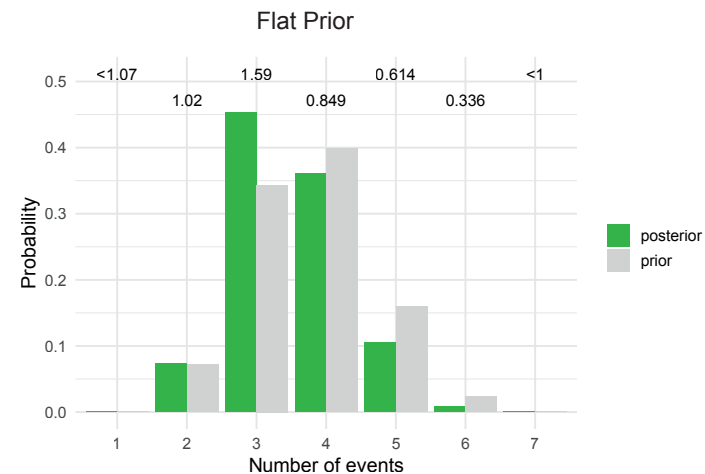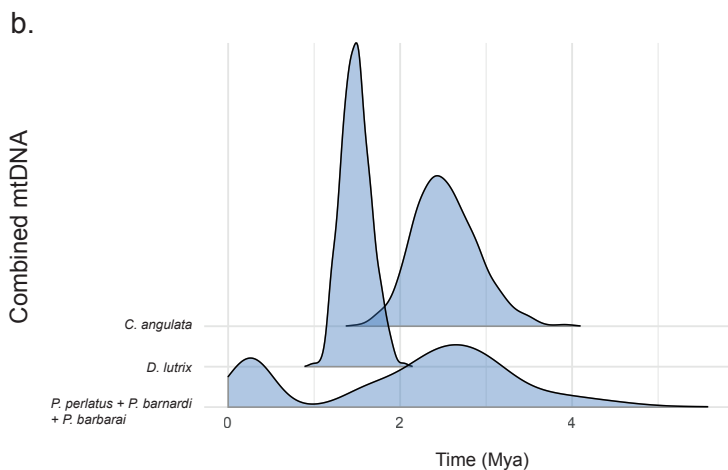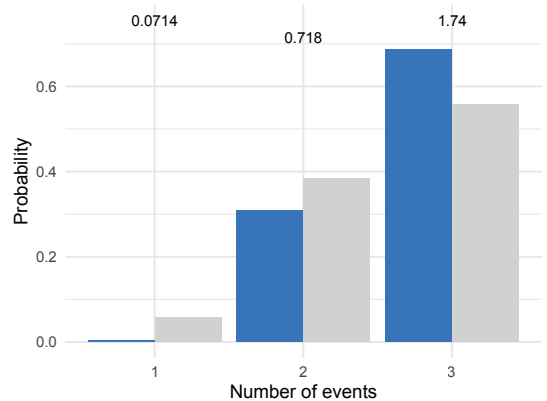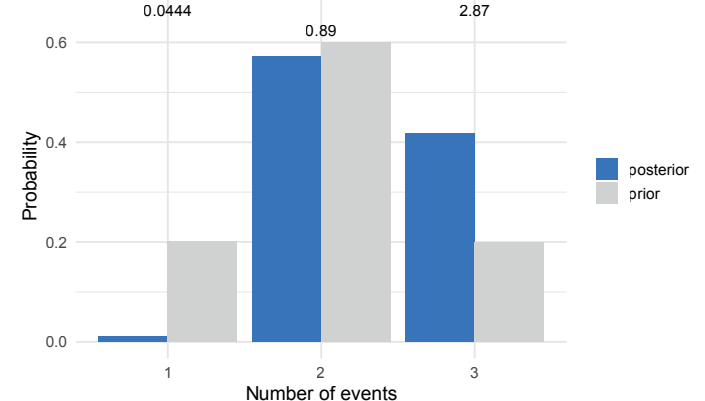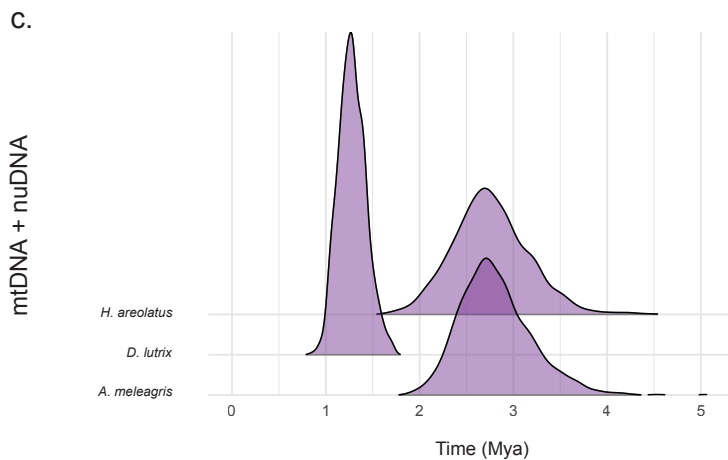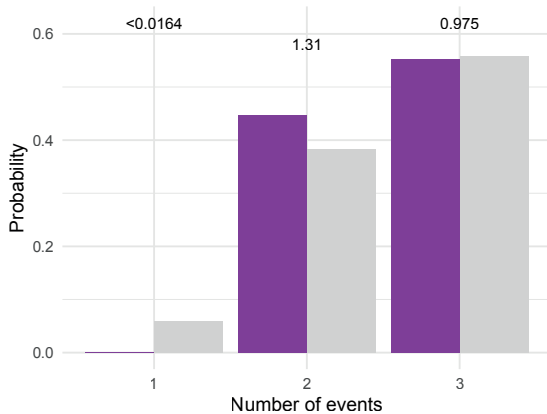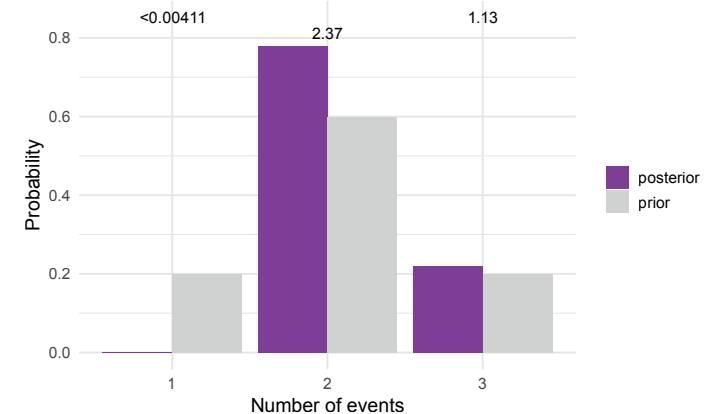

Supplement: Supplementary file 7 — Figure S7 [file EVA-15-1967-s007.pdf]
