# Supplementary material for: Between the Cape Fold Mountains and the deep blue sea: Comparative phylogeography of selected codistributed ectotherms reveals asynchronous cladogenesis
Source: Evol Appl. 2022 Oct 27;15(12):1967–87. doi: 10.1111/eva.13493 (PMC9753840; doi:10.1111/eva.13493)
Supplement: Supplementary file 6 — Figure S6 [file EVA-15-1967-s002.pdf]

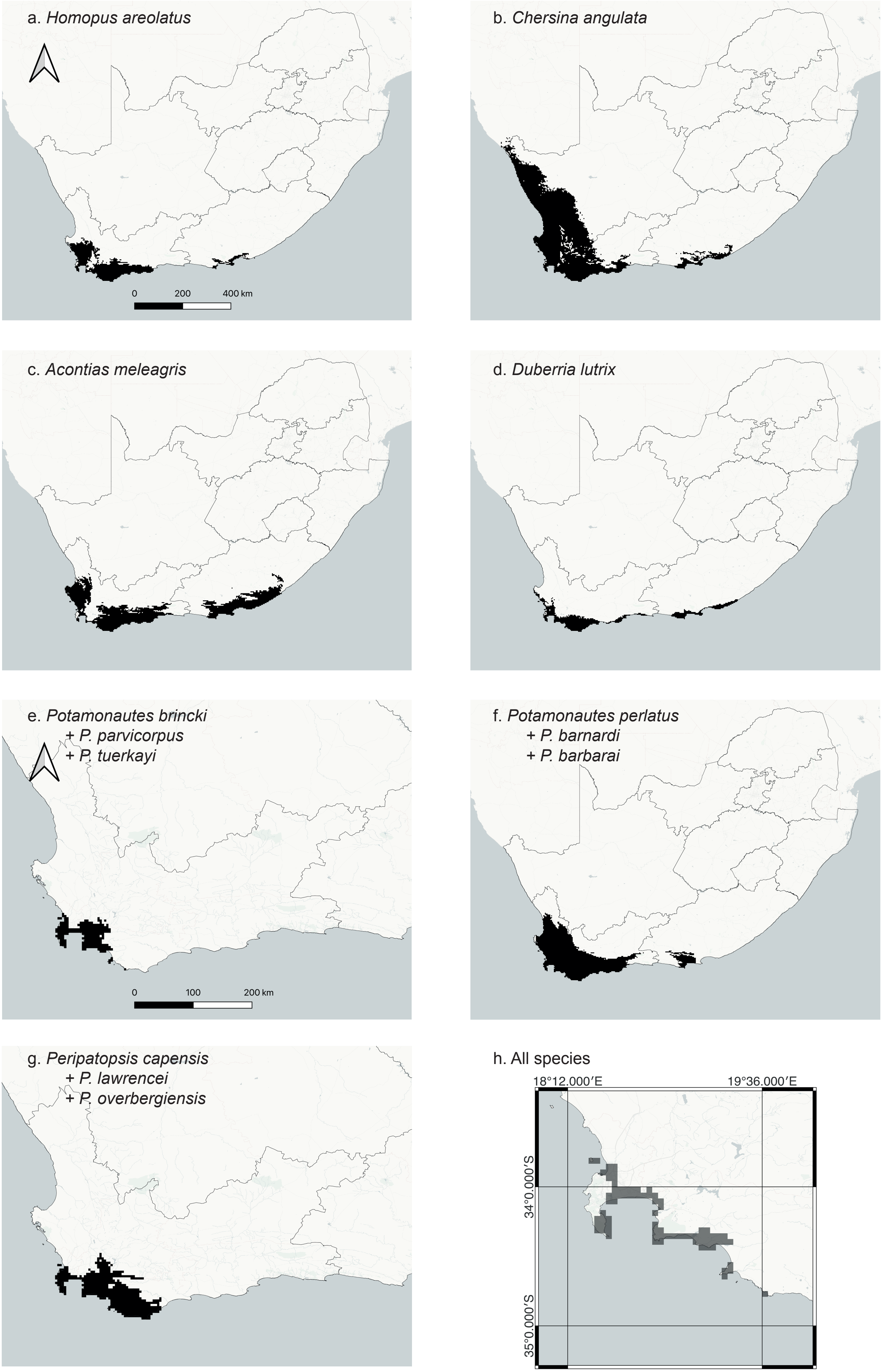
a. Homopus areolatus
0
200
400 km
b. Chersina angulata
c. Acontias meleagris
d. Duberria lutrix
e. Potamonautes brincki
+ P. parvicorpus
+ P. tuerkayi
0
100
200 km
f. Potamonautes perlatus
+ P. barnardi
+ P. barbarai
g. Peripatopsis capensis
+ P. lawrencei
+ P. overbergiensis
h. All species
18°12.000′E
19°36.000′E
34°0.000′S
35°0.000′S
