# Supplementary material for: Between the Cape Fold Mountains and the deep blue sea: Comparative phylogeography of selected codistributed ectotherms reveals asynchronous cladogenesis
Source: Evol Appl. 2022 Oct 27;15(12):1967–87. doi: 10.1111/eva.13493 (PMC9753840; doi:10.1111/eva.13493)
Supplement: Supplementary file 8 — Appendix S1 [file EVA-15-1967-s009.docx]

**Appendix 1 – Supplementary Methods**

**S1.1 | Mitochondrial mutation rates**

Both *H. areolatus* and *C. angulata* belong to the family Testudinidae, and therefore the same mutation rates were used for the ND4 locus sequences from each species, with a mean of 0.4% per lineage per Ma, as well as lower and upper bounds of 0.33% and 0.6%, respectively (Bowen *et al.,* 1993; Caccone *et al.,* 1999; Hofmeyer *et al.*, 2020). The *C. angulata* cytb locus, meanwhile, was also estimated with an initial mean of 0.4%, but with lower and upper bounds of 0.2% and 0.6%, respectively (Bowen *et al.,* 1993; Caccone *et al.,* 1999; Lesia *et al.,* 2003). The *D. lutrix* phylogeny was dated using both ND4 and cytb mitochondrial sequences, which were both estimated with a mean mutation rate of 1.34% per lineage per Ma (Daza *et al.,* 2009; Myers *et al.,* 2017; Kulenkampff *et al.*, 2019; Busschau *et al*., 2022). The legless skink *A. meleagris* samples included the COI locus (with a mean of 0.65%, as well as upper and lower bounds of 0.61% and 0.7%, respectively) (Macey *et al.,* 1998; Weisrock *et al.,* 2001; Honda *et al.,* 2006). The six *Potamonautes* species were investigated using the COI locus (with a mean mutation rate of 2% per Ma, and minimum and maximum values of 1.40% to 2.60%, respectively) (Knowlton & Weight, 1998; Schubart *et al*., 1998; Projecto-Garcia *et al*., 2010; Daniels 2011, Daniels *et al*., 2015; Daniels & Klaus, 2018), while *P. perlatus, P. barnardi* and *P. barbarai* also included the 16S rRNA locus (with a mean mutation rate of 1.02% per Ma, and lower and upper bounds of 0.64% and 1.42%) (Schubart *et al.,* 1998; Klaus *et al.,* 2010). Finally, the combined phylogeny of the velvet worm species *Per. capensis, Per. lawrencei* and *Per. overbergiensis* was estimated using the COI locus with a mean mutation rate of 1.9% per Ma, with lower and upper bounds of 1.5% and 2.3%, respectively (Brower, 1994; Farrell, 2001; Trewick & Wallis, 2001; Boyer *et al.,* 2007; Daniels, 2011; McDonald & Daniels, 2012; Myburgh & Daniels, 2015). All of the above phylogenies were estimated using a relaxed clock with a log-normal distribution (Drummond *et al.,* 2006).

**S1.2 | Simultaneous divergence**

*BEAST analyses were run for 100 million generation, sampling every 10000 generations. Tracer was used to assess convergence (ESS >200), as well as to identify the mean and 95% HPD divergence time estimates per locus after 20% of the posterior distribution had been discarded as burn-in.

As Ecoevolity is not able to specify separate mutation rates when using multiple loci per species, we determined weighted mean mutation rates for each combination of loci per taxon (Table S3.20). These weighted mutation rates were calculated using the proportional contribution of each locus to the overall concatenated sequence length. Since Ecoevolity assumes that each character is biallelic, nucleotides were recoded as either being in an initial state (0) or a different state (1). Oaks (2019) suggested an alternative approach in which sites with more than two states are removed, although noted that they had not found any discernable differences in the results between these two methods. Furthermore, in the mtDNA loci studied, only three taxa featured a rate of triallelic sites of over 1%, specifically *P. brincki + P. parvicorpus + P. tuerkayi* (1.30%), *P. capensis + P. lawrencei + P. overbergiensis* (1.88%) and *A. meleagris* (3.62%).

Two sets of analyses were run, using a more conservative ‘independent’ Dirichlet-process prior (which places a prior probability of 50% on the maximum number of events), as well as a ‘flat’ Uniform prior (which places equal probability on each possible model). The Dirichlet concentration prior used in the Ecoevolity analyses was determined using the program DPPROPS. A gamma distribution was placed on the event time prior, using the mean divergence time determined by the *BEAST analyses (shape = 2.0, mean = 3.49). The operator settings were set to auto optimization, with a delay value of 1000. The global mutation rate was set to 1.0, and individual mutation rates were specified and scaled for each taxon resulting in posterior divergence-times in million years. Population size priors were specified for each taxon, with effective population sizes determined using theta values retrieved from DnaSP 6.12 (Rozas *et al.,* 2017). Ten independent MCMC chains were run for 10000 generations, sampling every 100 generations. Convergence was assessed using the pyco-sumchains function in Ecoevolity and visualised using Tracer (ESS >200).
