# Supplementary material for: Between the Cape Fold Mountains and the deep blue sea: Comparative phylogeography of selected codistributed ectotherms reveals asynchronous cladogenesis
Source: Evol Appl. 2022 Oct 27;15(12):1967–87. doi: 10.1111/eva.13493 (PMC9753840; doi:10.1111/eva.13493)
Supplement: Supplementary file 9 — Appendix S2 [file EVA-15-1967-s003.docx]

**Table S2.** List of samples included in the study. Outgroups are indicated with asterisks (*)

| Taxon | Voucher ID | Sample ID | Locality | Latitude | Longitude | ND4 | Cytb | 16S | COI | PRLR | EXPH5 |
| --- | --- | --- | --- | --- | --- | --- | --- | --- | --- | --- | --- |
| *Homopus areolatus* |  |  |  |  |  |  |  |  |  |  |  |
| *Homopus areolatus* |  | Blomfontein1 | Blomfontein farm, WC | -31.9527 | 19.9825 | MN245572 |  |  |  |  |  |
| *Homopus areolatus* |  | Blomfontein2 | Blomfontein farm, WC | -31.9527 | 19.9825 | MN245573 |  |  |  |  |  |
| *Homopus areolatus* |  | Blomfontein3 | Blomfontein farm, WC | -31.9527 | 19.9825 | MN245574 |  |  |  |  |  |
| *Homopus areolatus* |  | Malmesbury1 | Malmesbury, WC | -33.4968 | 18.727 | MN245575 |  |  |  | MN245646 |  |
| *Homopus areolatus* |  | ParadysEC1 | Paradys, EC | -34.1014 | 24.8896 | MN245576 |  |  |  |  |  |
| *Homopus areolatus* |  | Oorlogskloof1 | Oorlogskloof Reservce, WC | -31.3954 | 19.0437 | MN245577 |  |  |  | MN245649 |  |
| *Homopus areolatus* |  | Adelaide | Adlaide, EC | -32.7039 | 26.2821 | MN245578 |  |  |  | MN245653 |  |
| *Homopus areolatus* |  | Franschoek1 | Franschoek, WC | -33.9185 | 19.1386 | MN245581 |  |  |  | MN245654 |  |
| *Homopus areolatus* |  | Franschoek2 | Franschoek, WC | -33.9185 | 19.1386 | MN245580 |  |  |  |  |  |
| *Homopus areolatus* |  | Franschoek3 | Franschoek, WC | -33.9185 | 19.1386 | MN245579 |  |  |  |  |  |
| *Homopus areolatus* |  | Stillbay1 | Still Bay, WC | -34.2339 | 21.3212 | MN245583 |  |  |  | MN245650 |  |
| *Homopus areolatus* |  | Stillbay2 | Still Bay, WC | -34.2339 | 21.3212 | MN245582 |  |  |  |  |  |
| *Homopus areolatus* |  | RiverlandS2 | Riverlands Reserve, WC | -33.4791 | 18.5816 | MN245584 |  |  |  |  |  |
| *Homopus areolatus* |  | Langfontein1 | Langfontein farm, WC | -33.3457 | 19.3699 | MN245588 |  |  |  |  |  |
| *Homopus areolatus* |  | Langfontein2 | Langfontein farm, WC | -33.3457 | 19.3699 | MN245587 |  |  |  |  |  |
| *Homopus areolatus* |  | Langfontein3 | Langfontein farm, WC | -33.3457 | 19.3699 | MN245586 |  |  |  |  |  |
| *Homopus areolatus* |  | Langfontein5 | Langfontein farm, WC | -33.3457 | 19.3699 | MN245585 |  |  |  |  |  |
| *Homopus areolatus* |  | Swartwalle1 | Swartwalle farm, WC | -33.5986 | 19.2832 | MN245591 |  |  |  | MN245651 |  |
| *Homopus areolatus* |  | Swartwalle2 | Swartwalle farm, WC | -33.5986 | 19.2832 | MN245590 |  |  |  |  |  |
| *Homopus areolatus* |  | Swartwalle3 | Swartwalle farm, WC | -33.5986 | 19.2832 | MN245589 |  |  |  |  |  |
| *Homopus areolatus* |  | RomansRiver1 | Romans River, WC | -33.4838 | 19.2028 | MN245594 |  |  |  | KX245655 |  |
| *Homopus areolatus* |  | RomansRiver2 | Romans River, WC | -33.4838 | 19.2028 | MN245593 |  |  |  |  |  |
| *Homopus areolatus* |  | RomansRiver3 | Romans River, WC | -33.4838 | 19.2028 | MN245592 |  |  |  |  |  |
| *Homopus areolatus* |  | Perdefontein | Perdefontein farm, WC | -33.3291 | 19.3346 | MN245595 |  |  |  | MN245643 |  |
| *Homopus areolatus* |  | Chelance1 | Chelance farm, WC | -33.6342 | 19.3397 | MN245596 |  |  |  |  |  |
| *Homopus areolatus* |  | PEM17230 | Gqeberha, EC | -34.018 | 25.6088 | MN245597 |  |  |  |  |  |
| *Homopus areolatus* |  | MB20984 | Herbertsdale, WC | -34.0658 | 21.7925 | MN245598 |  |  |  |  |  |
| *Homopus areolatus* |  | Buffelsjag1 | Buffeljags River, WC | -34.075 | 20.54 | MN245604 |  |  |  | MN245640 |  |
| *Homopus areolatus* |  | Buffelsjag2 | Buffeljags River, WC | -34.075 | 20.54 | MN245603 |  |  |  |  |  |
| *Homopus areolatus* |  | Buffelsjag3 | Buffeljags River, WC | -34.075 | 20.54 | MN245602 |  |  |  |  |  |
| *Homopus areolatus* |  | Buffelsjag4 | Buffeljags River, WC | -34.075 | 20.54 | MN245601 |  |  |  |  |  |
| *Homopus areolatus* |  | Buffelsjag5 | Buffeljags River, WC | -34.075 | 20.54 | MN245600 |  |  |  |  |  |
| *Homopus areolatus* |  | Buffelsjag6 | Buffeljags River, WC | -34.075 | 20.54 | MN245599 |  |  |  |  |  |
| *Homopus areolatus* |  | Clanwilliam | Clanwilliam, WC | -32.285 | 18.955 | MN245605 |  |  |  |  |  |
| *Homopus areolatus* |  | EPNR41 | Elandsberg NR, WC | -33.4264 | 19.0319 | MN245606 |  |  |  |  |  |
| *Homopus areolatus* |  | EPNR42 | Elandsberg NR, WC | -33.4264 | 19.0319 | MN245607 |  |  |  |  |  |
| *Homopus areolatus* |  | EPNR44 | Elandsberg NR, WC | -33.4264 | 19.0319 | MN245608 |  |  |  |  |  |
| *Homopus areolatus* |  | EPNR46 | Elandsberg NR, WC | -33.4264 | 19.0319 | MN245609 |  |  |  |  |  |
| *Homopus areolatus* |  | EPNR47 | Elandsberg NR, WC | -33.4264 | 19.0319 | MN245610 |  |  |  |  |  |
| *Homopus areolatus* |  | EPNR48 | Elandsberg NR, WC | -33.4264 | 19.0319 | MN245611 |  |  |  |  |  |
| *Homopus areolatus* |  | EPNR51 | Elandsberg NR, WC | -33.4264 | 19.0319 | MN245612 |  |  |  |  |  |
| *Homopus areolatus* |  | EPNR52 | Elandsberg NR, WC | -33.4264 | 19.0319 | MN245613 |  |  |  |  |  |
| *Homopus areolatus* |  | EPNR55 | Elandsberg NR, WC | -33.4264 | 19.0319 | MN245614 |  |  |  |  |  |
| *Homopus areolatus* |  | EPNR56 | Elandsberg NR, WC | -33.4264 | 19.0319 | MN245615 |  |  |  |  |  |
| *Homopus areolatus* |  | EPNR57 | Elandsberg NR, WC | -33.4264 | 19.0319 | MN245616 |  |  |  |  |  |
| *Homopus areolatus* |  | EPNR58 | Elandsberg NR, WC | -33.4264 | 19.0319 | MN245617 |  |  |  |  |  |
| *Homopus areolatus* |  | EPNR59 | Elandsberg NR, WC | -33.4264 | 19.0319 | MN245618 |  |  |  |  |  |
| *Homopus areolatus* |  | EPNR60 | Elandsberg NR, WC | -33.4264 | 19.0319 | MN245619 |  |  |  |  |  |
| *Homopus areolatus* |  | HH1 | Hottentots Holland Reserve, WC | -34.0707 | 19.0498 | MN245620 |  |  |  | MN245648 |  |
| *Homopus areolatus* |  | HH2 | Hottentots Holland Reserve, WC | -34.0707 | 19.0498 | MN245621 |  |  |  |  |  |
| *Homopus areolatus* |  | HH3 | Hottentots Holland Reserve, WC | -34.0707 | 19.0498 | MN245622 |  |  |  |  |  |
| *Homopus areolatus* |  | HH4 | Hottentots Holland Reserve, WC | -34.0707 | 19.0498 | MN245623 |  |  |  |  |  |
| *Homopus areolatus* |  | HH5 | Hottentots Holland Reserve, WC | -34.0707 | 19.0498 | MN245624 |  |  |  |  |  |
| *Homopus areolatus* |  | HH6 | Hottentots Holland Reserve, WC | -34.0707 | 19.0498 | MN245625 |  |  |  | MN245644 |  |
| *Homopus areolatus* |  | HH7 | Hottentots Holland Reserve, WC | -34.0707 | 19.0498 | MN245626 |  |  |  |  |  |
| *Homopus areolatus* |  | HH8 | Hottentots Holland Reserve, WC | -34.0707 | 19.0498 | MN245627 |  |  |  |  |  |
| *Homopus areolatus* |  | Kosierskraal4 | Kosierskraal farm, WC | -34.6133 | 19.8833 | MN245628 |  |  |  |  |  |
| *Homopus areolatus* |  | Kosierskraal5 | Kosierskraal farm, WC | -34.6133 | 19.8833 | MN245629 |  |  |  |  |  |
| *Homopus areolatus* |  | Montague | Montague, WC | -33.892 | 20.355 | MN245630 |  |  |  | MN245641 |  |
| *Homopus areolatus* |  | Rooivlei | Rooivlei farm, WC | -34.35 | 20.15 | MN245631 |  |  |  |  |  |
| *Homopus areolatus* |  | Idasvalley | Idasvalley, WC | -33.9242 | 18.8887 | MN245632 |  |  |  | KX346388 |  |
| *Homopus areolatus* |  | Moddervlei | Moddervlei, WC | -34.6073 | 19.8231 | MN245633 |  |  |  |  |  |
| *Homopus areolatus* |  | SomersetWest1 | Somerset West, WC | -34.0625 | 18.87 | MN245634 |  |  |  |  |  |
| *Homopus areolatus* |  | SomersetWest2 | Somerset West, WC | -34.0625 | 18.87 | MN245635 |  |  |  |  |  |
| *Homopus areolatus* |  | Vrolikheid1 | Vrolikheid district, WC | -33.9141 | 19.8907 | MN245636 |  |  |  | MN245645 |  |
| *Homopus areolatus* |  | Vrolikheid2 | Vrolikheid district, WC | -33.9141 | 19.8907 | MN245637 |  |  |  |  |  |
| *Homopus areolatus* |  | Vrolikheid3 | Vrolikheid district, WC | -33.9141 | 19.8907 | MN245638 |  |  |  |  |  |
| *Homopus areolatus* |  | Vrolikheid4 | Vrolikheid district, WC | -33.9141 | 19.8907 | MN245639 |  |  |  |  |  |
| *Homopus femoralis** |  | Loxton1HF | Loxton district, NC | -32.0627 | 22.4484 | KX346346 |  |  |  |  |  |
| *Homopus femoralis** |  | BenfonteinHf | Benfontein farm, FS | -28.8887 | 24.8354 | KX346347 |  |  |  | KX346383 |  |
| *Chersina angulata* |  |  |  |  |  |  |  |  |  |  |  |
| *Chersina angulata* |  | Klipdrift1 | Klipdrift farm, WC | -33.23855721 | 18.94725378 | EF120554 | EF120526 |  |  |  |  |
| *Chersina angulata* |  | Kleinmond1 | Kleinmond, WC | -34.31666667 | 19.03333333 | EF120574 | EF120522 |  |  |  |  |
| *Chersina angulata* |  | Kleinmond2 | Kleinmond, WC | -34.31666667 | 19.03333333 | EF120557 | EF120539 |  |  |  |  |
| *Chersina angulata* |  | Kleinmond3 | Kleinmond, WC | -34.31666667 | 19.03333333 | EF120557 | EF120539 |  |  |  |  |
| *Chersina angulata* |  | Algeria | Algeria, WC | -32.35 | 19.06666667 | EF120565 | EF120516 |  |  |  |  |
| *Chersina angulata* |  | Graafwater | Graafwater, WC | -32.15 | 18.6125 | EF120565 | EF120516 |  |  |  |  |
| *Chersina angulata* |  | Vlooikraal1 | Vlooikraal, WC | -34.66666667 | 19.76666667 | EF120561 | EF120517 |  |  |  |  |
| *Chersina angulata* |  | Vlooikraal2 | Vlooikraal, WC | -34.66666667 | 19.76666667 | EF120570 | EF120530 |  |  |  |  |
| *Chersina angulata* |  | Modder1 | Moddervlei, WC | -34.6 | 19.81666667 | EF120573 | EF120530 |  |  |  |  |
| *Chersina angulata* |  | Modder2 | Moddervlei, WC | -34.6 | 19.81666667 | EF120571 | EF120529 |  |  |  |  |
| *Chersina angulata* |  | Modder3 | Moddervlei, WC | -34.6 | 19.81666667 | EF120572 | EF120530 |  |  |  |  |
| *Chersina angulata* |  | Modder4 | Moddervlei, WC | -34.6 | 19.81666667 | EF120575 | EF120530 |  |  |  |  |
| *Chersina angulata* |  | Vrolik1 | Vrolijkheid, WC | -33.9225 | 19.89111111 | EF120563 | EF120517 |  |  |  |  |
| *Chersina angulata* |  | Vrolik2 | Vrolijkheid, WC | -33.9225 | 19.89111111 | EF120564 | EF120518 |  |  |  |  |
| *Chersina angulata* |  | Vrolik3 | Vrolijkheid, WC | -33.9225 | 19.89111111 | EF120564 | EF120517 |  |  |  |  |
| *Chersina angulata* |  | Vrolik4 | Vrolijkheid, WC | -33.9225 | 19.89111111 | EF120564 | EF120517 |  |  |  |  |
| *Chersina angulata* |  | Vrolik5 | Vrolijkheid, WC | -33.9225 | 19.89111111 | EF120564 | EF120517 |  |  |  |  |
| *Chersina angulata* |  | Vrolik6 | Vrolijkheid, WC | -33.9225 | 19.89111111 | EF120562 | EF120517 |  |  |  |  |
| *Chersina angulata* |  | KZ1F | Kleinsee, NC | -29.79166667 | 17.11777778 | EF120548 | EF120511 |  |  |  |  |
| *Chersina angulata* |  | KZ4F | Kleinsee, NC | -29.79166667 | 17.11777778 | EF120549 | EF120511 |  |  |  |  |
| *Chersina angulata* |  | KZ5F | Kleinsee, NC | -29.79166667 | 17.11777778 | EF120546 | EF120510 |  |  |  |  |
| *Chersina angulata* |  | KZ6F | Kleinsee, NC | -29.79166667 | 17.11777778 | EF120546 | EF120511 |  |  |  |  |
| *Chersina angulata* |  | Citrusdal1 | Citrusdal, WC | -32.39583333 | 18.95055556 | EF120552 | EF120511 |  |  |  |  |
| *Chersina angulata* |  | Kriedouw1 | Kriedouwkrans, WC | -32.37055556 | 18.98805556 | EF120566 | EF120520 |  |  |  |  |
| *Chersina angulata* |  | Kriedouw2 | Kriedouwkrans, WC | -32.37055556 | 18.98805556 | EF120566 | EF120519 |  |  |  |  |
| *Chersina angulata* |  | TK1 | Tierberg, WC | -33.17027778 | 22.26527778 | EF120576 | EF120538 |  |  |  |  |
| *Chersina angulata* |  | WB1 | Witteberg, WC | -33.31111111 | 20.58833333 | EF120553 | EF120531 |  |  |  |  |
| *Chersina angulata* |  | WB2 | Witteberg, WC | -33.31111111 | 20.58833333 | EF120553 |  |  |  |  |  |
| *Chersina angulata* |  | Stilbaai1 | Still Bay, WC | -34.25666667 | 21.44472222 | EF120568 | EF120541 |  |  |  |  |
| *Chersina angulata* |  | Stilbaai2 | Still Bay, WC | -34.25666667 | 21.44472222 | EF120567 | EF120540 |  |  |  |  |
| *Chersina angulata* |  | Stilbaai3 | Still Bay, WC | -34.25666667 | 21.44472222 | EF120569 | EF120540 |  |  |  |  |
| *Chersina angulata* |  | Stilbaai4 | Still Bay, WC | -34.25666667 | 21.44472222 | EF120567 | EF120540 |  |  |  |  |
| *Chersina angulata* |  | Stilbaai5 | Still Bay, WC | -34.25666667 | 21.44472222 | EF120567 | EF120540 |  |  |  |  |
| *Chersina angulata* |  | Stilbaai6 | Still Bay, WC | -34.25666667 | 21.44472222 | EF120568 | EF120540 |  |  |  |  |
| *Chersina angulata* |  | Stilbaai7 | Still Bay, WC | -34.25666667 | 21.44472222 | EF120567 | EF120540 |  |  |  |  |
| *Chersina angulata* |  | Stilbaai8 | Still Bay, WC | -34.25666667 | 21.44472222 | EF120567 |  |  |  |  |  |
| *Chersina angulata* |  | LB1 | Lamberts Bay, WC | -32.01194444 | 18.34111111 | EF120552 | EF120511 |  |  |  |  |
| *Chersina angulata* |  | LB2 | Lamberts Bay, WC | -32.01194444 | 18.34111111 | EF120551 |  |  |  |  |  |
| *Chersina angulata* |  | Springbok1 | Springbok, NC | -29.69194444 | 17.88305556 | EF120546 | EF120511 |  |  |  |  |
| *Chersina angulata* |  | Springbok2 | Springbok, NC | -29.69194444 | 17.88305556 | EF120546 | EF120511 |  |  |  |  |
| *Chersina angulata* |  | Springbok4 | Springbok, NC | -29.69194444 | 17.88305556 | EF120546 | EF120511 |  |  |  |  |
| *Chersina angulata* |  | Lutzville1 | Lutzville, WC | -31.50444444 | 18.29666667 | EF120551 | EF120515 |  |  |  |  |
| *Chersina angulata* |  | Doornbaai1 | Doorn Bay, WC | -31.7825 | 18.23277778 | EF120551 | EF120514 |  |  |  |  |
| *Chersina angulata* |  | EPNR1 | Elandsberg Private NR, WC | -33.42805556 | 19.02694444 | EF120557 | EF120524 |  |  |  |  |
| *Chersina angulata* |  | EPNR2 | Elandsberg Private NR, WC | -33.42805556 | 19.02694444 | EF120554 | EF120528 |  |  |  |  |
| *Chersina angulata* |  | EPNR3 | Elandsberg Private NR, WC | -33.42805556 | 19.02694444 | EF120557 |  |  |  |  |  |
| *Chersina angulata* |  | Kardoesie1 | Kardoesie, WC | -32.62861111 | 18.94833333 | EF120557 | EF120522 |  |  |  |  |
| *Chersina angulata* |  | Dassen1 | Dassen Island, WC | -33.4225 | 18.08805556 | EF120559 | EF120533 |  |  |  |  |
| *Chersina angulata* |  | Dassen2 | Dassen Island, WC | -33.4225 | 18.08805556 | EF120554 |  |  |  |  |  |
| *Chersina angulata* |  | Dassen3 | Dassen Island, WC | -33.4225 | 18.08805556 | EF120559 |  |  |  |  |  |
| *Chersina angulata* |  | Dassen4 | Dassen Island, WC | -33.4225 | 18.08805556 | EF120554 | EF120521 |  |  |  |  |
| *Chersina angulata* |  | Dassen5 | Dassen Island, WC | -33.4225 | 18.08805556 | EF120554 | EF120522 |  |  |  |  |
| *Chersina angulata* |  | Dassen6 | Dassen Island, WC | -33.4225 | 18.08805556 | EF120558 | EF120532 |  |  |  |  |
| *Chersina angulata* |  | Dassen7 | Dassen Island, WC | -33.4225 | 18.08805556 | EF120559 |  |  |  |  |  |
| *Chersina angulata* |  | Dassen8 | Dassen Island, WC | -33.4225 | 18.08805556 | EF120558 |  |  |  |  |  |
| *Chersina angulata* |  | Dassen9 | Dassen Island, WC | -33.4225 | 18.08805556 | EF120559 | EF120526 |  |  |  |  |
| *Chersina angulata* |  | Dassen10 | Dassen Island, WC | -33.4225 | 18.08805556 | EF120559 | EF120532 |  |  |  |  |
| *Chersina angulata* |  | Dassen11 | Dassen Island, WC | -33.4225 | 18.08805556 | EF120558 | EF120526 |  |  |  |  |
| *Chersina angulata* |  | Dassen12 | Dassen Island, WC | -33.4225 | 18.08805556 | EF120554 |  |  |  |  |  |
| *Chersina angulata* |  | Dassen14 | Dassen Island, WC | -33.4225 | 18.08805556 | EF120559 | EF120532 |  |  |  |  |
| *Chersina angulata* |  | Dassen15 | Dassen Island, WC | -33.4225 | 18.08805556 |  | EF120526 |  |  |  |  |
| *Chersina angulata* |  | Dassen16 | Dassen Island, WC | -33.4225 | 18.08805556 |  | EF120526 |  |  |  |  |
| *Chersina angulata* |  | Dassen17 | Dassen Island, WC | -33.4225 | 18.08805556 |  | EF120521 |  |  |  |  |
| *Chersina angulata* |  | Dassen18 | Dassen Island, WC | -33.4225 | 18.08805556 |  | EF120532 |  |  |  |  |
| *Chersina angulata* |  | Dassen19 | Dassen Island, WC | -33.4225 | 18.08805556 |  | EF120521 |  |  |  |  |
| *Chersina angulata* |  | Dassen20 | Dassen Island, WC | -33.4225 | 18.08805556 |  | EF120526 |  |  |  |  |
| *Chersina angulata* |  | Dassen21 | Dassen Island, WC | -33.4225 | 18.08805556 |  | EF120532 |  |  |  |  |
| *Chersina angulata* |  | PE1 | Gqeberha, EC | -34.01888889 | 25.4975 | EF120577 | EF120537 |  |  |  |  |
| *Chersina angulata* |  | PE2 | Gqeberha, EC | -34.01888889 | 25.4975 | EF120577 | EF120537 |  |  |  |  |
| *Chersina angulata* |  | PE3 | Gqeberha, EC | -34.01888889 | 25.4975 | EF120578 | EF120536 |  |  |  |  |
| *Chersina angulata* |  | PE4 | Gqeberha, EC | -34.01888889 | 25.4975 | EF120577 | EF120537 |  |  |  |  |
| *Chersina angulata* |  | PE5 | Gqeberha, EC | -34.01888889 | 25.4975 | EF120577 | EF120537 |  |  |  |  |
| *Chersina angulata* |  | PE6 | Gqeberha, EC | -34.01888889 | 25.4975 | EF120577 | EF120537 |  |  |  |  |
| *Chersina angulata* |  | PE7 | Gqeberha, EC | -34.01888889 | 25.4975 | EF120577 | EF120537 |  |  |  |  |
| *Chersina angulata* |  | PE8 | Gqeberha, EC | -34.01888889 | 25.4975 | EF120578 | EF120535 |  |  |  |  |
| *Chersina angulata* |  | PE9 | Gqeberha, EC | -34.01888889 | 25.4975 |  | EF120537 |  |  |  |  |
| *Chersina angulata* |  | PE10 | Gqeberha, EC | -34.01888889 | 25.4975 |  | EF120537 |  |  |  |  |
| *Chersina angulata* |  | PE11 | Gqeberha, EC | -34.01888889 | 25.4975 | EF120577 |  |  |  |  |  |
| *Chersina angulata* |  | PE12 | Gqeberha, EC | -34.01888889 | 25.4975 | EF120577 | EF120537 |  |  |  |  |
| *Chersina angulata* |  | PE13 | Gqeberha, EC | -34.01888889 | 25.4975 | EF120577 | EF120537 |  |  |  |  |
| *Chersina angulata* |  | PE14 | Gqeberha, EC | -34.01888889 | 25.4975 | EF120578 |  |  |  |  |  |
| *Chersina angulata* |  | PE15 | Gqeberha, EC | -34.01888889 | 25.4975 | EF120577 | EF120537 |  |  |  |  |
| *Chersina angulata* |  | PE17 | Gqeberha, EC | -34.01888889 | 25.4975 | EF120577 | EF120537 |  |  |  |  |
| *Chersina angulata* |  | PE18 | Gqeberha, EC | -34.01888889 | 25.4975 | EF120578 | EF120535 |  |  |  |  |
| *Chersina angulata* |  | PE19 | Gqeberha, EC | -34.01888889 | 25.4975 | EF120577 | EF120537 |  |  |  |  |
| *Chersina angulata* |  | PE20 | Gqeberha, EC | -34.01888889 | 25.4975 | EF120578 | EF120535 |  |  |  |  |
| *Chersina angulata* |  | Dans1 | Danskraal, NC | -29.70805556 | 17.80166667 | EF120546 | EF120511 |  |  |  |  |
| *Chersina angulata* |  | DanS1 | Danskraal, NC | -29.70805556 | 17.80166667 | EF120550 | EF120511 |  |  |  |  |
| *Chersina angulata* |  | Dans3 | Danskraal, NC | -29.70805556 | 17.80166667 | EF120546 | EF120511 |  |  |  |  |
| *Chersina angulata* |  | Dans4 | Danskraal, NC | -29.70805556 | 17.80166667 | EF120550 | EF120511 |  |  |  |  |
| *Chersina angulata* |  | Dans5 | Danskraal, NC | -29.70805556 | 17.80166667 | EF120550 | EF120511 |  |  |  |  |
| *Chersina angulata* |  | Dans6 | Danskraal, NC | -29.70805556 | 17.80166667 | EF120546 | EF120511 |  |  |  |  |
| *Chersina angulata* |  | Dans7 | Danskraal, NC | -29.70805556 | 17.80166667 | EF120546 | EF120511 |  |  |  |  |
| *Chersina angulata* |  | Dans8 | Danskraal, NC | -29.70805556 | 17.80166667 | EF120550 | EF120511 |  |  |  |  |
| *Chersina angulata* |  | Dans9 | Danskraal, NC | -29.70805556 | 17.80166667 | EF120550 | EF120511 |  |  |  |  |
| *Chersina angulata* |  | Dans10 | Danskraal, NC | -29.70805556 | 17.80166667 | EF120550 | EF120511 |  |  |  |  |
| *Chersina angulata* |  | Dans11 | Danskraal, NC | -29.70805556 | 17.80166667 | EF120550 | EF120511 |  |  |  |  |
| *Chersina angulata* |  | Dans12 | Danskraal, NC | -29.70805556 | 17.80166667 | EF120546 | EF120511 |  |  |  |  |
| *Chersina angulata* |  | Dans13 | Danskraal, NC | -29.70805556 | 17.80166667 | EF120550 | EF120511 |  |  |  |  |
| *Chersina angulata* |  | Dans14 | Danskraal, NC | -29.70805556 | 17.80166667 | EF120550 | EF120511 |  |  |  |  |
| *Chersina angulata* |  | Dans15 | Danskraal, NC | -29.70805556 | 17.80166667 | EF120550 | EF120511 |  |  |  |  |
| *Chersina angulata* |  | Dans16 | Danskraal, NC | -29.70805556 | 17.80166667 | EF120550 | EF120511 |  |  |  |  |
| *Chersina angulata* |  | Dans17 | Danskraal, NC | -29.70805556 | 17.80166667 | EF120550 |  |  |  |  |  |
| *Chersina angulata* |  | Dans18 | Danskraal, NC | -29.70805556 | 17.80166667 | EF120546 | EF120511 |  |  |  |  |
| *Chersina angulata* |  | Dans19 | Danskraal, NC | -29.70805556 | 17.80166667 | EF120546 | EF120511 |  |  |  |  |
| *Chersina angulata* |  | Dans20 | Danskraal, NC | -29.70805556 | 17.80166667 | EF120546 | EF120513 |  |  |  |  |
| *Chersina angulata* |  | Dans21 | Danskraal, NC | -29.70805556 | 17.80166667 | EF120546 | EF120511 |  |  |  |  |
| *Chersina angulata* |  | WCNP1 | West Coast NP, WC | -33.23416667 | 18.13361111 | EF120554 |  |  |  |  |  |
| *Chersina angulata* |  | WCNP2 | West Coast NP, WC | -33.23416667 | 18.13361111 | EF120558 |  |  |  |  |  |
| *Chersina angulata* |  | WCNP3 | West Coast NP, WC | -33.23416667 | 18.13361111 | EF120554 |  |  |  |  |  |
| *Chersina angulata* |  | WCNP4 | West Coast NP, WC | -33.23416667 | 18.13361111 | EF120560 | EF120526 |  |  |  |  |
| *Chersina angulata* |  | WCNP5 | West Coast NP, WC | -33.23416667 | 18.13361111 | EF120557 | EF120521 |  |  |  |  |
| *Chersina angulata* |  | WCNP6 | West Coast NP, WC | -33.23416667 | 18.13361111 | EF120557 |  |  |  |  |  |
| *Chersina angulata* |  | WCNP7 | West Coast NP, WC | -33.23416667 | 18.13361111 | EF120555 | EF120526 |  |  |  |  |
| *Chersina angulata* |  | WCNP8 | West Coast NP, WC | -33.23416667 | 18.13361111 | EF120554 |  |  |  |  |  |
| *Chersina angulata* |  | WCNP9 | West Coast NP, WC | -33.23416667 | 18.13361111 | EF120557 |  |  |  |  |  |
| *Chersina angulata* |  | WCNP10 | West Coast NP, WC | -33.23416667 | 18.13361111 | EF120554 | EF120532 |  |  |  |  |
| *Chersina angulata* |  | WCNP11 | West Coast NP, WC | -33.23416667 | 18.13361111 | EF120559 | EF120525 |  |  |  |  |
| *Chersina angulata* |  | WCNP12 | West Coast NP, WC | -33.23416667 | 18.13361111 | EF120554 | EF120522 |  |  |  |  |
| *Chersina angulata* |  | WCNP13 | West Coast NP, WC | -33.23416667 | 18.13361111 | EF120554 | EF120522 |  |  |  |  |
| *Chersina angulata* |  | WCNP14 | West Coast NP, WC | -33.23416667 | 18.13361111 | EF120559 | EF120526 |  |  |  |  |
| *Chersina angulata* |  | WCNP15 | West Coast NP, WC | -33.23416667 | 18.13361111 | EF120557 | EF120526 |  |  |  |  |
| *Chersina angulata* |  | WCNP16 | West Coast NP, WC | -33.23416667 | 18.13361111 | EF120554 | EF120527 |  |  |  |  |
| *Chersina angulata* |  | WCNP17 | West Coast NP, WC | -33.23416667 | 18.13361111 | EF120558 |  |  |  |  |  |
| *Chersina angulata* |  | WCNP18 | West Coast NP, WC | -33.23416667 | 18.13361111 | EF120559 | EF120532 |  |  |  |  |
| *Chersina angulata* |  | WCNP19 | West Coast NP, WC | -33.23416667 | 18.13361111 | EF120557 |  |  |  |  |  |
| *Chersina angulata* |  | WCNP20 | West Coast NP, WC | -33.23416667 | 18.13361111 | EF120554 | EF120526 |  |  |  |  |
| *Chersina angulata* |  | WCNP21 | West Coast NP, WC | -33.23416667 | 18.13361111 | EF120554 | EF120526 |  |  |  |  |
| *Chersina angulata* |  | WCNP22 | West Coast NP, WC | -33.23416667 | 18.13361111 | EF120559 | EF120534 |  |  |  |  |
| *Chersina angulata* |  | WCNP23 | West Coast NP, WC | -33.23416667 | 18.13361111 | EF120557 | EF120523 |  |  |  |  |
| *Chersina angulata* |  | WCNP24 | West Coast NP, WC | -33.23416667 | 18.13361111 | EF120557 |  |  |  |  |  |
| *Chersina angulata* |  | WCNP25 | West Coast NP, WC | -33.23416667 | 18.13361111 | EF120557 | EF120523 |  |  |  |  |
| *Geochelone radiata** |  |  |  |  |  |  | AY678358 |  |  |  |  |
| *Duberria lutrix* |  |  |  |  |  |  |  |  |  |  |  |
| *Duberria lutrix* | PEM R22526 | Ashton1 | Ashton, WC | -33.8346 | 20.05474722 | MK518169 | MK518081 |  |  |  | MK518260 |
| *Duberria lutrix* | PEM R22527 | Ashton2 | Ashton, WC | -33.8346 | 20.05474722 | MK518170 | MK518082 |  |  |  |  |
| *Duberria lutrix* | PEM R22528 | Ashton3 | Ashton, WC | -33.8346 | 20.05474722 | MK518171 | MK518083 |  |  |  |  |
| *Duberria lutrix* | PEM R22529 | Ashton4 | Ashton, WC | -33.8346 | 20.05474722 | MK518172 | MK518084 |  |  |  |  |
| *Duberria lutrix* | PEM R22530 | Ashton5 | Ashton, WC | -33.8346 | 20.05474722 | MK518173 | MK518085 |  |  |  |  |
| *Duberria lutrix* | PEM R22531 | Ashton6 | Ashton, WC | -33.8346 | 20.05474722 | MK518174 | MK518086 |  |  |  |  |
| *Duberria lutrix* | PEM R22532 | Ashton7 | Ashton, WC | -33.8346 | 20.05474722 | MK518175 | MK518087 |  |  |  |  |
| *Duberria lutrix* | PEM R22533 | Ashton8 | Ashton, WC | -33.8346 | 20.05474722 | MK518176 | MK518088 |  |  |  |  |
| *Duberria lutrix* |  | Bergvliet1 | Bergvliet, WC | -34.05774722 | 18.45173889 | MK518177 | MK518089 |  |  |  | MK518261 |
| *Duberria lutrix* | PEM R22515 | Caledon1 | Caledon, WC | -34.23098611 | 19.425375 | MK518178 | MK518090 |  |  |  | MK518263 |
| *Duberria lutrix* | SANBI 4547 | Flakkenberg1 | Vlakkenberg, WC | -34.02744722 | 18.39385278 | MK518180 | MK518093 |  |  |  | MK518287 |
| *Duberria lutrix* | PEM R22516 | Genadendal1 | Genadendal, WC | -34.04164167 | 19.56252222 | MK518181 | MK518094 |  |  |  | MK518265 |
| *Duberria lutrix* | PEM R22517 | Greyton1 | Greyton, WC | -34.05227222 | 19.61307222 | MK518182 | MK518096 |  |  |  | MK518266 |
| *Duberria lutrix* | PEM R22518 | Greyton2 | Greyton, WC | -34.05227222 | 19.61307222 | MK518183 | MK518097 |  |  |  |  |
| *Duberria lutrix* | PEM R22519 | Greyton3 | Greyton, WC | -34.05227222 | 19.61307222 | MK518184 | MK518098 |  |  |  |  |
| *Duberria lutrix* | SANBI 10847 | Herbertsdale1 | Herbertsdale, WC | -34.01701389 | 21.76673611 | MK518185 | MK518099 |  |  |  | MK518267 |
| *Duberria lutrix* | SABI 4474 | HopeFountain1 | Hope Fountain, EC | -33.52424722 | 26.40108611 | MK518187 | MK518101 |  |  |  | MK518269 |
| *Duberria lutrix* | SANBI 8108 | Humansdorp1 | Humansdorp, EC | -34.033225 | 24.76649722 | MK518188 | MK518102 |  |  |  | MK518270 |
| *Duberria lutrix* | PEM R22493 | Jacobsbaai1 | Jacobs Bay, WC | -32.96896389 | 17.89093056 | MK518189 | MK518103 |  |  |  | MK518271 |
| *Duberria lutrix* | SANBI 11300 | Kirstenbosch1 | Kirstenbosch, WC | -33.98635833 | 18.43671111 | MK518190 | MK518104 |  |  |  | MK518272 |
| *Duberria lutrix* | SANBI 1450 | Kirstenbosch2 | Kirstenbosch, WC | -33.98635833 | 18.43671111 | MK518191 | MK518105 |  |  |  |  |
| *Duberria lutrix* | SANBI 2785 | Kirstenbosch3 | Kirstenbosch, WC | -33.98635833 | 18.43671111 | MK518192 | MK518106 |  |  |  |  |
| *Duberria lutrix* |  | Kraaifontein1 | Kraaifontein, WC | -33.849275 | 18.71287222 | MK518200 | MK518107 |  |  |  |  |
| *Duberria lutrix* | SANBI 1703 | LakesideCT1 | Lakeside, WC | -34.08946944 | 18.45389444 | MK518202 | MK518119 |  |  |  | MK518277 |
| *Duberria lutrix* | PEM R22520 | Napier1 | Napier, WC | -34.46655556 | 19.89991944 | MK518203 | MK518120 |  |  |  | MK518278 |
| *Duberria lutrix* | PEM R22521 | Napier2 | Napier, WC | -34.46655556 | 19.89991944 | MK518204 | MK518121 |  |  |  |  |
| *Duberria lutrix* | PEM R22522 | Napier3 | Napier, WC | -34.46655556 | 19.89991944 | MK518205 | MK518122 |  |  |  |  |
| *Duberria lutrix* | PEM R22523 | Napier4 | Napier, WC | -34.46655556 | 19.89991944 | MK518206 | MK518123 |  |  |  |  |
| *Duberria lutrix* | PEM R22524 | Napier5 | Napier, WC | -34.46655556 | 19.89991944 | MK518207 | MK518124 |  |  |  |  |
| *Duberria lutrix* | PEM R22525 | Napier6 | Napier, WC | -34.46655556 | 19.89991944 | MK518208 | MK518125 |  |  |  |  |
| *Duberria lutrix* | SANBI 4558 | NaturesValley1 | Natures Valley, WC | -33.98066667 | 23.55633056 | MK518209 | MK518126 |  |  |  | MK518279 |
| *Duberria lutrix* | SANBI 2879 | Oudtshoorn1 | Oudtshoorn, WC | -33.66029722 | 22.173525 | MK518210 | MK518127 |  |  |  | MK518280 |
| *Duberria lutrix* | SANBI 400 | PortAlfred1 | Port Alfred, EC | -33.59322222 | 26.88431944 | MK518211 | MK518128 |  |  |  |  |
| *Duberria lutrix* | PEM R22506 | PringleBay1 | PringleBay, WC | -34.345525 | 18.8328 | MK518213 | MK518130 |  |  |  |  |
| *Duberria lutrix* | PEM R22507 | PringleBay2 | PringleBay, WC | -34.345525 | 18.8328 | MK518214 | MK518131 |  |  |  |  |
| *Duberria lutrix* | PEM R22508 | PringleBay3 | PringleBay, WC | -34.345525 | 18.8328 | MK518215 | MK518132 |  |  |  |  |
| *Duberria lutrix* | PEM R22509 | PringleBay4 | PringleBay, WC | -34.345525 | 18.8328 | MK518216 | MK518133 |  |  |  |  |
| *Duberria lutrix* | SANBI 4550 | Silvermine | Silvermine, WC | -34.09158056 | 18.42008889 | MK518219 | MK518136 |  |  |  |  |
| *Duberria lutrix* | PEM R22498 | SomersetWest1 | Somerset West, WC | -34.07676111 | 18.84468889 | MK518220 | MK518137 |  |  |  | MK518281 |
| *Duberria lutrix* | PEM R22499 | SomersetWest2 | Somerset West, WC | -34.07676111 | 18.84468889 | MK518221 | MK518138 |  |  |  |  |
| *Duberria lutrix* | PEM R22500 | SomersetWest3 | Somerset West, WC | -34.07676111 | 18.84468889 | MK518222 | MK518139 |  |  |  |  |
| *Duberria lutrix* | PEM R22501 | SomersetWest4 | Somerset West, WC | -34.07676111 | 18.84468889 | MK518223 | MK518140 |  |  |  |  |
| *Duberria lutrix* | PEM R22502 | SomersetWest5 | Somerset West, WC | -34.07676111 | 18.84468889 | MK518224 | MK518141 |  |  |  |  |
| *Duberria lutrix* | PEM R22503 | SomersetWest6 | Somerset West, WC | -34.07676111 | 18.84468889 | MK518225 | MK518142 |  |  |  |  |
| *Duberria lutrix* | PEM R22504 | SomersetWest7 | Somerset West, WC | -34.07676111 | 18.84468889 | MK518226 | MK518143 |  |  |  |  |
| *Duberria lutrix* | PEM R22505 | SomersetWest8 | Somerset West, WC | -34.07676111 | 18.84468889 | MK518251 | MK518144 |  |  |  |  |
| *Duberria lutrix* | PEM R22494 | Stellenbosch1 | Stellenbosch, WC | -33.90663889 | 18.85485278 | MK518227 | MK518145 |  |  |  | MK518282 |
| *Duberria lutrix* | PEM R22495 | Stellenbosch2 | Stellenbosch, WC | -33.90663889 | 18.85485278 | MK518228 | MK518146 |  |  |  |  |
| *Duberria lutrix* | PEM R22496 | Stellenbosch3 | Stellenbosch, WC | -33.90663889 | 18.85485278 | MK518229 | MK518147 |  |  |  |  |
| *Duberria lutrix* | PEM R22497 | Stellenbosch4 | Stellenbosch, WC | -33.90663889 | 18.85485278 | MK518230 | MK518148 |  |  |  |  |
| *Duberria lutrix* | PEM R22524 | Swellendam1 | Swellendam, WC | -34.02939167 | 20.44516389 | MK518253 | MK518150 |  |  |  | MK518284 |
| *Duberria lutrix* | PEM R22525 | Swellendam2 | Swellendam, WC | -34.02939167 | 20.44516389 | MK518254 | MK518151 |  |  |  |  |
| *Duberria lutrix* | PEM R22526 | Swellendam3 | Swellendam, WC | -34.02939167 | 20.44516389 | MK518255 | MK518152 |  |  |  |  |
| *Duberria lutrix* | PEM R22527 | Swellendam4 | Swellendam, WC | -34.02939167 | 20.44516389 | MK518256 | MK518153 |  |  |  |  |
| *Duberria lutrix* | PEM R22528 | Swellendam5 | Swellendam, WC | -34.02939167 | 20.44516389 | MK518257 | MK518154 |  |  |  |  |
| *Duberria lutrix* | PEM R22529 | Swellendam6 | Swellendam, WC | -34.02939167 | 20.44516389 | MK518258 | MK518155 |  |  |  |  |
| *Duberria lutrix* |  | Swellendam7 | Swellendam, WC | -34.02939167 | 20.44516389 |  | MK518156 |  |  |  |  |
| *Duberria lutrix* | SANBI 4545 | Tokai1 | Tokai, WC | -34.06038889 | 18.42905833 | MK518232 | MK518157 |  |  |  | MK518285 |
| *Duberria lutrix* | PEM R22510 | Villiersdorp1 | Villiersdorp, WC | -33.98579444 | 19.28616111 | MK518234 | MK518159 |  |  |  | MK518286 |
| *Duberria lutrix* | PEM R22511 | Villiersdorp2 | Villiersdorp, WC | -33.98579444 | 19.28616111 | MK518235 | MK518160 |  |  |  |  |
| *Duberria lutrix* | PEM R22512 | Villiersdorp3 | Villiersdorp, WC | -33.98579444 | 19.28616111 | MK518236 | MK518161 |  |  |  |  |
| *Duberria lutrix* | PEM R22513 | Villiersdorp4 | Villiersdorp, WC | -33.98579444 | 19.28616111 | MK518237 | MK518162 |  |  |  |  |
| *Duberria lutrix* | PEM R22514 | Villiersdorp5 | Villiersdorp, WC | -33.98579444 | 19.28616111 | MK518238 | MK518163 |  |  |  |  |
| *Duberria lutrix* |  | PortElizabeth1 | Gqberha, EC | -33.79799722 | 25.67187222 | FJ404356 | FJ494305 |  |  |  |  |
| *Duberria lutrix* |  | Agulhas1 | Agulhas, WC | -34.80311389 | 19.95990833 | MK518243 | MK518078 |  |  |  | MK518259 |
| *Duberria lutrix* |  | Agulhas2 | Agulhas, WC | -34.80311389 | 19.95990833 | MK518244 | MK518079 |  |  |  |  |
| *Duberria lutrix* |  | Agulhas3 | Agulhas, WC | -34.80311389 | 19.95990833 | MK518245 | MK518080 |  |  |  |  |
| *Duberria lutrix* |  | Bredasdorp1 | Bredasdorp, WC | -34.50578889 | 20.06725556 | MK518246 | MK518090 |  |  |  | MK518262 |
| *Duberria lutrix* |  | Grahamstown1 | Grahamstown, EC | -33.29996944 | 26.52718889 | MK518247 | MK518095 |  |  |  |  |
| *Duberria lutrix* |  | Klipheuwel1 | Klipheuwel, WC | -33.69622778 | 18.72415833 | MK518249 | MK518108 |  |  |  | MK518274 |
| *Duberria lutrix* |  | Klipheuwel2 | Klipheuwel, WC | -33.69622778 | 18.72415833 | MK518250 | MK518109 |  |  |  |  |
| *Duberria lutrix* |  | Struisbaai1 | Struisbaai, WC | -34.76166667 | 20.04080556 | MK518252 | MK518149 |  |  |  | MK518283 |
| *Duberria lutrix* |  | Kleinmond1 | Kleinmond, WC | -34.33501 | 19.01223 | MK518248 | MK518107 |  |  |  | MK518273 |
| *Duberria variegata** |  | D.VARIEGATA |  |  |  | FJ404357 | FJ404306 |  |  |  |  |
| *Acontias meleagris* |  |  |  |  |  |  |  |  |  |  |  |
| *Acontias meleagris* |  | Bwes | Beaufort West, WC | -32.321111 | 22.486111 |  |  |  | EU855676 |  |  |
| *Acontias meleagris* |  | Kuils1 | Kuilsrivier, WC | -33.91666667 | 18.66666667 |  |  |  | EU855677 |  |  |
| *Acontias meleagris* |  | Ouds1 | Oudtshoorn, WC | -33.58333333 | 22.21666667 |  |  |  | EU855678 |  |  |
| *Acontias meleagris* |  | Ouds2 | Oudtshoorn, WC | -33.58333333 | 22.21666667 |  |  |  | EU855679 |  |  |
| *Acontias meleagris* |  | Ouds3 | Oudtshoorn, WC | -33.58333333 | 22.21666667 |  |  |  | EU855680 |  |  |
| *Acontias meleagris* |  | Ouds4 | Oudtshoorn, WC | -33.58333333 | 22.21666667 |  |  |  | EU855681 |  |  |
| *Acontias meleagris* |  | Ouds5 | Oudtshoorn, WC | -33.58333333 | 22.21666667 |  |  |  | EU855682 |  |  |
| *Acontias meleagris* |  | Ouds6 | Oudtshoorn, WC | -33.58333333 | 22.21666667 |  |  |  | EU855683 |  |  |
| *Acontias meleagris* |  | Clanwil1 | Clanwilliam, WC | -32.15194444 | 18.88722222 |  |  |  | EU855684 |  |  |
| *Acontias meleagris* |  | Clanwil2 | Clanwilliam, WC | -32.15194444 | 18.88722222 |  |  |  | EU855685 |  |  |
| *Acontias meleagris* |  | Clanwil3 | Clanwilliam, WC | -32.15194444 | 18.88722222 |  |  |  | EU855686 |  |  |
| *Acontias meleagris* |  | Graaf1 | Graafwater, WC | -32.1175 | 18.63555556 |  |  |  | EU855687 |  |  |
| *Acontias meleagris* |  | Graaf2 | Graafwater, WC | -32.1175 | 18.63555556 |  |  |  | EU855688 |  |  |
| *Acontias meleagris* |  | Malmesb | Malmesbury, WC | -33.45 | 18.73777778 |  |  |  | EU855689 |  |  |
| *Acontias meleagris* |  | Lang1 | Langebaan, WC | -33.10444444 | 18.05166667 |  |  |  | EU855690 |  |  |
| *Acontias meleagris* |  | Lang2 | Langebaan, WC | -33.10444444 | 18.05166667 |  |  |  | EU855691 |  |  |
| *Acontias meleagris* |  | Lang3 | Langebaan, WC | -33.10444444 | 18.05166667 |  |  |  | EU855692 |  |  |
| *Acontias meleagris* |  | Lang4 | Langebaan, WC | -33.10444444 | 18.05166667 |  |  |  | EU855693 |  |  |
| *Acontias meleagris* |  | Lang5 | Langebaan, WC | -33.10444444 | 18.05166667 |  |  |  | EU855694 |  |  |
| *Acontias meleagris* |  | A.m.V1 | Velddrift, WC | -32.81694444 | 18.11722222 |  |  |  |  |  |  |
| *Acontias meleagris* |  | A.m.V2 | Velddrift, WC | -32.81694444 | 18.11722222 |  |  |  |  |  |  |
| *Acontias meleagris* |  | A.m.V3 | Velddrift, WC | -32.81694444 | 18.11722222 |  |  |  | AY683782 |  |  |
| *Acontias meleagris* |  | A.m.V4 | Velddrift, WC | -32.81694444 | 18.11722222 |  |  |  | AY683783 |  |  |
| *Acontias meleagris* |  | A.m.V5 | Velddrift, WC | -32.81694444 | 18.11722222 |  |  |  | AY683784 |  |  |
| *Acontias meleagris* |  | A.m.V6 | Velddrift, WC | -32.81694444 | 18.11722222 |  |  |  | AY683785 |  |  |
| *Acontias meleagris* |  | A.m.V7 | Velddrift, WC | -32.81694444 | 18.11722222 |  |  |  | AY683786 |  |  |
| *Acontias meleagris* |  | A.m.V8 | Velddrift, WC | -32.81694444 | 18.11722222 |  |  |  |  |  |  |
| *Acontias meleagris* |  | A.m.V9 | Velddrift, WC | -32.81694444 | 18.11722222 |  |  |  |  |  |  |
| *Acontias meleagris* |  | A.m.V10 | Velddrift, WC | -32.81694444 | 18.11722222 |  |  |  | AY028855 |  |  |
| *Acontias meleagris* |  | Veld1 | Velddrift, WC | -32.81694444 | 18.11722222 |  |  |  | EU855695 |  |  |
| *Acontias meleagris* |  | Veld2 | Velddrift, WC | -32.81694444 | 18.11722222 |  |  |  | EU855696 |  |  |
| *Acontias meleagris* |  | Veld3 | Velddrift, WC | -32.81694444 | 18.11722222 |  |  |  | EU855697 |  |  |
| *Acontias meleagris* |  | Veld4 | Velddrift, WC | -32.81694444 | 18.11722222 |  |  |  | EU855698 |  |  |
| *Acontias meleagris* |  | Veld5 | Velddrift, WC | -32.81694444 | 18.11722222 |  |  |  | EU855699 |  |  |
| *Acontias meleagris* |  | Veld6 | Velddrift, WC | -32.81694444 | 18.11722222 |  |  |  | EU855700 |  |  |
| *Acontias meleagris* |  | Veld7 | Velddrift, WC | -32.81694444 | 18.11722222 |  |  |  | EU855701 |  |  |
| *Acontias meleagris* |  | Veld8 | Velddrift, WC | -32.81694444 | 18.11722222 |  |  |  | EU855702 |  |  |
| *Acontias meleagris* |  | Veld2.1 | Velddrift, WC | -32.81694444 | 18.11722222 |  |  |  | EU855703 |  |  |
| *Acontias meleagris* |  | Veld2.2 | Velddrift, WC | -32.81694444 | 18.11722222 |  |  |  | EU855704 |  |  |
| *Acontias meleagris* |  | Veld2.3 | Velddrift, WC | -32.81694444 | 18.11722222 |  |  |  | EU855705 |  |  |
| *Acontias meleagris* |  | Veld2.4 | Velddrift, WC | -32.81694444 | 18.11722222 |  |  |  | EU855706 |  |  |
| *Acontias meleagris* |  | Veld2.5 | Velddrift, WC | -32.81694444 | 18.11722222 |  |  |  | EU855707 |  |  |
| *Acontias meleagris* |  | Veld2.6 | Velddrift, WC | -32.81694444 | 18.11722222 |  |  |  | EU855708 |  |  |
| *Acontias meleagris* |  | Veld2.7 | Velddrift, WC | -32.81694444 | 18.11722222 |  |  |  | EU855709 |  |  |
| *Acontias meleagris* |  | Veld2.8 | Velddrift, WC | -32.81694444 | 18.11722222 |  |  |  | EU855710 |  |  |
| *Acontias meleagris* |  | Veld2.9 | Velddrift, WC | -32.81694444 | 18.11722222 |  |  |  | EU855711 |  |  |
| *Acontias meleagris* |  | Veld2.10 | Velddrift, WC | -32.81694444 | 18.11722222 |  |  |  | EU855712 |  |  |
| *Acontias meleagris* |  | Veld2.11 | Velddrift, WC | -32.81694444 | 18.11722222 |  |  |  | EU855713 |  |  |
| *Acontias meleagris* |  | Veld2.12 | Velddrift, WC | -32.81694444 | 18.11722222 |  |  |  | EU855714 |  |  |
| *Acontias meleagris* |  | A.m.o1 | Grahamstown, EC | -33.27028 | 26.53611 |  |  |  | AY683737 |  |  |
| *Acontias meleagris* |  | A.m.o2 | Grahamstown, EC | -33.27028 | 26.53611 |  |  |  | AY683738 |  |  |
| *Acontias meleagris* |  | A.m.o3 | Grahamstown, EC | -33.27028 | 26.53611 |  |  |  | AY683739 |  |  |
| *Acontias meleagris* |  | A.m.o4 | Grahamstown, EC | -33.27028 | 26.53611 |  |  |  | AY683740 |  |  |
| *Acontias meleagris* |  | A.m.o5 | Grahamstown, EC | -33.27028 | 26.53611 |  |  |  | AY683741 |  |  |
| *Acontias meleagris* |  | A.m.o6 | Grahamstown, EC | -33.27028 | 26.53611 |  |  |  | AY683742 |  |  |
| *Acontias meleagris* |  | A.m.o7 | Grahamstown, EC | -33.27028 | 26.53611 |  |  |  | AY683743 |  |  |
| *Acontias meleagris* |  | A.m.o8 | Grahamstown, EC | -33.27028 | 26.53611 |  |  |  | AY683764 |  |  |
| *Acontias meleagris* |  | A.m.o9 | Grahamstown, EC | -33.27028 | 26.53611 |  |  |  | AY683765 |  |  |
| *Acontias meleagris* |  | A.m.o10 | Grahamstown, EC | -33.27028 | 26.53611 |  |  |  | AY683773 |  |  |
| *Acontias percivali tasmani* |  | A.p.t1 | Coega, EC | -33.75444 | 25.60333 |  |  |  | AY683744 |  |  |
| *Acontias percivali tasmani* |  | A.p.t2 | Coega, EC | -33.75444 | 25.60333 |  |  |  | AY683745 |  |  |
| *Acontias percivali tasmani* |  | A.p.t3 | Coega, EC | -33.75444 | 25.60333 |  |  |  | AY683746 |  |  |
| *Acontias percivali tasmani* |  | A.p.t4 | Coega, EC | -33.75444 | 25.60333 |  |  |  | AY683747 |  |  |
| *Acontias percivali tasmani* |  | A.p.t5 | Coega, EC | -33.75444 | 25.60333 |  |  |  | AY683748 |  |  |
| *Acontias percivali tasmani* |  | A.p.t6 | Coega, EC | -33.75444 | 25.60333 |  |  |  | AY028863 |  |  |
| *Acontias percivali tasmani* |  | A.p.t7 | Coega, EC | -33.75444 | 25.60333 |  |  |  | AY028864 |  |  |
| *Acontias percivali tasmani* |  | A.p.t8 | Coega, EC | -33.75444 | 25.60333 |  |  |  | AY028865 |  |  |
| *Acontias meleagris orientalis* |  | A.o.lPE1 | Gqeberha, EC | -33.70389 | 25.85222 |  |  |  | AY683759 |  |  |
| *Acontias meleagris orientalis* |  | A.o.lPE2 | Gqeberha, EC | -33.70389 | 25.85222 |  |  |  | AY683760 |  |  |
| *Acontias meleagris orientalis* |  | A.o.lPE3 | Gqeberha, EC | -33.70389 | 25.85222 |  |  |  | AY683761 |  |  |
| *Acontias meleagris orientalis* |  | A.o.lPE4 | Gqeberha, EC | -33.70389 | 25.85222 |  |  |  | AY683762 |  |  |
| *Acontias meleagris orientalis* |  | A.o.lPE5 | Gqeberha, EC | -33.70389 | 25.85222 |  |  |  | AY683763 |  |  |
| *Acontias meleagris orientalis* |  | A.o.lPA1 | Port Alfred, EC | -33.58333 | 26.91667 |  |  |  | AY683766 |  |  |
| *Acontias meleagris orientalis* |  | A.o.lPA2 | Port Alfred, EC | -33.58333 | 26.91667 |  |  |  | AY683767 |  |  |
| *Acontias meleagris orientalis* |  | A.o.lPA3 | Port Alfred, EC | -33.58333 | 26.91667 |  |  |  | AY683768 |  |  |
| *Acontias meleagris orientalis* |  | A.o.lPA4 | Port Alfred, EC | -33.58333 | 26.91667 |  |  |  | AY683769 |  |  |
| *Acontias meleagris orientalis* |  | A.o.lPA5 | Port Alfred, EC | -33.58333 | 26.91667 |  |  |  | AY683770 |  |  |
| *Acontias meleagris orientalis* |  | A.o.lPA6 | Port Alfred, EC | -33.58333 | 26.91667 |  |  |  | AY683771 |  |  |
| *Acontias meleagris orientalis* |  | A.o.lPA7 | Port Alfred, EC | -33.58333 | 26.91667 |  |  |  | AY683772 |  |  |
| *Acontias meleagris orientalis* |  | A.o.lPA8 | Port Alfred, EC | -33.58333 | 26.91667 |  |  |  | AY683773 |  |  |
| *Acontias meleagris* |  | A.m.M1 | Mossel Bay, WC | -34.16667 | 22.13333 |  |  |  | AY683774 |  |  |
| *Acontias meleagris* |  | A.m.M2 | Mossel Bay, WC | -34.16667 | 22.13333 |  |  |  | AY683775 |  |  |
| *Acontias meleagris* |  | A.m.M3 | Mossel Bay, WC | -34.16667 | 22.13333 |  |  |  | AY683776 |  |  |
| *Acontias meleagris* |  | A.m.M4 | Mossel Bay, WC | -34.16667 | 22.13333 |  |  |  | AY683777 |  |  |
| *Acontias meleagris* |  | A.m.M5 | Mossel Bay, WC | -34.16667 | 22.13333 |  |  |  | AY683778 |  |  |
| *Acontias meleagris* |  | A.m.M6 | Mossel Bay, WC | -34.16667 | 22.13333 |  |  |  | AY683779 |  |  |
| *Acontias meleagris* |  | A.m.M7 | Mossel Bay, WC | -34.16667 | 22.13333 |  |  |  | AY683780 |  |  |
| *Acontias meleagris* |  | Elands1 | Elands Bay, WC | -32.3025 | 18.33666667 |  |  |  | AY028854 |  |  |
| *Acontias meleagris* |  | Elands2 | Elands Bay, WC | -32.3025 | 18.33666667 |  |  |  | EU855715 |  |  |
| *Acontias meleagris* |  | SirLouw | Sir Lowry's Pass, WC | -34.1 | 18.91666667 |  |  |  | EU855717 |  |  |
| *Acontias meleagris* |  | Saldanha | Saldanha Bay, WC | -33.01666667 | 17.91666667 |  |  |  | EU855718 |  |  |
| *Acontias meleagris* |  | Struisba | Struis Bay, WC | -34.66666667 | 20.21666667 |  |  |  | EU855719 |  |  |
| *Acontias meleagris* |  | A.m.mR1 | Robben Island, WC | -33.8 | 18.36666667 |  |  |  | AY683754 |  |  |
| *Acontias meleagris* |  | A.m.mR2 | Robben Island, WC | -33.8 | 18.36666667 |  |  |  | AY683755 |  |  |
| *Acontias meleagris* |  | A.m.mR3 | Robben Island, WC | -33.8 | 18.36666667 |  |  |  | AY683756 |  |  |
| *Acontias meleagris* |  | A.m.mR4 | Robben Island, WC | -33.8 | 18.36666667 |  |  |  | AY683757 |  |  |
| *Acontias meleagris* |  | RobI1 | Robben Island, WC | -33.8 | 18.36666667 |  |  |  | AY683758 |  |  |
| *Acontias meleagris* |  | RobI2 | Robben Island, WC | -33.8 | 18.36666667 |  |  |  | EU855720 |  |  |
| *Acontias meleagris* |  | RobI3 | Robben Island, WC | -33.8 | 18.36666667 |  |  |  | EU855721 |  |  |
| *Acontias meleagris* |  | RobI4 | Robben Island, WC | -33.8 | 18.36666667 |  |  |  | EU855722 |  |  |
| *Acontias meleagris* |  | RobI5 | Robben Island, WC | -33.8 | 18.36666667 |  |  |  | EU855723 |  |  |
| *Acontias meleagris* |  | RobI6 | Robben Island, WC | -33.8 | 18.36666667 |  |  |  | EU855724 |  |  |
| *Acontias meleagris* |  | RobI7 | Robben Island, WC | -33.8 | 18.36666667 |  |  |  | EU855725 |  |  |
| *Acontias meleagris* |  | RobI8 | Robben Island, WC | -33.8 | 18.36666667 |  |  |  | EU855726 |  |  |
| *Acontias meleagris* |  | RobI9 | Robben Island, WC | -33.8 | 18.36666667 |  |  |  | EU855727 |  |  |
| *Acontias meleagris* |  | RobI10 | Robben Island, WC | -33.8 | 18.36666667 |  |  |  | EU855728 |  |  |
| *Acontias meleagris* |  | RobI11 | Robben Island, WC | -33.8 | 18.36666667 |  |  |  | EU855729 |  |  |
| *Acontias meleagris* |  | RobI12 | Robben Island, WC | -33.8 | 18.36666667 |  |  |  | EU855730 |  |  |
| *Acontias meleagris* |  | RobI13 | Robben Island, WC | -33.8 | 18.36666667 |  |  |  | EU855731 |  |  |
| *Acontias meleagris* |  | RobI14 | Robben Island, WC | -33.8 | 18.36666667 |  |  |  | EU855732 |  |  |
| *Acontias meleagris* |  | RobI15 | Robben Island, WC | -33.8 | 18.36666667 |  |  |  | EU855733 |  |  |
| *Acontias meleagris* |  | CapeHang | Cape Hangklip, WC | -34.38333333 | 18.83333333 |  |  |  | EU855734 |  |  |
| *Acontias meleagris* |  | Jacobs1 | Jacobs Bay, WC | -32.96666667 | 17.88333333 |  |  |  | EU855735 |  |  |
| *Acontias meleagris* |  | Jacobs2 | Jacobs Bay, WC | -32.96666667 | 17.88333333 |  |  |  | EU855736 |  |  |
| *Acontias meleagris* |  | Aberdeen133 | Aberdeen, EC | -32.3525 | 24.585666 |  |  |  | JQ692328 |  | JQ278040 |
| *Acontias meleagris* |  | Aberdeen135 | Aberdeen, EC | -32.3525 | 24.585666 |  |  |  | JQ692329 |  |  |
| *Acontias meleagris* |  | Aberdeen136 | Aberdeen, EC | -32.3525 | 24.585666 |  |  |  | JQ692330 |  |  |
| *Acontias meleagris* |  | Aberdeen137 | Aberdeen, EC | -32.3525 | 24.585666 |  |  |  | JQ692331 |  |  |
| *Acontias meleagris* |  | Aberdeen138 |  |  |  |  |  |  |  |  | JQ278041 |
| *Acontias meleagris* |  | Aberdeen140 | Aberdeen, EC | -32.3525 | 24.585666 |  |  |  | JQ692332 |  | JQ278042 |
| *Acontias meleagris* |  | Aberdeen144 | Aberdeen, EC | -32.3525 | 24.585666 |  |  |  | JQ692333 |  |  |
| *Acontias meleagris* |  | Agulhas224 |  |  |  |  |  |  |  |  | JQ278043 |
| *Acontias meleagris* |  | Agulhas228 | Agulhas, WC | -34.62673 | 20.1207 |  |  |  | JQ692334 |  |  |
| *Acontias meleagris* |  | ORAlex | Alexandria, EC | -33.671783 | 26.417313 |  |  |  | JQ692335 |  | JQ278044 |
| *Acontias meleagris* |  | Alexandria182 | Alexandria, EC | -33.671783 | 26.417313 |  |  |  | JQ692336 |  | JQ278045 |
| *Acontias meleagris* |  | Alexandria184 | Alexandria, EC | -33.671783 | 26.417313 |  |  |  | JQ692337 |  | JQ278046 |
| *Acontias meleagris* |  | Ashton2 | Ashton, WC | -34.226666 | 19.416666 |  |  |  | JQ692338 |  |  |
| *Acontias meleagris* |  | Ashton3 | Ashton, WC | -34.226666 | 19.416666 |  |  |  | JQ692339 |  | JQ278047 |
| *Acontias meleagris* |  | Ashton4 | Ashton, WC | -34.226666 | 19.416666 |  |  |  | JQ692340 |  | JQ278048 |
| *Acontias meleagris* |  | Ashton5 | Ashton, WC | -34.226666 | 19.416666 |  |  |  | JQ692341 |  | JQ278049 |
| *Acontias meleagris* |  | Aurora | Aurora, WC | -32.7083 | 18.484154 |  |  |  | JQ692342 |  | JQ278050 |
| *Acontias meleagris* |  | Baakinsvalley | Baakens Valley, EC | -33.733477 | 25.477295 |  |  |  | JQ692343 |  |  |
| *Acontias meleagris* |  | ORBarry | Barrydale, WC | -33.838333 | 20.033333 |  |  |  | JQ692344 |  | JQ278051 |
| *Acontias meleagris* |  | OSBarry1 | Barrydale, WC | -33.838333 | 20.033333 |  |  |  | JQ692345 |  | JQ278052 |
| *Acontias meleagris* |  | OSBarry2 | Barrydale, WC | -33.838333 | 20.033333 |  |  |  | JQ692346 |  |  |
| *Acontias meleagris* |  | OSBarry3 | Barrydale, WC | -33.838333 | 20.033333 |  |  |  | JQ692347 |  |  |
| *Acontias meleagris* |  | OSBarry4 | Barrydale, WC | -33.838333 | 20.033333 |  |  |  | JQ692348 |  |  |
| *Acontias meleagris* |  | OSBarry5 | Barrydale, WC | -33.838333 | 20.033333 |  |  |  | JQ692349 |  | JQ278053 |
| *Acontias meleagris* |  | ORBedford79 | Bedford, EC | -33.068666 | 26.23 |  |  |  | JQ692350 |  |  |
| *Acontias meleagris* |  | ORBedford80 | Bedford, EC | -33.068666 | 26.23 |  |  |  | JQ692351 |  |  |
| *Acontias meleagris* |  | ORBedford81 | Bedford, EC | -33.068666 | 26.23 |  |  |  | JQ692352 |  |  |
| *Acontias meleagris* |  | ORBedford82 | Bedford, EC | -33.068666 | 26.23 |  |  |  | JQ692353 |  |  |
| *Acontias meleagris* |  | ORBedford83 | Bedford, EC | -33.068666 | 26.23 |  |  |  | JQ692354 |  | JQ278054 |
| *Acontias meleagris* |  | ORBedford84 | Bedford, EC | -33.068666 | 26.23 |  |  |  | JQ692355 |  |  |
| *Acontias meleagris* |  | ORBedford85 | Bedford, EC | -33.068666 | 26.23 |  |  |  | JQ692356 |  |  |
| *Acontias meleagris* |  | BettiesBay | Betties Bay | 0 |  |  |  |  | JQ692357 |  | JQ278055 |
| *Acontias meleagris* |  | Bloemhof104 | Bloemhof, EC | -32.825 | 25.725 |  |  |  | JQ692358 |  | JQ278056 |
| *Acontias meleagris* |  | Bloemhof105 | Bloemhof, EC | -32.825 | 25.725 |  |  |  | JQ692359 |  | JQ278058 |
| *Acontias meleagris* |  | Bloemhof106 | Bloemhof, EC | -32.825 | 25.725 |  |  |  | JQ692360 |  | JQ278057 |
| *Acontias meleagris* |  | Bloemhof108 | Bloemhof, EC | -32.825 | 25.725 |  |  |  | JQ692361 |  |  |
| *Acontias meleagris* |  | Bloemhof110 | Bloemhof, EC | -32.825 | 25.725 |  |  |  | JQ692362 |  | JQ278059 |
| *Acontias meleagris* |  | Bloemhof111 | Bloemhof, EC | -32.825 | 25.725 |  |  |  | JQ692363 |  |  |
| *Acontias meleagris* |  | Bloemhof112 | Bloemhof, EC | -32.825 | 25.725 |  |  |  | JQ692364 |  | JQ278060 |
| *Acontias meleagris* |  | Bloemhof114 | Bloemhof, EC | -32.825 | 25.725 |  |  |  | JQ692365 |  | JQ278061 |
| *Acontias meleagris* |  | Bloemhof119 | Bloemhof, EC | -32.825 | 25.725 |  |  |  | JQ692366 |  | JQ278062 |
| *Acontias meleagris* |  | Bloemhof120 | Bloemhof, EC | -32.825 | 25.725 |  |  |  | JQ692367 |  | JQ278063 |
| *Acontias meleagris* |  | Bredasdorp191 | Bredasdorp, WC | -34.533333 | 20.041667 |  |  |  | JQ692368 |  |  |
| *Acontias meleagris* |  | Bredasdorp192 | Bredasdorp, WC | -34.533333 | 20.041667 |  |  |  | JQ692369 |  | JQ278064 |
| *Acontias meleagris* |  | Bredasdorp193 | Bredasdorp, WC | -34.533333 | 20.041667 |  |  |  | JQ692370 |  | JQ278065 |
| *Acontias meleagris* |  | Bredasdorp197 | Bredasdorp, WC | -34.533333 | 20.041667 |  |  |  | JQ692371 |  |  |
| *Acontias meleagris* |  | Bredasdorp201 | Bredasdorp, WC | -34.533333 | 20.041667 |  |  |  | JQ692373 |  |  |
| *Acontias meleagris* |  | Bredarsdorp212 |  |  |  |  |  |  |  |  | JQ278066 |
| *Acontias meleagris* |  | Bredasdorp214 | Bredasdorp, WC | -34.533333 | 20.041667 |  |  |  | JQ692373 |  |  |
| *Acontias meleagris* |  | Bredasdorp218 | Bredasdorp, WC | -34.533333 | 20.041667 |  |  |  | JQ692374 |  |  |
| *Acontias meleagris* |  | Cookhouse98 | Cookhouse, EC | -32.59 | 25.135833 |  |  |  | JQ692375 |  | JQ278067 |
| *Acontias meleagris* |  | Cookhouse100 | Cookhouse, EC | -32.59 | 25.135833 |  |  |  | JQ692376 |  | JQ278068 |
| *Acontias meleagris* |  | Cookhouse101 | Cookhouse, EC | -32.59 | 25.135833 |  |  |  | JQ692377 |  | JQ278069 |
| *Acontias meleagris* |  | Cookhouse102 | Cookhouse, EC | -32.59 | 25.135833 |  |  |  | JQ692378 |  |  |
| *Acontias meleagris* |  | ORTCradock155 | Cradock, EC | -32.158756 | 25.613251 |  |  |  | JQ692379 |  |  |
| *Acontias meleagris* |  | ORTCradock156 | Cradock, EC | -32.158756 | 25.613251 |  |  |  | JQ692380 |  | JQ278070 |
| *Acontias meleagris* |  | ORTCradock158 | Cradock, EC | -32.158756 | 25.613251 |  |  |  | JQ692381 |  |  |
| *Acontias meleagris* |  | Dunbrody1 | Dunbrody, WC | -33.471666 | 25.541666 |  |  |  | JQ692382 |  |  |
| *Acontias meleagris* |  | Dunbrody2 | Dunbrody, WC | -33.471666 | 25.541666 |  |  |  | JQ692383 |  |  |
| *Acontias meleagris* |  | Dunbrody3 | Dunbrody, WC | -33.471666 | 25.541666 |  |  |  | n/a |  | JQ278071 |
| *Acontias meleagris* |  | Dunbrody5 | Dunbrody, WC | -33.471666 | 25.541666 |  |  |  | JQ692385 |  |  |
| *Acontias meleagris* |  | ELondon1 | East London, EC | -33.058333 | 27.852166 |  |  |  | JQ692386 |  |  |
| *Acontias meleagris* |  | ELondon2 | East London, EC | -33.058333 | 27.852166 |  |  |  | JQ692387 |  |  |
| *Acontias meleagris* |  | EastLondon70 | East London, EC | -33.058333 | 27.852166 |  |  |  | JQ692388 |  | JQ278073 |
| *Acontias meleagris* |  | OSEastLond74 | East London, EC | -33.058333 | 27.852166 |  |  |  | JQ692389 |  | JQ278072 |
| *Acontias meleagris* |  | Gansbaai | Gans Bay, WC | -34.583917 | 19.346881 |  |  |  | JQ692390 |  | JQ278074 |
| *Acontias meleagris* |  | GraaffRein127 | Graaf Reinet, EC | -32.701833 | 25.635166 |  |  |  | JQ692391 |  |  |
| *Acontias meleagris* |  | GraaffRein128 | Graaf Reinet, EC | -32.701833 | 25.635166 |  |  |  | JQ692392 |  | JQ278075 |
| *Acontias meleagris* |  | Hfountain1 | Hope Fountain, WC | -33.471666 | 25.541666 |  |  |  | JQ692393 |  | JQ278077 |
| *Acontias meleagris* |  | Hfountain2 | Hope Fountain, WC | -33.471666 | 25.541666 |  |  |  | JQ692394 |  | JQ278078 |
| *Acontias meleagris* |  | Hfountain3 | Hope Fountain, WC | -33.471666 | 25.541666 |  |  |  | JQ692395 |  |  |
| *Acontias meleagris* |  | ORJansenville168 | Jansenville, EC | -32.638333 | 24.685 |  |  |  | JQ692396 |  |  |
| *Acontias meleagris* |  | ORJansenville169 | Jansenville, EC | -32.638333 | 24.685 |  |  |  | JQ692397 |  | JQ278079 |
| *Acontias meleagris* |  | ORJansenvil170 | Jansenville, EC | -32.638333 | 24.685 |  |  |  | JQ692398 |  |  |
| *Acontias meleagris* |  | ORJansenvil171 | Jansenville, EC | -32.638333 | 24.685 |  |  |  | JQ692399 |  |  |
| *Acontias meleagris* |  | ORJansen172 |  |  |  |  |  |  |  |  | JQ278080 |
| *Acontias meleagris* |  | ORJansen176 |  |  |  |  |  |  |  |  | JQ278081 |
| *Acontias meleagris* |  | Jansenville180 | Jansenville, EC | -32.638333 | 24.685 |  |  |  | JQ692400 |  | JQ278082 |
| *Acontias meleagris* |  | OSKatberg161 | Katberg, EC | -32.151833 | 25.75 |  |  |  | JQ692401 |  |  |
| *Acontias meleagris* |  | OSKatberg162 | Katberg, EC | -32.151833 | 25.75 |  |  |  | JQ692402 |  | JQ278083 |
| *Acontias meleagris* |  | OSKatberg163 | Katberg, EC | -32.151833 | 25.75 |  |  |  | JQ692403 |  |  |
| *Acontias meleagris* |  | Klipheuwel2 | Klipheuwel, WC | -33.703611 | 18.695277 |  |  |  | JQ692405 |  | JQ278084 |
| *Acontias meleagris* |  | Macassar | Macassar, WC | -34.053886 | 18.765593 |  |  |  | JQ692406 |  | JQ278086 |
| *Acontias meleagris* |  | Middeldrift | Middeldrift, EC | -32.791892 | 26.993408 |  |  |  | JQ692407 |  | JQ278087 |
| *Acontias meleagris* |  | Montagu1 | Montagu, WC | -33.838333 | 20.033333 |  |  |  | JQ692408 |  |  |
| *Acontias meleagris* |  | Montagu2 | Montagu, WC | -33.838333 | 20.033333 |  |  |  | JQ692409 |  | JQ278088 |
| *Acontias meleagris* |  | Montagu3 | Montagu, WC | -33.838333 | 20.033333 |  |  |  | JQ692410 |  | JQ278089 |
| *Acontias meleagris* |  | Montagu4 | Montagu, WC | -33.838333 | 20.033333 |  |  |  | JQ692411 |  | JQ278090 |
| *Acontias meleagris* |  | Muizenberg | Muizenberg, WC | -34.091336 | 18.480549 |  |  |  | JQ692412 |  |  |
| *Acontias meleagris* |  | Niewoudt253 | Niewoudtville, NC | -31.3241 | 19.11482 |  |  |  | JQ692413 |  | JQ278093 |
| *Acontias meleagris* |  | Niewoudt254 | Niewoudtville, NC | -31.3241 | 19.11482 |  |  |  | JQ692414 |  | JQ278091 |
| *Acontias meleagris* |  | Paterson1 | Paterson, EC | -33.365 | 26.502166 |  |  |  | JQ692415 |  |  |
| *Acontias meleagris* |  | Paterson2 | Paterson, EC | -33.365 | 26.502166 |  |  |  | JQ692416 |  |  |
| *Acontias meleagris* |  | Paterson3 | Paterson, EC | -33.365 | 26.502166 |  |  |  | JQ692417 |  | JQ278094 |
| *Acontias meleagris* |  | Paterson4 | Paterson, EC | -33.365 | 26.502166 |  |  |  | JQ692418 |  | JQ278095 |
| *Acontias meleagris* |  | ORTPearson89 | Pearston, EC | -32.586666 | 25.135833 |  |  |  | JQ692419 |  | JQ278096 |
| *Acontias meleagris* |  | Pearston90 |  |  |  |  |  |  |  |  | JQ278097 |
| *Acontias meleagris* |  | Pearston91 | Pearston, EC | -32.586666 | 25.135833 | // |  |  | JQ692420 |  | JQ278098 |
| *Acontias meleagris* |  | Pearston92 | Pearston, EC | -32.586666 | 25.135833 |  |  |  | JQ692421 |  | JQ278100 |
| *Acontias meleagris* |  | Pearston93 |  |  |  |  |  |  |  |  | JQ278099 |
| *Acontias meleagris* |  | Pearston94 | Pearston, EC | -32.586666 | 25.135833 |  |  |  | JQ692422 |  |  |
| *Acontias meleagris* |  | PEMR17 | Oyster Bay, EC | -34.090277 | 24.902222 |  |  |  | JQ692423 |  |  |
| *Acontias meleagris* |  | PringleBay | Pringle Bay, WC | -34.347617 | 18.831511 |  |  |  | JQ692424 |  | JQ278101 |
| *Acontias meleagris* |  | PringleBay2 | Pringle Bay, WC | -34.347617 | 18.831511 |  |  |  | JQ692425 |  | JQ278102 |
| *Acontias meleagris* |  | Qumbu2 | Qumbu, EC | -31.162106 | 28.869554 |  |  |  | JQ692426 |  | JQ278103 |
| *Acontias meleagris* |  | Qumbu3 | Qumbu, EC | -31.162106 | 28.869554 |  |  |  | JQ692427 |  |  |
| *Acontias meleagris* |  | Rawsonville1 | Rawsonville, WC | -33.666666 | 19.268333 |  |  |  | JQ692428 |  | JQ278104 |
| *Acontias meleagris* |  | Rawsonville2 | Rawsonville, WC | -33.666666 | 19.268333 |  |  |  | JQ692429 |  | JQ278105 |
| *Acontias meleagris* |  | Rawsonville3 | Rawsonville, WC | -33.666666 | 19.268333 |  |  |  | JQ692430 |  | JQ278106 |
| *Acontias meleagris* |  | Salem1 | Salem, EC | -32.801833 | 26.758333 |  |  |  | JQ692431 |  |  |
| *Acontias meleagris* |  | Salem2 | Salem, EC | -32.801833 | 26.758333 |  |  |  | JQ692432 |  | JQ278108 |
| *Acontias meleagris* |  | Salem3 | Salem, EC | -32.801833 | 26.758333 |  |  |  | JQ692433 |  |  |
| *Acontias meleagris* |  | Salem4 | Salem, EC | -32.801833 | 26.758333 |  |  |  | JQ692434 |  |  |
| *Acontias meleagris* |  | Salem5 | Salem, EC | -32.801833 | 26.758333 |  |  |  | JQ692435 |  |  |
| *Acontias meleagris* |  | Salem6 | Salem, EC | -32.801833 | 26.758333 |  |  |  | JQ692436 |  |  |
| *Acontias meleagris* |  | Salem7 | Salem, EC | -32.801833 | 26.758333 |  |  |  | JQ692437 |  |  |
| *Acontias meleagris* |  | Struisbaai230 | Struis Bay, WC | -34.666666 | 20.216666 |  |  |  | JQ692438 |  |  |
| *Acontias meleagris* |  | ORStruisbaai231 | Struis Bay, WC | -34.666666 | 20.216666 |  |  |  | JQ692439 |  | JQ278109 |
| *Acontias meleagris* |  | ORStruisbaai232 | Struis Bay, WC | -34.666666 | 20.216666 |  |  |  | JQ692440 |  |  |
| *Acontias meleagris* |  | Struisbaai235 |  |  |  |  |  |  |  |  | JQ278110 |
| *Acontias meleagris* |  | Struisbaai237 | Struis Bay, WC | -34.666666 | 20.216666 |  |  |  | JQ692441 |  |  |
| *Acontias meleagris* |  | Struisbaai238 | Struis Bay, WC | -34.666666 | 20.216666 |  |  |  | JQ692442 |  |  |
| *Acontias meleagris* |  | Struisbaai239 | Struis Bay, WC | -34.666666 | 20.216666 |  |  |  | JQ692443 |  |  |
| *Acontias meleagris* |  | Swellendam | Swellendam, WC | -34.008843 | 20.443153 |  |  |  | JQ692444 |  | JQ278111 |
| *Acontias meleagris* |  | Tandjies | Tandjiesberg, EC | -32.369523 | 24.682846 |  |  |  | JQ692445 |  |  |
| *Acontias meleagris* |  | Tarka | Tarkastad, EC | -32.016666 | 26.25 |  |  |  | JQ692446 |  |  |
| *Acontias meleagris* |  | ORTarka147 | Tarkastad, EC | -32.016666 | 26.25 |  |  |  | JQ692447 |  |  |
| *Acontias meleagris* |  | ORTarka148 |  |  |  |  |  |  |  |  | JQ278112 |
| *Acontias meleagris* |  | ORTarka149 | Tarkastad, EC | -32.016666 | 26.25 |  |  |  | JQ692448 |  |  |
| *Acontias meleagris* |  | ORTarka150 | Tarkastad, EC | -32.016666 | 26.25 |  |  |  | JQ692449 |  |  |
| *Acontius breviceps** |  | A.gracilicauda |  |  |  |  |  |  | AY683736 |  | JQ278035 |
| *Acontias percivali occidentalis** | CAS209634 | A.p.occiden |  |  |  |  |  |  | HQ180061 |  | JQ278038 |
| *Acontias litoralis** |  | A.litoralis |  |  |  |  |  |  | AY028868 |  | JQ278036 |
| *Potamonautes brincki + Potamonautes tuerkayi + Potamonautes parvicorpus* | | | | | | | | | |  |  |
| *Potamonautes brincki* |  | BettiesBay1 | Betties Bay, WC | -34.34215 | 18.92215 |  |  |  | KU561495 |  |  |
| *Potamonautes brincki* |  | PringleBay1 | Pringle Bay, WC | -34.3238 | 18.8383 |  |  |  | KU561496 |  |  |
| *Potamonautes brincki* |  | PringleBay2 | Pringle Bay, WC | -34.3238 | 18.8383 |  |  |  | KU561497 |  |  |
| *Potamonautes brincki* |  | PringleBay3 | Pringle Bay, WC | -34.3238 | 18.8383 |  |  |  | KU561498 |  |  |
| *Potamonautes brincki* |  | PringleBay4 | Pringle Bay, WC | -34.3238 | 18.8383 |  |  |  | KU561499 |  |  |
| *Potamonautes brincki* |  | PringleBay5 | Pringle Bay, WC | -34.3238 | 18.8383 |  |  |  | KU561500 |  |  |
| *Potamonautes tuerkayi* |  | Fernkloof1 | Fernklof NR, WC | -34.3895 | 19.27251667 |  |  |  | KU561467 |  |  |
| *Potamonautes tuerkayi* |  | Fernkloof2 | Fernklof NR, WC | -34.3895 | 19.27251667 |  |  |  | KU561468 |  |  |
| *Potamonautes tuerkayi* |  | Fernkloof4 | Fernklof NR, WC | -34.3895 | 19.27251667 |  |  |  | KU561469 |  |  |
| *Potamonautes tuerkayi* |  | Fernkloof5 | Fernklof NR, WC | -34.3895 | 19.27251667 |  |  |  | KU561470 |  |  |
| *Potamonautes tuerkayi* |  | Fernkloof6 | Fernklof NR, WC | -34.3895 | 19.27251667 |  |  |  | KU561471 |  |  |
| *Potamonautes tuerkayi* |  | Fernkloof7 | Fernklof NR, WC | -34.3895 | 19.27251667 |  |  |  | KU561472 |  |  |
| *Potamonautes tuerkayi* |  | Fernkloof8 | Fernklof NR, WC | -34.3895 | 19.27251667 |  |  |  | KU561473 |  |  |
| *Potamonautes tuerkayi* |  | Fernkloof9 | Fernklof NR, WC | -34.3895 | 19.27251667 |  |  |  | KU561474 |  |  |
| *Potamonautes tuerkayi* |  | Fernkloof10 | Fernklof NR, WC | -34.3895 | 19.27251667 |  |  |  | KU561475 |  |  |
| *Potamonautes parvicorpus* |  | Jonkershoek1 | Jonkershoek NR, WC | -33.97123333 | 18.93823333 |  |  |  | KU561512 |  |  |
| *Potamonautes parvicorpus* |  | Jonkershoek2 | Jonkershoek NR, WC | -33.97123333 | 18.93823333 |  |  |  | KU561513 |  |  |
| *Potamonautes parvicorpus* |  | Jonkershoek3 | Jonkershoek NR, WC | -33.97123333 | 18.93823333 |  |  |  | KU561514 |  |  |
| *Potamonautes parvicorpus* |  | Jonkershoek4 | Jonkershoek NR, WC | -33.97123333 | 18.93823333 |  |  |  | KU561515 |  |  |
| *Potamonautes parvicorpus* |  | Jonkershoek5 | Jonkershoek NR, WC | -33.97123333 | 18.93823333 |  |  |  | KU561516 |  |  |
| *Potamonautes parvicorpus* |  | Jonkershoek6 | Jonkershoek NR, WC | -33.97123333 | 18.93823333 |  |  |  | KU561517 |  |  |
| *Potamonautes parvicorpus* |  | Kirstenbosch3 | Kirstenbosch NR, WC | -34.00091667 | 18.3883 |  |  |  | KU561518 |  |  |
| *Potamonautes parvicorpus* |  | Kirstenbosch2 | Kirstenbosch NR, WC | -34.00091667 | 18.3883 |  |  |  | KU561519 |  |  |
| *Potamonautes parvicorpus* |  | Kirstenbosch4 | Kirstenbosch NR, WC | -34.00091667 | 18.3883 |  |  |  | KU561520 |  |  |
| *Potamonautes tuerkayi* |  | Napier1 | Napier, WC | -34.42595 | 19.9069 |  |  |  | KU561476 |  |  |
| *Potamonautes tuerkayi* |  | Napier2 | Napier, WC | -34.42595 | 19.9069 |  |  |  | KU561477 |  |  |
| *Potamonautes tuerkayi* |  | Napier3 | Napier, WC | -34.42595 | 19.9069 |  |  |  | KU561478 |  |  |
| *Potamonautes tuerkayi* |  | Napier4 | Napier, WC | -34.42595 | 19.9069 |  |  |  | KU561479 |  |  |
| *Potamonautes tuerkayi* |  | Napier5 | Napier, WC | -34.42595 | 19.9069 |  |  |  | KU561480 |  |  |
| *Potamonautes tuerkayi* |  | Napier6 | Napier, WC | -34.42595 | 19.9069 |  |  |  | KU561481 |  |  |
| *Potamonautes tuerkayi* |  | Napier7 | Napier, WC | -34.42595 | 19.9069 |  |  |  | KU561482 |  |  |
| *Potamonautes tuerkayi* |  | Napier8 | Napier, WC | -34.42595 | 19.9069 |  |  |  | KU561483 |  |  |
| *Potamonautes tuerkayi* |  | Napier9 | Napier, WC | -34.42595 | 19.9069 |  |  |  | KU561484 |  |  |
| *Potamonautes tuerkayi* |  | Napier10 | Napier, WC | -34.42595 | 19.9069 |  |  |  | KU561485 |  |  |
| *Potamonautes brincki* |  | Rooiels2 | Rooiels, WC | -34.29271667 | 18.86708333 |  |  |  | KU561486 |  |  |
| *Potamonautes brincki* |  | Rooiels4 | Rooiels, WC | -34.29271667 | 18.86708333 |  |  |  | KU561487 |  |  |
| *Potamonautes brincki* |  | Rooiels5 | Rooiels, WC | -34.29271667 | 18.86708333 |  |  |  | KU561488 |  |  |
| *Potamonautes parvicorpus* |  | OrangeKloof1 | Orangekloof, WC | -33.99316667 | 18.38851667 |  |  |  | KU561521 |  |  |
| *Potamonautes parvicorpus* |  | OrangeKloof2 | Orangekloof, WC | -33.99316667 | 18.38851667 |  |  |  | KU561522 |  |  |
| *Potamonautes parvicorpus* |  | OrangeKloof3 | Orangekloof, WC | -33.99316667 | 18.38851667 |  |  |  | KU561523 |  |  |
| *Potamonautes parvicorpus* |  | OrangeKloof4 | Orangekloof, WC | -33.99316667 | 18.38851667 |  |  |  | KU561524 |  |  |
| *Potamonautes parvicorpus* |  | OrangeKloof5 | Orangekloof, WC | -33.99316667 | 18.38851667 |  |  |  | KU561525 |  |  |
| *Potamonautes parvicorpus* |  | OrangeKloof6 | Orangekloof, WC | -33.99316667 | 18.38851667 |  |  |  | KU561526 |  |  |
| *Potamonautes parvicorpus* |  | OrangeKloof7 | Orangekloof, WC | -33.99316667 | 18.38851667 |  |  |  | KU561527 |  |  |
| *Potamonautes parvicorpus* |  | OrangeKloof8 | Orangekloof, WC | -33.99316667 | 18.38851667 |  |  |  | KU561528 |  |  |
| *Potamonautes parvicorpus* |  | OrangeKloof9 | Orangekloof, WC | -33.99316667 | 18.38851667 |  |  |  | KU561529 |  |  |
| *Potamonautes brincki* |  | Palmiet1 | Palmiet, WC | -34.27021667 | 19.00958333 |  |  |  | KU561489 |  |  |
| *Potamonautes brincki* |  | Palmiet2 | Palmiet, WC | -34.27021667 | 19.00958333 |  |  |  | KU561490 |  |  |
| *Potamonautes brincki* |  | Palmiet3 | Palmiet, WC | -34.27021667 | 19.00958333 |  |  |  | KU561491 |  |  |
| *Potamonautes brincki* |  | Palmiet4 | Palmiet, WC | -34.27021667 | 19.00958333 |  |  |  | KU561492 |  |  |
| *Potamonautes brincki* |  | Palmiet5 | Palmiet, WC | -34.27021667 | 19.00958333 |  |  |  | KU561493 |  |  |
| *Potamonautes brincki* |  | Palmiet6 | Palmiet, WC | -34.27021667 | 19.00958333 |  |  |  | KU561494 |  |  |
| *Potamonautes parvicorpus* |  | Platteklip1 | Platteklip Gorge, WC | -33.95573333 | 18.40783333 |  |  |  | KU561530 |  |  |
| *Potamonautes parvicorpus* |  | Platteklip2 | Platteklip Gorge, WC | -33.95573333 | 18.40783333 |  |  |  | KU561531 |  |  |
| *Potamonautes parvicorpus* |  | Platteklip3 | Platteklip Gorge, WC | -33.95573333 | 18.40783333 |  |  |  | KU561532 |  |  |
| *Potamonautes parvicorpus* |  | Platteklip4 | Platteklip Gorge, WC | -33.95573333 | 18.40783333 |  |  |  | KU561533 |  |  |
| *Potamonautes parvicorpus* |  | Platteklip5 | Platteklip Gorge, WC | -33.95573333 | 18.40783333 |  |  |  | KU561534 |  |  |
| *Potamonautes parvicorpus* |  | Platteklip6 | Platteklip Gorge, WC | -33.95573333 | 18.40783333 |  |  |  | KU561535 |  |  |
| *Potamonautes parvicorpus* |  | Platteklip7 | Platteklip Gorge, WC | -33.95573333 | 18.40783333 |  |  |  | KU561536 |  |  |
| *Potamonautes parvicorpus* |  | Platteklip8 | Platteklip Gorge, WC | -33.95573333 | 18.40783333 |  |  |  | KU561537 |  |  |
| *Potamonautes parvicorpus* |  | SirLouwries1 | Sir Lowries Pass, WC | -34.1526 | 18.93725 |  |  |  | KU561501 |  |  |
| *Potamonautes parvicorpus* |  | SirLouwries2 | Sir Lowries Pass, WC | -34.1526 | 18.93725 |  |  |  | KU561502 |  |  |
| *Potamonautes parvicorpus* |  | SirLouwries3 | Sir Lowries Pass, WC | -34.1526 | 18.93725 |  |  |  | KU561503 |  |  |
| *Potamonautes parvicorpus* |  | SirLouwries4 | Sir Lowries Pass, WC | -34.1526 | 18.93725 |  |  |  | KU561504 |  |  |
| *Potamonautes parvicorpus* |  | SirLouwries5 | Sir Lowries Pass, WC | -34.1526 | 18.93725 |  |  |  | KU561505 |  |  |
| *Potamonautes parvicorpus* |  | SirLouwries6 | Sir Lowries Pass, WC | -34.1526 | 18.93725 |  |  |  | KU561506 |  |  |
| *Potamonautes brincki* |  | Steenbras1 | Steenbras dam, WC | -34.19383333 | 18.8244 |  |  |  | KU561507 |  |  |
| *Potamonautes brincki* |  | Steenbras2 | Steenbras dam, WC | -34.19383333 | 18.8244 |  |  |  | KU561508 |  |  |
| *Potamonautes brincki* |  | Steenbras3 | Steenbras dam, WC | -34.19383333 | 18.8244 |  |  |  | KU561509 |  |  |
| *Potamonautes brincki* |  | Steenbras4 | Steenbras dam, WC | -34.19383333 | 18.8244 |  |  |  | KU561510 |  |  |
| *Potamonautes brincki* |  | Steenbras5 | Steenbras dam, WC | -34.19383333 | 18.8244 |  |  |  | KU561511 |  |  |
| *Potamonautes parvicorpus* |  | Blinkwater1 | Blinkwater, WC | -34.10018333 | 18.81948333 |  |  |  | KU561538 |  |  |
| *Potamonautes parvicorpus* |  | Blinkwater2 | Blinkwater, WC | -34.10018333 | 18.81948333 |  |  |  | KU561539 |  |  |
| *Potamonautes parvicorpus* |  | Blinkwater3 | Blinkwater, WC | -34.10018333 | 18.81948333 |  |  |  | KU561540 |  |  |
| *Potamonautes parvicorpus* |  | Blinkwater4 | Blinkwater, WC | -34.10018333 | 18.81948333 |  |  |  | KU561541 |  |  |
| *Potamonautes parvicorpus* |  | Silvermine1 | Silvermine NR, WC | -33.95865 | 18.65633333 |  |  |  | KU561542 |  |  |
| *Potamonautes depressus** |  | P.depressus |  |  |  |  |  |  | KU157058 |  |  |
| *Potamonautes clarus** |  | P.clarus |  |  |  |  |  |  | KU157043 |  |  |
| *Potamonautes perlatus + Potamonautes barbarai + Potamonautes barnardi* | | | | | | | | | | | |
| *Potamonautes barbarai* |  | Andrieskr1 | Andrieskraal, EC | -33.742831 | 24.629981 |  |  | DQ028650 |  |  |  |
| *Potamonautes barbarai* |  | Andrieskr2 | Andrieskraal, EC | -33.742831 | 24.629981 |  |  | DQ028651 |  |  |  |
| *Potamonautes barbarai* |  | Andrieskr3 | Andrieskraal, EC | -33.742831 | 24.629981 |  |  | DQ028652 |  |  |  |
| *Potamonautes barbarai* |  | Andrieskr4 | Andrieskraal, EC | -33.742831 | 24.629981 |  |  | DQ028653 |  |  |  |
| *Potamonautes barbarai* |  | Andrieskr5 | Andrieskraal, EC | -33.742831 | 24.629981 |  |  | DQ028654 |  |  |  |
| *Potamonautes barnardi* |  | Bainsklf1 | Bainskloof, WC | -33.589581 | 19.129067 |  |  | AF493173 | AF494031 |  |  |
| *Potamonautes barnardi* |  | Bainsklf2 | Bainskloof, WC | -33.589581 | 19.129067 |  |  | AF493174 |  |  |  |
| *Potamonautes barnardi* |  | Bainsklf3 | Bainskloof, WC | -33.589581 | 19.129067 |  |  | AF493176 | AF494030 |  |  |
| *Potamonautes perlatus* |  | Boesman1 | Boesmansbos, WC | -32.515286 | 19.376333 |  |  |  |  |  |  |
| *Potamonautes perlatus* |  | Boesman2 | Boesmansbos, WC | -32.515286 | 19.376333 |  |  |  |  |  |  |
| *Potamonautes perlatus* |  | Boesman3 | Boesmansbos, WC | -32.515286 | 19.376333 |  |  |  |  |  |  |
| *Potamonautes barnardi* |  | Bonnievale | Bonnievale, WC | -33.945622 | 20.102786 |  |  |  | AF494032 |  |  |
| *Potamonautes perlatus* |  | Boontjies1 | Boontjieskloof, WC | -32.558064 | 19.113869 |  |  | DQ028676 |  |  |  |
| *Potamonautes perlatus* |  | Boontjies2 | Boontjieskloof, WC | -32.558064 | 19.113869 |  |  | DQ028675 |  |  |  |
| *Potamonautes barbarai* |  | Bosdorp1 | Bosdorp, EC | -33.669383 | 24.574017 |  |  | DQ028660 |  |  |  |
| *Potamonautes barbarai* |  | Bosdorp2 | Bosdorp, EC | -33.669383 | 24.574017 |  |  | DQ028661 |  |  |  |
| *Potamonautes barbarai* |  | Bosdorp3 | Bosdorp, EC | -33.669383 | 24.574017 |  |  | DQ028662 |  |  |  |
| *Potamonautes barbarai* |  | Bosdorp4 | Bosdorp, EC | -33.669383 | 24.574017 |  |  | DQ028663 |  |  |  |
| *Potamonautes barbarai* |  | Bosdorp5 | Bosdorp, EC | -33.669383 | 24.574017 |  |  | DQ028664 |  |  |  |
| *Potamonautes perlatus* |  | Citrusdal1 | Citrusdal, WC | -32.562367 | 19.318369 |  |  |  | AF494022 |  |  |
| *Potamonautes perlatus* |  | Citrusdal2 | Citrusdal, WC | -32.562367 | 19.318369 |  |  | AF493162 |  |  |  |
| *Potamonautes perlatus* |  | Citrusdal3 | Citrusdal, WC | -32.562367 | 19.318369 |  |  |  |  |  |  |
| *Potamonautes perlatus* |  | Citrusdal4 | Citrusdal, WC | -32.562367 | 19.318369 |  |  |  |  |  |  |
| *Potamonautes perlatus* |  | Clanwill1 | Clanwilliam, WC | -32.208492 | 18.969386 |  |  |  |  |  |  |
| *Potamonautes perlatus* |  | Clanwill2 | Clanwilliam, WC | -32.208492 | 18.969386 |  |  |  |  |  |  |
| *Potamonautes barnardi* |  | DeHoop1 | De Hoop, WC | -34.350272 | 20.596106 |  |  | AF493166 | AF494035 |  |  |
| *Potamonautes barnardi* |  | DeHoop2 | De Hoop, WC | -34.350272 | 20.596106 |  |  |  |  |  |  |
| *Potamonautes barnardi* |  | DeHoop3 | De Hoop, WC | -34.350272 | 20.596106 |  |  | AF493167 |  |  |  |
| *Potamonautes barnardi* |  | DeHoop4 | De Hoop, WC | -34.350272 | 20.596106 |  |  | AF493168 | AF494036 |  |  |
| *Potamonautes barbarai* |  | Dwyka1 | Dwyka River, WC | -33.08735 | 21.570931 |  |  | DQ028732 |  |  |  |
| *Potamonautes barbarai* |  | Dwyka2 | Dwyka River, WC | -33.08735 | 21.570931 |  |  | DQ028733 |  |  |  |
| *Potamonautes barbarai* |  | Dwyka3 | Dwyka River, WC | -33.08735 | 21.570931 |  |  | DQ028731 |  |  |  |
| *Potamonautes barbarai* |  | Dwyka4 | Dwyka River, WC | -33.08735 | 21.570931 |  |  | DQ028730 |  |  |  |
| *Potamonautes barbarai* |  | Dwyka5 | Dwyka River, WC | -33.08735 | 21.570931 |  |  | DQ028734 |  |  |  |
| *Potamonautes barbarai* |  | Groot1 | Groot River, WC | -33.670611 | 21.165992 |  |  | DQ028686 |  |  |  |
| *Potamonautes barbarai* |  | Groot2 | Groot River, WC | -33.670611 | 21.165992 |  |  | DQ028684 |  |  |  |
| *Potamonautes barbarai* |  | Groot3 | Groot River, WC | -33.670611 | 21.165992 |  |  | DQ028682 |  |  |  |
| *Potamonautes barbarai* |  | Groot4 | Groot River, WC | -33.670611 | 21.165992 |  |  | DQ028685 |  |  |  |
| *Potamonautes barbarai* |  | Groot5 | Groot River, WC | -33.670611 | 21.165992 |  |  | DQ028683 |  |  |  |
| *Potamonautes barbarai* |  | Hankey1 | Hankey, EC | -33.841831 | 24.861986 |  |  | DQ028640 |  |  |  |
| *Potamonautes barbarai* |  | Hankey2 | Hankey, EC | -33.841831 | 24.861986 |  |  | DQ028641 |  |  |  |
| *Potamonautes barbarai* |  | Hankey3 | Hankey, EC | -33.841831 | 24.861986 |  |  | DQ028642 |  |  |  |
| *Potamonautes barbarai* |  | Hankey4 | Hankey, EC | -33.841831 | 24.861986 |  |  | DQ028643 |  |  |  |
| *Potamonautes barbarai* |  | Huisriver1 | Huis River, WC | -33.500542 | 21.600689 |  |  | DQ028690 |  |  |  |
| *Potamonautes barbarai* |  | Huisriver2 | Huis River, WC | -33.500542 | 21.600689 |  |  | DQ028691 |  |  |  |
| *Potamonautes barbarai* |  | Huisriver3 | Huis River, WC | -33.500542 | 21.600689 |  |  | DQ028688 |  |  |  |
| *Potamonautes barbarai* |  | Huisriver4 | Huis River, WC | -33.500542 | 21.600689 |  |  | DQ028689 |  |  |  |
| *Potamonautes barbarai* |  | Huisriver5 | Huis River, WC | -33.500542 | 21.600689 |  |  | DQ028687 |  |  |  |
| *Potamonautes barbarai* |  | Kleinplts1 | Kleinplaats, EC | -33.636917 | 24.453944 |  |  |  |  |  |  |
| *Potamonautes barbarai* |  | Kleinplts2 | Kleinplaats, EC | -33.636917 | 24.453944 |  |  |  |  |  |  |
| *Potamonautes barbarai* |  | Kleinplts3 | Kleinplaats, EC | -33.636917 | 24.453944 |  |  | AF493164 |  |  |  |
| *Potamonautes barbarai* |  | Kleinplts4 | Kleinplaats, EC | -33.636917 | 24.453944 |  |  |  |  |  |  |
| *Potamonautes barbarai* |  | Kleinplts5 | Kleinplaats, EC | -33.636917 | 24.453944 |  |  |  |  |  |  |
| *Potamonautes barnardi* |  | Kleinrvr1 | Klein River, WC | -34.426492 | 19.511075 |  |  | DQ028709 |  |  |  |
| *Potamonautes perlatus* |  | Kriedouw1 | Kriedouwkrans, WC | -32.364736 | 18.954033 |  |  | DQ028677 |  |  |  |
| *Potamonautes perlatus* |  | Kriedouw2 | Kriedouwkrans, WC | -32.364736 | 18.954033 |  |  | DQ028678 |  |  |  |
| *Potamonautes perlatus* |  | Kriedouw3 | Kriedouwkrans, WC | -32.364736 | 18.954033 |  |  | DQ028679 |  |  |  |
| *Potamonautes perlatus* |  | Liesbeeck1 | Liesbeeck, WC | -34.190519 | 18.416639 |  |  | DQ028665 |  |  |  |
| *Potamonautes perlatus* |  | Liesbeeck2 | Liesbeeck, WC | -34.190519 | 18.416639 |  |  | DQ028666 |  |  |  |
| *Potamonautes perlatus* |  | Liesbeeck3 | Liesbeeck, WC | -34.190519 | 18.416639 |  |  | DQ028667 |  |  |  |
| *Potamonautes perlatus* |  | Liesbeeck4 | Liesbeeck, WC | -34.190519 | 18.416639 |  |  | DQ028668 |  |  |  |
| *Potamonautes barbarai* |  | Nels1 | Nels River, WC | -33.487644 | 21.433878 |  |  | DQ028696 |  |  |  |
| *Potamonautes barbarai* |  | Nels2 | Nels River, WC | -33.487644 | 21.433878 |  |  | DQ028698 |  |  |  |
| *Potamonautes barbarai* |  | Nels3 | Nels River, WC | -33.487644 | 21.433878 |  |  | DQ028700 |  |  |  |
| *Potamonautes barbarai* |  | Nels4 | Nels River, WC | -33.487644 | 21.433878 |  |  | DQ028697 |  |  |  |
| *Potamonautes barbarai* |  | Nels5 | Nels River, WC | -33.487644 | 21.433878 |  |  | DQ028699 |  |  |  |
| *Potamonautes perlatus* |  | Olifants1 | Olifant River, WC | -31.901364 | 18.604317 |  |  | AF493160 |  |  |  |
| *Potamonautes perlatus* |  | Paarl1 | Paarl, WC | -33.829569 | 19.054844 |  |  |  |  |  |  |
| *Potamonautes perlatus* |  | Paarl2 | Paarl, WC | -33.829569 | 19.054844 |  |  |  |  |  |  |
| *Potamonautes perlatus* |  | Paarl3 | Paarl, WC | -33.829569 | 19.054844 |  |  | AF493163 |  |  |  |
| *Potamonautes perlatus* |  | Paarl9 | Paarl, WC | -33.829569 | 19.054844 |  |  |  |  |  |  |
| *Potamonautes perlatus* |  | Paarl10 | Paarl, WC | -33.829569 | 19.054844 |  |  |  |  |  |  |
| *Potamonautes barbarai* |  | Patensie1 | Patensie, EC | -33.776225 | 24.809872 |  |  | DQ028645 |  |  |  |
| *Potamonautes barbarai* |  | Patensie2 | Patensie, EC | -33.776225 | 24.809872 |  |  | DQ028646 |  |  |  |
| *Potamonautes barbarai* |  | Patensie3 | Patensie, EC | -33.776225 | 24.809872 |  |  | DQ028647 |  |  |  |
| *Potamonautes barbarai* |  | Patensie4 | Patensie, EC | -33.776225 | 24.809872 |  |  | DQ028648 |  |  |  |
| *Potamonautes barbarai* |  | Poortjies1 | Poortjies, EC | -33.658481 | 24.531992 |  |  | AF493165 | AF494023 |  |  |
| *Potamonautes barbarai* |  | Poortjies2 | Poortjies, EC | -33.658481 | 24.531992 |  |  |  |  |  |  |
| *Potamonautes barbarai* |  | Poortjies3 | Poortjies, EC | -33.658481 | 24.531992 |  |  |  |  |  |  |
| *Potamonautes barbarai* |  | PrinceAlb1 | Prince Albert, WC | -33.174269 | 22.027631 |  |  | DQ028719 |  |  |  |
| *Potamonautes barbarai* |  | PrinceAlb2 | Prince Albert, WC | -33.174269 | 22.027631 |  |  | DQ028717 |  |  |  |
| *Potamonautes barbarai* |  | PrinceAlb3 | Prince Albert, WC | -33.174269 | 22.027631 |  |  | DQ028715 |  |  |  |
| *Potamonautes barbarai* |  | PrinceAlb4 | Prince Albert, WC | -33.174269 | 22.027631 |  |  | DQ028718 |  |  |  |
| *Potamonautes barbarai* |  | PrinceAlb5 | Prince Albert, WC | -33.174269 | 22.027631 |  |  | DQ028716 |  |  |  |
| *Potamonautes barnardi* |  | Robertson1 | Robertson, WC | -33.832397 | 19.869556 |  |  | AF493170 | AF494026 |  |  |
| *Potamonautes barnardi* |  | Robertson2 | Robertson, WC | -33.832397 | 19.869556 |  |  |  |  |  |  |
| *Potamonautes barnardi* |  | Robertson3 | Robertson, WC | -33.832397 | 19.869556 |  |  | AF493171 | AF494028 |  |  |
| *Potamonautes barnardi* |  | Robertson4 | Robertson, WC | -33.832397 | 19.869556 |  |  | AF493169 | AF494027 |  |  |
| *Potamonautes barbarai* |  | Sandriver1 | Sand River, EC | -33.689811 | 24.591153 |  |  | DQ028635 |  |  |  |
| *Potamonautes barbarai* |  | Sandriver2 | Sand River, EC | -33.689811 | 24.591153 |  |  | DQ028636 |  |  |  |
| *Potamonautes barbarai* |  | Sandriver3 | Sand River, EC | -33.689811 | 24.591153 |  |  | DQ028637 |  |  |  |
| *Potamonautes barbarai* |  | Sandriver4 | Sand River, EC | -33.689811 | 24.591153 |  |  | DQ028638 |  |  |  |
| *Potamonautes barbarai* |  | Smithskrl1 | Smitskraal, EC | -33.653444 | 24.354361 |  |  | DQ028655 |  |  |  |
| *Potamonautes barbarai* |  | Smithskrl2 | Smitskraal, EC | -33.653444 | 24.354361 |  |  | DQ028656 |  |  |  |
| *Potamonautes barbarai* |  | Smithskrl3 | Smitskraal, EC | -33.653444 | 24.354361 |  |  | DQ028657 |  |  |  |
| *Potamonautes barbarai* |  | Smithskrl4 | Smitskraal, EC | -33.653444 | 24.354361 |  |  | DQ028658 |  |  |  |
| *Potamonautes barbarai* |  | Smithskrl5 | Smitskraal, EC | -33.653444 | 24.354361 |  |  | DQ028659 |  |  |  |
| *Potamonautes perlatus* |  | Stellnbs1 | Stellenbosch, WC | -33.95183 | 18.90585 |  |  | DQ028670 |  |  |  |
| *Potamonautes perlatus* |  | Stellnbs2 | Stellenbosch, WC | -33.95183 | 18.90585 |  |  | DQ028671 |  |  |  |
| *Potamonautes perlatus* |  | Stellnbs3 | Stellenbosch, WC | -33.95183 | 18.90585 |  |  | DQ028672 |  |  |  |
| *Potamonautes perlatus* |  | Stellnbs4 | Stellenbosch, WC | -33.95183 | 18.90585 |  |  | DQ028673 |  |  |  |
| *Potamonautes perlatus* |  | Stellnbs5 | Stellenbosch, WC | -33.95183 | 18.90585 |  |  | DQ028674 |  |  |  |
| *Potamonautes barbarai* |  | Swartberg1 | Swartberg, WC | -33.324906 | 22.047717 |  |  | DQ028693 |  |  |  |
| *Potamonautes barbarai* |  | Swartberg2 | Swartberg, WC | -33.324906 | 22.047717 |  |  | DQ028695 |  |  |  |
| *Potamonautes barbarai* |  | Swartberg3 | Swartberg, WC | -33.324906 | 22.047717 |  |  | DQ028694 |  |  |  |
| *Potamonautes barbarai* |  | Swartberg4 | Swartberg, WC | -33.324906 | 22.047717 |  |  | DQ028692 |  |  |  |
| *Potamonautes perlatus* |  | Tokai1 | Tokai, WC | -34 | 18.387022 |  |  |  | AF494024 |  |  |
| *Potamonautes perlatus* |  | Tokai2 | Tokai, WC | -34 | 18.387022 |  |  |  | AF494025 |  |  |
| *Potamonautes perlatus* |  | Tokai3 | Tokai, WC | -34 | 18.387022 |  |  | AF493172 |  |  |  |
| *Potamonautes perlatus* |  | Tokai4 | Tokai, WC | -34 | 18.387022 |  |  |  |  |  |  |
| *Potamonautes barbarai* |  | Touws1 | Touws River, WC | -33.339392 | 20.014 |  |  |  |  |  |  |
| *Potamonautes barnardi* |  | TunnelTer1 | Tunnel Terminal, WC | -33.730908 | 19.118247 |  |  |  |  |  |  |
| *Potamonautes barnardi* |  | TunnelTer2 | Tunnel Terminal, WC | -33.730908 | 19.118247 |  |  |  |  |  |  |
| *Potamonautes barnardi* |  | TunnelTer3 | Tunnel Terminal, WC | -33.730908 | 19.118247 |  |  |  |  |  |  |
| *Potamonautes barnardi* |  | TunnelTer4 | Tunnel Terminal, WC | -33.730908 | 19.118247 |  |  |  |  |  |  |
| *Potamonautes barbarai* |  | Vette1 | Vet River, WC | -34.019822 | 21.216314 |  |  | DQ028720 |  |  |  |
| *Potamonautes barbarai* |  | Vette2 | Vet River, WC | -34.019822 | 21.216314 |  |  | DQ028722 |  |  |  |
| *Potamonautes barbarai* |  | Vette3 | Vet River, WC | -34.019822 | 21.216314 |  |  | DQ028723 |  |  |  |
| *Potamonautes barbarai* |  | Vette4 | Vet River, WC | -34.019822 | 21.216314 |  |  | DQ028724 |  |  |  |
| *Potamonautes barbarai* |  | Vette5 | Vet River, WC | -34.019822 | 21.216314 |  |  | DQ028721 |  |  |  |
| *Potamonautes barbarai* |  | Vleiriv1 | Vlei River, WC | -33.551833 | 21.884861 |  |  | DQ028712 |  |  |  |
| *Potamonautes barbarai* |  | Vleiriv2 | Vlei River, WC | -33.551833 | 21.884861 |  |  | DQ028711 |  |  |  |
| *Potamonautes barbarai* |  | Vleiriv3 | Vlei River, WC | -33.551833 | 21.884861 |  |  | DQ028713 |  |  |  |
| *Potamonautes barbarai* |  | Vleiriv4 | Vlei River, WC | -33.551833 | 21.884861 |  |  | DQ028714 |  |  |  |
| *Potamonautes perlatus* |  | Vlei5 | Vlei River, WC | -33.551833 | 21.884861 |  |  |  |  |  |  |
| *Potamonautes unispinus** |  | P.unispinus |  |  |  |  |  | AY042250 | AF510870 |  |  |
| *Potamonautes warreni** |  | P.warreni |  |  |  |  |  | AY042251 | AF510880 |  |  |
| *Potamonautes sidneyi** |  | P.sidneyi |  |  |  |  |  | AY042245 | AF510871 |  |  |
| *Peripatopsis capensis + Peripatopsis lawrencei + Peripatopsis overbergiensis* | | | | | | | | | | | |
| *Peripatopsis overbergiensis* |  | Font1 | Marloth Reserve, WC | -33.995833 | 20.4575 |  |  |  | KP640366 |  |  |
| *Peripatopsis overbergiensis* |  | Font2 | Marloth Reserve, WC | -33.995833 | 20.4575 |  |  |  | KP640366 |  |  |
| *Peripatopsis overbergiensis* |  | Font3 | Marloth Reserve, WC | -33.995833 | 20.4575 |  |  |  | KP640370 |  |  |
| *Peripatopsis overbergiensis* |  | Font4 | Marloth Reserve, WC | -33.995833 | 20.4575 |  |  |  | KP640366 |  |  |
| *Peripatopsis overbergiensis* |  | Font5 | Marloth Reserve, WC | -33.995833 | 20.4575 |  |  |  | KP640366 |  |  |
| *Peripatopsis overbergiensis* |  | Font6 | Marloth Reserve, WC | -33.995833 | 20.4575 |  |  |  | KP640366 |  |  |
| *Peripatopsis overbergiensis* |  | Font7 | Marloth Reserve, WC | -33.995833 | 20.4575 |  |  |  | KP640368 |  |  |
| *Peripatopsis overbergiensis* |  | Font8 | Marloth Reserve, WC | -33.995833 | 20.4575 |  |  |  | KP640369 |  |  |
| *Peripatopsis overbergiensis* |  | Font9 | Marloth Reserve, WC | -33.995833 | 20.4575 |  |  |  | KP640366 |  |  |
| *Peripatopsis overbergiensis* |  | Font10 | Marloth Reserve, WC | -33.995833 | 20.4575 |  |  |  | KP640366 |  |  |
| *Peripatopsis overbergiensis* |  | GVBA1 | Grootvadersbosch Reserve, WC | -33.984167 | 20.824167 |  |  |  | KP640366 |  |  |
| *Peripatopsis overbergiensis* |  | GVBA2 | Grootvadersbosch Reserve, WC | -33.984167 | 20.824167 |  |  |  | KP640366 |  |  |
| *Peripatopsis overbergiensis* |  | GVBA3 | Grootvadersbosch Reserve, WC | -33.984167 | 20.824167 |  |  |  | KP640366 |  |  |
| *Peripatopsis overbergiensis* |  | GVBA4 | Grootvadersbosch Reserve, WC | -33.984167 | 20.824167 |  |  |  | KP640368 |  |  |
| *Peripatopsis overbergiensis* |  | GVBA5 | Grootvadersbosch Reserve, WC | -33.984167 | 20.824167 |  |  |  | KP640366 |  |  |
| *Peripatopsis overbergiensis* |  | GVBA6 | Grootvadersbosch Reserve, WC | -33.984167 | 20.824167 |  |  |  | KP640366 |  |  |
| *Peripatopsis overbergiensis* |  | GVBA7 | Grootvadersbosch Reserve, WC | -33.984167 | 20.824167 |  |  |  | KP640369 |  |  |
| *Peripatopsis overbergiensis* |  | GVBA9 | Grootvadersbosch Reserve, WC | -33.984167 | 20.824167 |  |  |  | KP640366 |  |  |
| *Peripatopsis overbergiensis* |  | GVBA10 | Grootvadersbosch Reserve, WC | -33.984167 | 20.824167 |  |  |  | KP640366 |  |  |
| *Peripatopsis overbergiensis* |  | GVBB1 | Grootvadersbosch Reserve, WC | -33.984167 | 20.824444 |  |  |  | KP640366 |  |  |
| *Peripatopsis overbergiensis* |  | GVBB3 | Grootvadersbosch Reserve, WC | -33.984167 | 20.824444 |  |  |  | KP640367 |  |  |
| *Peripatopsis overbergiensis* |  | GVBB4 | Grootvadersbosch Reserve, WC | -33.984167 | 20.824444 |  |  |  | KP640366 |  |  |
| *Peripatopsis overbergiensis* |  | GVBB5 | Grootvadersbosch Reserve, WC | -33.984167 | 20.824444 |  |  |  | KP640366 |  |  |
| *Peripatopsis overbergiensis* |  | GVBB6 | Grootvadersbosch Reserve, WC | -33.984167 | 20.824444 |  |  |  | KP640366 |  |  |
| *Peripatopsis overbergiensis* |  | GVBB7 | Grootvadersbosch Reserve, WC | -33.984167 | 20.824444 |  |  |  | KP640366 |  |  |
| *Peripatopsis overbergiensis* |  | GVBB8 | Grootvadersbosch Reserve, WC | -33.984167 | 20.824444 |  |  |  | KP640366 |  |  |
| *Peripatopsis overbergiensis* |  | GVBB9 | Grootvadersbosch Reserve, WC | -33.984167 | 20.824444 |  |  |  | KP640370 |  |  |
| *Peripatopsis overbergiensis* |  | GVBB10 | Grootvadersbosch Reserve, WC | -33.984167 | 20.824444 |  |  |  | KP640366 |  |  |
| *Peripatopsis overbergiensis* |  | GVBC1 | Grootvadersbosch Reserve, WC | -33.9825 | 20.832222 |  |  |  | KP640366 |  |  |
| *Peripatopsis overbergiensis* |  | GVBC2 | Grootvadersbosch Reserve, WC | -33.9825 | 20.832222 |  |  |  | KP640366 |  |  |
| *Peripatopsis overbergiensis* |  | GVBC3 | Grootvadersbosch Reserve, WC | -33.9825 | 20.832222 |  |  |  | KP640366 |  |  |
| *Peripatopsis overbergiensis* |  | GVBD1 | Grootvadersbosch Reserve, WC | -33.984722 | 20.816111 |  |  |  | KP640366 |  |  |
| *Peripatopsis overbergiensis* |  | GVBD2 | Grootvadersbosch Reserve, WC | -33.984722 | 20.816111 |  |  |  | KP640366 |  |  |
| *Peripatopsis overbergiensis* |  | GVBD3 | Grootvadersbosch Reserve, WC | -33.984722 | 20.816111 |  |  |  | KP640366 |  |  |
| *Peripatopsis overbergiensis* |  | GVBD4 | Grootvadersbosch Reserve, WC | -33.984722 | 20.816111 |  |  |  | KP640366 |  |  |
| *Peripatopsis overbergiensis* |  | GVBD5 | Grootvadersbosch Reserve, WC | -33.984722 | 20.816111 |  |  |  | KP640366 |  |  |
| *Peripatopsis overbergiensis* |  | GVBD7 | Grootvadersbosch Reserve, WC | -33.984722 | 20.816111 |  |  |  | KP640366 |  |  |
| *Peripatopsis overbergiensis* |  | KLB1 | Marloth Reserve, WC | -33.995556 | 20.452222 |  |  |  | KP640381 |  |  |
| *Peripatopsis overbergiensis* |  | KLB2 | Marloth Reserve, WC | -33.995556 | 20.452222 |  |  |  | KP640375 |  |  |
| *Peripatopsis overbergiensis* |  | KLB3 | Marloth Reserve, WC | -33.995556 | 20.452222 |  |  |  | KP640379 |  |  |
| *Peripatopsis overbergiensis* |  | KLB4 | Marloth Reserve, WC | -33.995556 | 20.452222 |  |  |  | KP640381 |  |  |
| *Peripatopsis overbergiensis* |  | KLB5 | Marloth Reserve, WC | -33.995556 | 20.452222 |  |  |  | KP640377 |  |  |
| *Peripatopsis overbergiensis* |  | KLB6 | Marloth Reserve, WC | -33.995556 | 20.452222 |  |  |  | KP640373 |  |  |
| *Peripatopsis overbergiensis* |  | KLB7 | Marloth Reserve, WC | -33.995556 | 20.452222 |  |  |  | KP640376 |  |  |
| *Peripatopsis overbergiensis* |  | KLB8 | Marloth Reserve, WC | -33.995556 | 20.452222 |  |  |  | KP640378 |  |  |
| *Peripatopsis overbergiensis* |  | KLB9 | Marloth Reserve, WC | -33.995556 | 20.452222 |  |  |  | KP640382 |  |  |
| *Peripatopsis overbergiensis* |  | KLB10 | Marloth Reserve, WC | -33.995556 | 20.452222 |  |  |  | KP640373 |  |  |
| *Peripatopsis overbergiensis* |  | MLTHerm1 | Marloth Reserve, WC | -33.988056 | 20.421667 |  |  |  | KP640371 |  |  |
| *Peripatopsis overbergiensis* |  | MLTHerm2 | Marloth Reserve, WC | -33.988056 | 20.421667 |  |  |  | KP640371 |  |  |
| *Peripatopsis overbergiensis* |  | MLTHerm3 | Marloth Reserve, WC | -33.988056 | 20.421667 |  |  |  | KP640371 |  |  |
| *Peripatopsis overbergiensis* |  | MLTHerm4 | Marloth Reserve, WC | -33.988056 | 20.421667 |  |  |  | KP640372 |  |  |
| *Peripatopsis overbergiensis* |  | MLTHerm5 | Marloth Reserve, WC | -33.988056 | 20.421667 |  |  |  | KP640380 |  |  |
| *Peripatopsis overbergiensis* |  | MLTHerm6 | Marloth Reserve, WC | -33.988056 | 20.421667 |  |  |  | KP640380 |  |  |
| *Peripatopsis overbergiensis* |  | KA1 | De Hoop Reserve, WC | -34.379167 | 20.541389 |  |  |  | KP640385 |  |  |
| *Peripatopsis overbergiensis* |  | KA2 | De Hoop Reserve, WC | -34.379167 | 20.541389 |  |  |  | KP640385 |  |  |
| *Peripatopsis overbergiensis* |  | KA3 | De Hoop Reserve, WC | -34.379167 | 20.541389 |  |  |  | KP640385 |  |  |
| *Peripatopsis capensis* |  | MyburghDM1 | Myburgh Ravine, WC | -34.00535556 | 18.37832222 |  |  |  | JN798078 |  |  |
| *Peripatopsis capensis* |  | MyburghDM2 | Myburgh Ravine, WC | -34.00535556 | 18.37832222 |  |  |  | JN798077 |  |  |
| *Peripatopsis capensis* |  | MyburghDM3 | Myburgh Ravine, WC | -34.00535556 | 18.37832222 |  |  |  | JN798076 |  |  |
| *Peripatopsis capensis* |  | MyburghDM4 | Myburgh Ravine, WC | -34.00535556 | 18.37832222 |  |  |  | JN798075 |  |  |
| *Peripatopsis capensis* |  | ORANGEDM1 | Orangekloof, WC | -34.00435833 | 18.38295556 |  |  |  | JN798079 |  |  |
| *Peripatopsis capensis* |  | ORANGEDM2 | Orangekloof, WC | -34.00435833 | 18.38295556 |  |  |  | JN798080 |  |  |
| *Peripatopsis capensis* |  | ORANGEDM3 | Orangekloof, WC | -34.00435833 | 18.38295556 |  |  |  | JN798081 |  |  |
| *Peripatopsis capensis* |  | ORANGEDM5 | Orangekloof, WC | -34.00435833 | 18.38295556 |  |  |  | JN798082 |  |  |
| *Peripatopsis capensis* |  | RHODESDM4 | Rhodes Memorial, WC | -33.95270278 | 18.45129444 |  |  |  | JN798083 |  |  |
| *Peripatopsis capensis* |  | RHODESDM5 | Rhodes Memorial, WC | -33.95270278 | 18.45129444 |  |  |  | JN798084 |  |  |
| *Peripatopsis capensis* |  | RHODESDM6 | Rhodes Memorial, WC | -33.95270278 | 18.45129444 |  |  |  | JN798085 |  |  |
| *Peripatopsis capensis* |  | RHODESDM7 | Rhodes Memorial, WC | -33.95270278 | 18.45129444 |  |  |  | JN798086 |  |  |
| *Peripatopsis capensis* |  | RHODESDM8 | Rhodes Memorial, WC | -33.95270278 | 18.45129444 |  |  |  | JN798087 |  |  |
| *Peripatopsis capensis* |  | RHODESDM9 | Rhodes Memorial, WC | -33.95270278 | 18.45129444 |  |  |  | JN798088 |  |  |
| *Peripatopsis capensis* |  | SkeletonDM1 | Skeleton Gorge, WC | -33.98 | 18.42 |  |  |  | JN798089 |  |  |
| *Peripatopsis capensis* |  | SkeletonDM2 | Skeleton Gorge, WC | -33.98 | 18.42 |  |  |  | JN798090 |  |  |
| *Peripatopsis capensis* |  | SkeletonDM3 | Skeleton Gorge, WC | -33.98 | 18.42 |  |  |  | JN798091 |  |  |
| *Peripatopsis capensis* |  | SKELETONG1 | Skeleton Gorge, WC | -33.98 | 18.42 |  |  |  | EU855332 |  |  |
| *Peripatopsis capensis* |  | NEWLANDSF1 | Newlands Forest, WC | -33.95430556 | 18.44473056 |  |  |  | EU855321 |  |  |
| *Peripatopsis capensis* |  | NEWLANDSF2 | Newlands Forest, WC | -33.95430556 | 18.44473056 |  |  |  | EU855322 |  |  |
| *Peripatopsis capensis* |  | NEWLANDSF3 | Newlands Forest, WC | -33.95430556 | 18.44473056 |  |  |  | EU855323 |  |  |
| *Peripatopsis capensis* |  | CeceliaDM1 | Cecelia Forest, WC | -33.96 | 18.42 |  |  |  | JN798092 |  |  |
| *Peripatopsis capensis* |  | CeceliaDM2 | Cecelia Forest, WC | -33.96 | 18.42 |  |  |  | JN798093 |  |  |
| *Peripatopsis capensis* |  | CeceliaDM3 | Cecelia Forest, WC | -33.96 | 18.42 |  |  |  | JN798094 |  |  |
| *Peripatopsis capensis* |  | CeceliaDM4 | Cecelia Forest, WC | -33.96 | 18.42 |  |  |  | JN798095 |  |  |
| *Peripatopsis capensis* |  | CeceliaF2 | Cecelia Forest, WC | -33.96 | 18.42 |  |  |  | EU855329 |  |  |
| *Peripatopsis capensis* |  | CeceliaF4 | Cecelia Forest, WC | -33.96 | 18.42 |  |  |  | EU855330 |  |  |
| *Peripatopsis capensis* |  | CeceliaF5 | Cecelia Forest, WC | -33.96 | 18.42 |  |  |  | EU855331 |  |  |
| *Peripatopsis lawrencei* |  | DAPDM1 | Dappat se Gat, WC | -34.22384444 | 18.84004444 |  |  |  | JN798096 |  |  |
| *Peripatopsis lawrencei* |  | DAPDM2 | Dappat se Gat, WC | -34.22384444 | 18.84004444 |  |  |  | JN798097 |  |  |
| *Peripatopsis lawrencei* |  | DAPDM3 | Dappat se Gat, WC | -34.22384444 | 18.84004444 |  |  |  | JN798098 |  |  |
| *Peripatopsis lawrencei* |  | DAPDM5 | Dappat se Gat, WC | -34.22384444 | 18.84004444 |  |  |  | JN798099 |  |  |
| *Peripatopsis lawrencei* |  | DAPDM6 | Dappat se Gat, WC | -34.22384444 | 18.84004444 |  |  |  | JN798100 |  |  |
| *Peripatopsis lawrencei* |  | CALEDONDM1 | Caledon, WC | -34.21194444 | 19.50638889 |  |  |  | JN798101 |  |  |
| *Peripatopsis lawrencei* |  | Franschoek1 | Franschoek, WC | -33.89749061 | 19.15233736 |  |  |  |  |  |  |
| *Peripatopsis lawrencei* |  | Franschoek2 | Franschoek, WC | -33.89749061 | 19.15233736 |  |  |  |  |  |  |
| *Peripatopsis lawrencei* |  | Franschoek3 | Franschoek, WC | -33.89749061 | 19.15233736 |  |  |  |  |  |  |
| *Peripatopsis lawrencei* |  | Franschoek4 | Franschoek, WC | -33.89749061 | 19.15233736 |  |  |  |  |  |  |
| *Peripatopsis lawrencei* |  | JONKERDM1 | Jonkershoek, WC | -33.96826667 | 18.94018056 |  |  |  | JN798102 |  |  |
| *Peripatopsis lawrencei* |  | JONKERDM2 | Jonkershoek, WC | -33.96826667 | 18.94018056 |  |  |  | JN798103 |  |  |
| *Peripatopsis lawrencei* |  | JONKERDM3 | Jonkershoek, WC | -33.96826667 | 18.94018056 |  |  |  | JN798104 |  |  |
| *Peripatopsis lawrencei* |  | JONKERDM4 | Jonkershoek, WC | -33.96826667 | 18.94018056 |  |  |  | JN798105 |  |  |
| *Peripatopsis lawrencei* |  | KogelbDM3 | Kogelberg, WC | -34.33291944 | 18.95148333 |  |  |  | JN798106 |  |  |
| *Peripatopsis lawrencei* |  | KogelbDM4 | Kogelberg, WC | -34.33291944 | 18.95148333 |  |  |  | JN798107 |  |  |
| *Peripatopsis lawrencei* |  | RIVERSOND2 | Riviersonderend, WC | -34.01333333 | 19.83 |  |  |  | EU855356 |  |  |
| *Peripatopsis lawrencei* |  | OUBOSDM7 | Oubos, WC | -34.07620278 | 19.82882222 |  |  |  | JN798109 |  |  |
| *Peripatopsis lawrencei* |  | OUBOSDM8 | Oubos, WC | -34.07620278 | 19.82882222 |  |  |  | JN798108 |  |  |
| *Peripatopsis lawrencei* |  | OubosDM1 | Oubos, WC | -34.07620278 | 19.82882222 |  |  |  | JN798110 |  |  |
| *Peripatopsis lawrencei* |  | OubosDM2 | Oubos, WC | -34.07620278 | 19.82882222 |  |  |  | JN798111 |  |  |
| *Peripatopsis lawrencei* |  | Rondevlei | Rondevlei, WC | -34.06041667 | 18.49993333 |  |  |  | JN798112 |  |  |
| *Peripatopsis lawrencei* |  | NAPIER1 | Napier, WC | -34.48992222 | 19.72229167 |  |  |  | JN798118 |  |  |
| *Peripatopsis lawrencei* |  | NAPIER2 | Napier, WC | -34.48992222 | 19.72229167 |  |  |  | JN798117 |  |  |
| *Peripatopsis lawrencei* |  | NAPIER3 | Napier, WC | -34.48992222 | 19.72229167 |  |  |  | JN798115 |  |  |
| *Peripatopsis lawrencei* |  | NAPIER4 | Napier, WC | -34.48992222 | 19.72229167 |  |  |  | JN798114 |  |  |
| *Peripatopsis lawrencei* |  | NAPIER5 | Napier, WC | -34.48992222 | 19.72229167 |  |  |  | JN798113 |  |  |
| *Peripatopsis lawrencei* |  | NapierRed | Napier, WC | -34.48992222 | 19.72229167 |  |  |  | JN798116 |  |  |
| *Peripatopsis lawrencei* |  | HIGHNOON1 | High Noon, WC | -33.90811944 | 19.28223611 |  |  |  | EU855288 |  |  |
| *Peripatopsis lawrencei* |  | HIGHNOON2 | High Noon, WC | -33.90811944 | 19.28223611 |  |  |  | EU855289 |  |  |
| *Peripatopsis lawrencei* |  | HIGHNOON3 | High Noon, WC | -33.90811944 | 19.28223611 |  |  |  | EU855290 |  |  |
| *Peripatopsis lawrencei* |  | HIGHNOON4 | High Noon, WC | -33.90811944 | 19.28223611 |  |  |  | EU855291 |  |  |
| *Peripatopsis lawrencei* |  | GREYTON1 | Greyton, WC | -34.03375556 | 19.61592222 |  |  |  | EU855340 |  |  |
| *Peripatopsis lawrencei* |  | GREYTON2 | Greyton, WC | -34.03375556 | 19.61592222 |  |  |  | EU855341 |  |  |
| *Peripatopsis lawrencei* |  | GREYTON3 | Greyton, WC | -34.03375556 | 19.61592222 |  |  |  | EU855342 |  |  |
| *Peripatopsis lawrencei* |  | GrootbosDM1 | Grootbos, WC | -34.57111111 | 19.42277778 |  |  |  | JN798122 |  |  |
| *Peripatopsis lawrencei* |  | GrootbosDM3 | Grootbos, WC | -34.57111111 | 19.42277778 |  |  |  | JN798121 |  |  |
| *Peripatopsis lawrencei* |  | GrootbosDM4 | Grootbos, WC | -34.57111111 | 19.42277778 |  |  |  | JN798120 |  |  |
| *Peripatopsis lawrencei* |  | GrootbosDM5 | Grootbos, WC | -34.57111111 | 19.42277778 |  |  |  | JN798119 |  |  |
| *Peripatopsis lawrencei* |  | GROOTBOS1 | Grootbos, WC | -34.57111111 | 19.42277778 |  |  |  | EU855344 |  |  |
| *Peripatopsis lawrencei* |  | GROOTBOS2 | Grootbos, WC | -34.57111111 | 19.42277778 |  |  |  | EU855345 |  |  |
| *Peripatopsis lawrencei* |  | GROOTBOS3 | Grootbos, WC | -34.57111111 | 19.42277778 |  |  |  | EU855346 |  |  |
| *Peripatopsis lawrencei* |  | GROOTBOS4 | Grootbos, WC | -34.57111111 | 19.42277778 |  |  |  | EU855347 |  |  |
| *Peripatopsis lawrencei* |  | GROOTBOS5 | Grootbos, WC | -34.57111111 | 19.42277778 |  |  |  | EU855348 |  |  |
| *Peripatopsis lawrencei* |  | FernkDM1 | Fernkloof Reserve, WC | -34.39361111 | 19.27611111 |  |  |  | JN798123 |  |  |
| *Peripatopsis lawrencei* |  | FernkDM2 | Fernkloof Reserve, WC | -34.39361111 | 19.27611111 |  |  |  | JN798124 |  |  |
| *Peripatopsis lawrencei* |  | FernkDM3 | Fernkloof Reserve, WC | -34.39361111 | 19.27611111 |  |  |  | JN798125 |  |  |
| *Peripatopsis lawrencei* |  | FernkDM4 | Fernkloof Reserve, WC | -34.39361111 | 19.27611111 |  |  |  | JN798126 |  |  |
| *Peripatopsis lawrencei* |  | FERNKLOOF4 | Fernkloof Reserve, WC | -34.39361111 | 19.27611111 |  |  |  | EU855336 |  |  |
| *Peripatopsis overbergiensis* |  | GrootvdbDM1 | Grootvadersbosch Reserve, WC | -33.98416667 | 20.82416667 |  |  |  | JN798129 |  |  |
| *Peripatopsis overbergiensis* |  | GrootvdbDM4 | Grootvadersbosch Reserve, WC | -33.98416667 | 20.82416667 |  |  |  | JN798128 |  |  |
| *Peripatopsis overbergiensis* |  | GrootvdbDM5 | Grootvadersbosch Reserve, WC | -33.98416667 | 20.82416667 |  |  |  | JN798127 |  |  |
| *Peripatopsis overbergiensis* |  | GROOTVADER1 | Grootvadersbosch Reserve, WC | -33.98416667 | 20.82416667 |  |  |  | EU855311 |  |  |
| *Peripatopsis overbergiensis* |  | GROOTVADER2 | Grootvadersbosch Reserve, WC | -33.98416667 | 20.82416667 |  |  |  | EU855312 |  |  |
| *Peripatopsis overbergiensis* |  | GROOTVADER3 | Grootvadersbosch Reserve, WC | -33.98416667 | 20.82416667 |  |  |  | EU855313 |  |  |
| *Peripatopsis overbergiensis* |  | GROOTVADER4 | Grootvadersbosch Reserve, WC | -33.98416667 | 20.82416667 |  |  |  | EU855314 |  |  |
| *Peripatopsis overbergiensis* |  | GROOTVADER5 | Grootvadersbosch Reserve, WC | -33.98416667 | 20.82416667 |  |  |  | EU855315 |  |  |
| *Peripatopsis overbergiensis* |  | MARLOTHDM1 | Marloth Reserve, WC | -33.997733 | 20.457689 |  |  |  | JN798136 |  |  |
| *Peripatopsis overbergiensis* |  | MARLOTHDM2 | Marloth Reserve, WC | -33.997733 | 20.457689 |  |  |  | JN798135 |  |  |
| *Peripatopsis overbergiensis* |  | MARLOTHDM4 | Marloth Reserve, WC | -33.997733 | 20.457689 |  |  |  | JN798134 |  |  |
| *Peripatopsis overbergiensis* |  | MARLOTHDM5 | Marloth Reserve, WC | -33.997733 | 20.457689 |  |  |  | JN798133 |  |  |
| *Peripatopsis overbergiensis* |  | MARLOTHDM6 | Marloth Reserve, WC | -33.997733 | 20.457689 |  |  |  | JN798132 |  |  |
| *Peripatopsis overbergiensis* |  | MARLOTHDM7 | Marloth Reserve, WC | -33.997733 | 20.457689 |  |  |  | JN798131 |  |  |
| *Peripatopsis overbergiensis* |  | MARLOTHDM8 | Marloth Reserve, WC | -33.997733 | 20.457689 |  |  |  | JN798130 |  |  |
| *Peripatopsis overbergiensis* |  | MARLOTHNR1 | Marloth Reserve, WC | -33.997733 | 20.457689 |  |  |  | EU855284 |  |  |
| *Peripatopsis overbergiensis* |  | MARLOTHNR2 | Marloth Reserve, WC | -33.997733 | 20.457689 |  |  |  | EU855285 |  |  |
| *Peripatopsis overbergiensis* |  | MARLOTHNR4 | Marloth Reserve, WC | -33.997733 | 20.457689 |  |  |  | EU855286 |  |  |
| *Peripatopsis overbergiensis* |  | PotbergDM2 | De Hoop Reserve, WC | -34.29305556 | 20.48861111 |  |  |  | JN798141 |  |  |
| *Peripatopsis overbergiensis* |  | PotbergDM3 | De Hoop Reserve, WC | -34.29305556 | 20.48861111 |  |  |  | JN798140 |  |  |
| *Peripatopsis overbergiensis* |  | PotbergDM4 | De Hoop Reserve, WC | -34.29305556 | 20.48861111 |  |  |  | JN798139 |  |  |
| *Peripatopsis overbergiensis* |  | PotbergDM5 | De Hoop Reserve, WC | -34.29305556 | 20.48861111 |  |  |  | JN798138 |  |  |
| *Peripatopsis overbergiensis* |  | PotbergDM6 | De Hoop Reserve, WC | -34.29305556 | 20.48861111 |  |  |  | JN798137 |  |  |
| *Peripatopsis overbergiensis* |  | POTBERG1 | De Hoop Reserve, WC | -34.29305556 | 20.48861111 |  |  |  | EU855316 |  |  |
| *Peripatopsis overbergiensis* |  | POTBERG2 | De Hoop Reserve, WC | -34.29305556 | 20.48861111 |  |  |  | EU855317 |  |  |
| *Peripatopsis overbergiensis* |  | POTBERG3 | De Hoop Reserve, WC | -34.29305556 | 20.48861111 |  |  |  | EU855318 |  |  |
| *Peripatopsis overbergiensis* |  | POTBERG4 | De Hoop Reserve, WC | -34.29305556 | 20.48861111 |  |  |  | EU855319 |  |  |
| *Peripatopsis overbergiensis* |  | POTBERG5 | De Hoop Reserve, WC | -34.29305556 | 20.48861111 |  |  |  | EU855320 |  |  |
| *Peripatopsis birgeri** |  | KARKLOOF1 | Karkloof Reserve, KZN |  |  |  |  |  | EU855276 |  |  |
| *Peripatopsis moseleyi** |  | HOGSBACK1 | Hogsback, EC |  |  |  |  |  | EU855278 |  |  |
