# Supplementary material for: Between the Cape Fold Mountains and the deep blue sea: Comparative phylogeography of selected codistributed ectotherms reveals asynchronous cladogenesis
Source: Evol Appl. 2022 Oct 27;15(12):1967–87. doi: 10.1111/eva.13493 (PMC9753840; doi:10.1111/eva.13493)
Supplement: Supplementary file 10 — Appendix S3 [file EVA-15-1967-s001.docx]

**APPENDIX 3 |** **Supplementary tables**

**TABLE S3.1** Number of sequences used per locus for each taxon. The number of taxa from each phylogeographic region are indicated below in parenthesis (west/east)

|  | *H. areolatus* | *C. angulata* | *D. lutrix* | *A. meleagris* | *P. brincki*  + *P. parvicorpus*  *+ P. tuerkayi* | *P. perlatus*  + *P. barbarai*  *+ P. barnardi* | *Per. capensis*  *+ Per. lawrencei +*  *Per. overbergiensis* |
| --- | --- | --- | --- | --- | --- | --- | --- |
| ND4 | 68  (35/33) | 153  (115/38) | 73  (21/52) |  |  |  |  |
| cytb |  | 126  (85/41) | 71  (21/50) |  |  |  |  |
| 16S |  |  |  |  |  | 122  (31/91) |  |
| COI |  |  |  | 236  (46/188) | 76  (37/39) | 122  (31/91) | 161  (28/133) |
| PRLR | 14  (6/8) |  |  |  |  |  |  |
| EXPH5 |  |  |  | 52  (5/47) |  |  |  |
| SPTBN1 |  |  | 25  (7/18) |  |  |  |  |

**TABLE S3.2** Mutation rates used to date each phylogeny

|  | Mean | Lower Bound | Upper Bound |
| --- | --- | --- | --- |
| *Homopus areolatus* | | | |
| ND4 | 0.40 | 0.33 | 0.60 |
| *Chersina angulata* | | | |
| ND4 | 0.40 | 0.33 | 0.60 |
| cytb | 0.40 | 0.20 | 0.60 |
| *Duberria lutrix* | | | |
| ND4 | 1.34 |  |  |
| cytb | 1.34 |  |  |
| *Acontias meleagris* | | | |
| COI | 0.65 | 0.61 | 0.75 |
| *Potamonautes brincki + P. parvicorpus + P. tuerkayi* | | | |
| COI | 2.0 | 1.40 | 2.60 |
| *Potamonautes perlatus + P. barbarai + P. barnardi* | | | |
| COI | 2.0 | 1.40 | 2.60 |
| 16S | 1.02 | 0.64 | 1.42 |
| *Potamonautes capensis + P. lawrencei + P. overbergiensis* | | | |
| COI | 1.9 | 1.5 | 2.3 |

*Note:* All values are in % per million years

**TABLE S3.3** Best-fit substitution models determined using IQ-TREE under the Bayesian information criterion

|  | *H. areolatus* | *C. angulata* | *D. lutrix* | *A. meleagris* | *P. brincki*  + *P. parvicorpus*  *+ P. tuerkayi* | *P. perlatus*  + *P. barbarai*  *+ P. barnardi* | *Per. capensis*  *+ Per. lawrencei +*  *Per. overbergiensis* |
| --- | --- | --- | --- | --- | --- | --- | --- |
| ND4 | HKY+F+G4 | HKY+F+G4 | HKY+F+I |  |  |  |  |
| cytb |  | HKY+F+G4 | TPM2u+F+I |  |  |  |  |
| 16S |  |  |  |  |  | HKY+F+I |  |
| COI |  | TN+F+G4 |  | TPM3u+F+I+G4 | TIM2+F+G4 | HKY+F+G4 | TIM2+F+G4 |

**TABLE S3.4** Results of the hierarchical AMOVA for the mtDNA loci used in the study among the populations delineated by the phylogeographic regions, among the localities within each population, and within each locality

|  | Among populations | | | Among localities | | | Within localities | | |
| --- | --- | --- | --- | --- | --- | --- | --- | --- | --- |
|  | Va | % | *F*_CT_ | Vb | % | *F*_SC_ | Vc | % | *F*_ST_ |
| ***Homopus areolatus*** | | | | | | | | | |
| ND4 | 7.51 | **75.90** | 0.76 | 1.66 | **16.80** | 0.70 | 0.72 | **7.95** | 0.93 |
| ***Chersina angulata*** | | | | | | | | | |
| cytb | 1.86 | **57.33** | 0.57 | 0.86 | **26.37** | 0.62 | 0.53 | **16.29** | 0.84 |
| ND4 | 3.67 | **54.72** | 0.55 | 2.06 | **30.75** | 0.68 | 0.97 | **14.53** | 0.85 |
| ***Duberria lutrix*** | | | | | | | | | |
| cytb | 5.83 | **83.13** | 0.83 | 0.88 | **12.51** | 0.74 | 0.31 | **4.36** | 0.96 |
| ND4 | 11.60 | **86.22** | 0.86 | 1.38 | **10.24** | 0.74 | 0.48 | **3.53** | 0.96 |
| ***Acontias meleagris*** | | | | | | | | | |
| COI | 10.65 | **58.29** | 0.58 | 6.55 | **35.83** | 0.86 | 1.07 | **5.88** | 0.94 |
| ***Potamonautes brincki*** **+ *Potamoautes parvicorpus + Potamonautes tuerkayi*** | | | | | | | | | |
| COI | 20.29 | **76.18** | 0.76 | 5.76 | **21.61** | 0.91 | 0.59 | **2.21** | 0.98 |
| *Potamonautes brincki* | | | | | | | | | |
| COI |  |  |  | 1.67 | **81.35** | - | 0.38 | **18.65** | 0.81 |
| *Potamonautes parvicorpus* | | | | | | | | | |
| COI |  |  |  | 4.12 | **96.44** | - | 0.15 | **3.56** | 0.96 |
| *Potamonautes tuerkayi* | | | | | | | | | |
| COI |  |  |  | 2.15 | **58.29** | - | 1.54 | **41.71** | 0.58 |
| ***Potamonautes perlatus*** ***+ Potamonautes barnardi + Potamonautes barbarai*** | | | | | | | | | |
| 16S | 5.75 | **81.62** | 0.82 | 0.85 | **12.10** | 0.66 | 1.44 | **6.28** | 0.94 |
| COI | 10.07 | **48.69** | 0.49 | 8.79 | **42.52** | 0.83 | 1.82 | **8.79** | 0.91 |
| *Potamonautes perlatus* | | | | | | | | | |
| 16S |  |  |  | 0.70 | **71.60** | - | 0.28 | **28.40** | 0.72 |
| COI |  |  |  | 3.83 | **95.35** | - | 0.19 | **4.65** | 0.95 |
| *Potamonautes barnardi* | | | | | | | | | |
| 16S |  |  |  | 1.10 | **58.33** | - | 0.79 | **41.67** | 0.58 |
| COI |  |  |  | 19.70 | **60.99** | - | 12.60 | **39.01** | 0.61 |
| *Potamonautes barbarai* | | | | | | | | | |
| 16S |  |  |  | 0.44 | **50.50** | - | 0.43 | **49.50** | 0.50 |
| COI |  |  |  | 0.70 | **63.54** | - | 0.40 | **36.46** | 0.64 |
| ***Peripatopsis capensis + Peripatopsis lawrencei + Peripatopsis overbergiensis*** | | | | | | | | | |
| COI | 14.47 | **53.94** | 0.54 | 10.93 | **40.74** | 0.88 | 1.43 | **5.32** | 0.95 |
| *Peripatopsis capensis* | | | | | | | | | |
| COI |  |  |  | 2.27 | **63.3** | - | 1.31 | **36.7** | 0.63 |
| *Peripatopsis lawrencei* | | | | | | | | | |
| COI |  |  |  | 6.42 | **81.84** | - | 1.42 | **18.16** | 0.82 |
| *Peripatopsis overbergiensis* | | | | | | | | | |
| COI |  |  |  | 5.46 | **78.89** | - | 1.46 | **21.11** | 0.79 |

**TABLE S3.5** AMOVA showing the variation among and within the localities of the western and eastern phylogeographic regions

|  | West | | | | | East | | | | |
| --- | --- | --- | --- | --- | --- | --- | --- | --- | --- | --- |
|  | Among localities | | Within localities | |  | Among localities | | Within localities | |  |
|  | Va | % | Vb | % | Fst | Va | % | Vb | % | Fst |
| *Homopus areolatus* | | | | | | | | | | |
| ND4 | 0.46 | **48.14** | 0.50 | **51.86** | **0.48*** | 2.78 | **73.56** | 1.00 | **26.44** | 0.74 |
| *Chersina angulata* | | | | | | | | | | |
| *cytb* | 0.90 | **61.13** | 0.57 | **38.87** | 0.61 | 0.75 | **62.99** | 0.44 | **37.01** | 0.63 |
| ND4 | 2.05 | **66.56** | 1.03 | **33.44** | 0.67 | 2.12 | **72.30** | 0.81 | **27.70** | 0.72 |
| *Duberria lutrix* | | | | | | | | | | |
| cytb | 0.03 | **33.63** | 0.07 | **66.37** | **0.34*** | 1.16 | **73.65** | 0.42 | **26.35** | 0.74 |
| ND4 | 0.08 | **20.33** | 0.33 | **79.67** | **0.41*** | 1.81 | **76.99** | 0.54 | **23.01** | 0.77 |
| *Acontius meleagris* | | | | | | | | | | |
| COI | 2.17 | **78.46** | 0.60 | **21.54** | 0.78 | 7.22 | **85.67** | 1.21 | **14.33** | 0.86 |
| *Potamonautes parvicorpus* | | | | | | *P. brincki* + *P. tuerkayi* | | | | |
| COI | 4.12 | **96.44** | 0.15 | **3.56** | 0.96 | 7.32 | **88.02** | 1.00 | **11.98** | 0.88 |
| *Peripatopsis capensis* | | | | | | *Per. lawrencei ­+ Per. overbergiensis* | | | | |
| COI | 2.27 | **63.30** | 1.31 | **36.70** | 0.63 | 12.63 | **89.70** | 1.45 | **10.30** | 0.90 |
| *Potamonautes perlatus* | | | | | | *P. barbarai + P. barnardi* | | | | |
| COI | 3.83 | **95.35** | 0.19 | **4.65** | 0.95 | 10.47 | **81.61** | 2.36 | **18.39** | 0.82 |
| *16S* | 0.70 | **71.60** | 0.28 | **28.40** | 0.72 | 0.90 | **64.71** | 0.49 | **35.29** | 0.65 |

*Note:* All values are statistically significant (p <0.05) excluding those marked with an asterisk (*)

**TABLE S3.6** Standard diversity indices and neutrality tests for each clade of each gene per species

| Species/Population | Locus | N | S | H | h | π | Tajima's *D* | Fu's *F*s |
| --- | --- | --- | --- | --- | --- | --- | --- | --- |
| *H. areolatus* | ND4 | 68 | 63 | 29 | 0.884 | 0.018 | -0.482 | -2.505 |
| West - A | ND4 | 34 | 14 | 10 | 0.583 | 0.002 | **-2.265** | **-6.253** |
| West - B | ND4 | 1 | na | 1 | na | na | na | na |
| East - C | ND4 | 3 | 0 | 1 | na | na | na | na |
| East - D | ND4 | 26 | 17 | 15 | 0.926 | 0.005 | -0.922 | -7.074 |
| East - E | ND4 | 4 | 7 | 2 | 0.500 | 0.005 | -0.817 | 3.251 |
| *C. angulata* | cytb | 126 | 38 | 38 | 0.919 | 0.014 | -1.194 | **-20.781** |
|  | ND4 | 100 | 62 | 31 | 0.938 | 0.011 | -0.829 | -3.789 |
| West - A | ctyb | 50 | 23 | 20 | 0.911 | 0.009 | -1.488 | **-10.843** |
|  | ND4 | 40 | 19 | 13 | 0.879 | 0.004 | -0.750 | -2.385 |
| West - B | ctyb | 35 | 6 | 6 | 0.316 | 0.001 | **-1.906** | **-4.122** |
|  | ND4 | 31 | 19 | 6 | 0.686 | 0.003 | -1.522 | 1.372 |
| East - C | ctyb | 41 | 12 | 12 | 0.849 | 0.007 | -0.810 | -4.134 |
|  | ND4 | 29 | 23 | 12 | 0.833 | 0.006 | -0.502 | -1.048 |
| *D. lutrix* | ctyb | 71 | 21 | 16 | 0.850 | 0.011 | 0.375 | -0.692 |
|  | ND4 | 73 | 41 | 17 | 0.909 | 0.019 | 1.396 | 4.301 |
| West - A | ctyb | 21 | 2 | 3 | 0.186 | 0.000 | -1.514 | -1.920 |
|  | ND4 | 21 | 3 | 4 | 0.648 | 0.001 | -0.137 | -0.494 |
| East - B | ctyb | 26 | 10 | 9 | 0.760 | 0.003 | -1.201 | -3.317 |
|  | ND4 | 27 | 5 | 6 | 0.764 | 0.002 | -0.218 | -1.260 |
| East - C | ctyb | 18 | 3 | 4 | 0.712 | 0.002 | 0.593 | 0.006 |
|  | ND4 | 19 | 6 | 4 | 0.591 | 0.002 | -0.723 | 0.616 |
| East - D | ctyb | 6 | 6 | 4 | 0.800 | 0.005 | -0.786 | -0.272 |
|  | ND4 | 6 | 8 | 4 | 0.800 | 0.005 | -0.623 | 0.314 |
| *A. meleagris* | COI | 236 | 94 | 60 | 0.948 | 0.029 | -0.688 | -8.440 |
| West - A | COI | 48 | 31 | 12 | 0.694 | 0.007 | -1.666 | -0.920 |
| East - B | COI | 120 | 48 | 43 | 0.962 | 0.012 | -1.017 | -19.293 |
| East - C | COI | 29 | 15 | 10 | 0.727 | 0.004 | -1.419 | -2.783 |
| East - D | COI | 19 | 11 | 8 | 0.795 | 0.005 | -0.696 | -1.523 |
| East - E | COI | 14 | 33 | 7 | 0.758 | 0.022 | 0.442 | 3.414 |
| East - F | COI | 5 | 2 | 3 | 0.700 | 0.001 | -0.973 | -0.829 |
| East - G | COI | 1 | na | 1 | na | na | na | na |
| *P. brincki s.l.* | COI | 76 | 85 | 19 | 0.929 | 0.053 | **2.172** | **16.679** |
| *P. parvicorpus* | COI | 37 | 28 | 7 | 0.802 | 0.012 | 0.286 | 6.110 |
| *P. brincki s.s.* | COI | 20 | 11 | 5 | 0.800 | 0.006 | 0.379 | 2.396 |
| *P. tuerkayi* | COI | 19 | 15 | 7 | 0.807 | 0.009 | 0.918 | 1.725 |
| *Per. capensis s.l.* | COI | 161 | 132 | 59 | 0.929 | 0.050 | 0.609 | -0.539 |
| *Per. capensis s.s.* | COI | 28 | 19 | 9 | 0.862 | 0.010 | 1.150 | 2.015 |
| *Per. lawrencei* | COI | 49 | 78 | 34 | 0.983 | 0.023 | -0.763 | -8.977 |
| *Per. overbergiensis* | COI | 84 | 37 | 20 | 0.799 | 0.017 | 1.339 | 2.211 |
| *P. perlatus s.l.* | 16S | 122 | 33 | 24 | 0.897 | 0.012 | -1.002 | -5.529 |
|  | COI | 122 | 171 | 34 | 0.897 | 0.045 | -0.988 | 5.656 |
| *P. perlatus s.s.* | 16S | 31 | 6 | 5 | 0.695 | 0.004 | -0.131 | 0.374 |
|  | COI | 31 | 25 | 10 | 0.832 | 0.012 | 0.781 | 2.321 |
| *P. barnardi* | 16S | 17 | 16 | 11 | 0.926 | 0.009 | -1.251 | -4.487 |
|  | COI | 17 | 155 | 15 | 0.978 | 0.094 | 0.451 | 0.793 |
| *P. barbarai* | 16S | 74 | 15 | 11 | 0.814 | 0.005 | -1.307 | -2.800 |
|  | COI | 74 | 15 | 9 | 0.749 | 0.003 | -0.940 | -0.418 |

*Note:* Statistically significant values (p <0.05) are indicated in bold

**TABLE S3.7** Mean divergence time estimates in million years before present (with minimum and maximum 95% HPD values) calculated in *BEAST for indivudual mtDNA loci for each taxon, as well as for combined mtDNA and total evidence where available

|  | ND4 | 16S | COI | cytb | mtDNA | nuDNA + mtDNA |
| --- | --- | --- | --- | --- | --- | --- |
| *H. areolatus* | 3.71  (1.76 - 5.68) |  |  |  |  | 3.59  (1.64 - 5.67) |
| *C. angulata* | 3.11  (0.88 - 6.36) |  |  | 1.41  (0.47 - 2.49) | 2.72  (0.86 - 5.01) |  |
| *D. lutrix* | 1.52  (0.40 - 2.51) |  |  | 0.12  (0.03 - 0.23) | 1.05  (0.32 - 1.68) | 0.85  (0.75 - 4.48) |
| *A. meleagris* |  |  | 7.31  (4.71 - 8.72) |  |  | 6.5  (2.44 - 10.58) |
| *P. brincki*  + *P. parvicorpus*  *+ P. tuerkayi* |  |  | 3.15  (0.49 - 4.21) |  |  |  |
| *P. perlatus*  + *P. barbarai*  *+ P. barnardi* |  | 2.55  (0.39 - 3.93) | 0.93  (0.02 - 3.51) |  | 5.88  (0.85 - 11.85) |  |
| *Per. capensis*  *+ Per. lawrencei +*  *Per. overbergiensis* |  |  | 2.55  (0.75 - 3.54) |  |  |  |

| TABLE S3.8 Combinations of bioclimatic variables used to construct the species distribution models per taxon | | | | | | | | | | | | | | | | | | | |
| --- | --- | --- | --- | --- | --- | --- | --- | --- | --- | --- | --- | --- | --- | --- | --- | --- | --- | --- | --- |
| Taxon | Bio1 | Bio2 | Bio3 | Bio4 | Bio5 | Bio6 | Bio7 | Bio8 | Bio9 | Bio10 | Bio11 | Bio12 | Bio13 | Bio14 | Bio15 | Bio16 | Bio17 | Bio18 | Bio19 |
| *H. areolatus* | X |  | X |  | X | X |  | X | X |  |  | X |  | X |  |  |  |  |  |
| *C. angulata* | X |  | X |  |  | X |  | X | X |  |  |  |  | X | X |  |  |  | X |
| *D. lutrix* | X |  | X |  | X | X |  |  | X |  |  | X |  | X | X |  |  |  |  |
| *A. meleagris* | X |  | X |  | X | X |  | X |  |  |  | X |  | X |  |  |  |  |  |
| *P. brincki*  + *P. parvicorpus*  *+ P. tuerkayi* |  | X | X |  |  |  |  |  |  | X |  |  |  |  | X |  | X |  | X |
| *P. perlatus*  + *P. barbarai*  *+ P. barnardi* | X | X |  |  |  |  |  | X | X |  |  |  |  |  |  |  |  | X | X |
| *Per. capensis +*  *Per. lawrencei +*  *Per. overbergiensis* |  |  | X |  |  |  | X | X | X |  |  | X |  | X |  |  |  |  |  |

| TABLE S3.9 Model parameters and evalutaion metrics used in MaxEnt to construct species distribution models and to infer aeras of climatic stability for each study species | | | | | | |
| --- | --- | --- | --- | --- | --- | --- |
| Taxon | Sample size | Feature class | Regularisation multiplier | AUC_test_ | AUC_TRAIN_ | Partitioning method |
| *H. areolatus* | 201 | H | 1.5 | 0.976 | 0.978 | block |
| *C. angulata* | 105 | LQH | 2 | 0.944 | 0.951 | block |
| *D. lutrix* | 30 | LQH | 2 | 0.985 | 0.987 | block |
| *A. meleagris* | 170 | H | 2 | 0.961 | 0.964 | block |
| *P. brincki*  + *P. parvicorpus*  *+ P. tuerkayi* | 13 | L | 1 | 0.997 | 0.998 | jackknife |
| *P. perlatus*  + *P. barbarai*  *+ P. barnardi* | 76 | L | 4 | 0.964 | 0.971 | block |
| *Per. capensis*  *+ Per. lawrencei*  *+ Per. overbergiensis* | 27 | LQ | 0.5 | 0.995 | 0.996 | block |

**TABLES S3.10** Climate variable statistics for the *Homopus areolatus* sampling localities

| **TABLE S3.10a** Standardised climatic variable loadings at *H. areolatus* sampling localities for the first two principal components. Strongly-loading variables are indicated in bold | | |
| --- | --- | --- |
|  | PC 1 (40.44%) | PC2 (29.37%) |
| Bio1 | 0.10345 | -0.11052 |
| Bio2 | 0.29477 | -0.062871 |
| Bio3 | -0.060787 | **-0.32818** |
| Bio4 | **0.31681** | 0.099299 |
| Bio5 | **0.3559** | -0.01548 |
| Bio6 | -0.12239 | -0.042184 |
| Bio7 | **0.30523** | 0.018772 |
| Bio8 | -0.10174 | -0.21479 |
| Bio9 | 0.13676 | 0.07416 |
| Bio10 | 0.24885 | -0.060798 |
| Bio11 | -0.076403 | -0.14418 |
| Bio12 | -0.26571 | **0.31671** |
| Bio13 | -0.11694 | **0.43209** |
| Bio14 | **-0.36363** | -0.077882 |
| Bio15 | 0.20628 | **0.34076** |
| Bio16 | -0.11169 | **0.43511** |
| Bio17 | **-0.35953** | -0.078229 |
| Bio18 | -0.24578 | -0.13064 |

| **TABLE S3.10b** Spearman's correlation between the standardised bioclimatic variables at *H. areolatus* sampling localities and longitude, with significant Spearman's rank coefficients (rs) and corresponding p-values indicated in bold | | |
| --- | --- | --- |
|  | rs | p |
| Bio1 | -0.0978261 | 0.656 |
| Bio2 | -0.2371542 | 0.2746 |
| **Bio3** | **0.4160079** | **0.04948** |
| **Bio4** | **-0.4594862** | **0.02864** |
| **Bio5** | **-0.4456522** | **0.0343** |
| Bio6 | 0.2144269 | 0.3243 |
| Bio7 | -0.305336 | 0.1563 |
| Bio8 | 0.4021739 | 0.05817 |
| **Bio9** | **-0.5128458** | **0.0134** |
| Bio10 | -0.3695652 | 0.08339 |
| Bio11 | 0.3942688 | 0.06365 |
| Bio12 | -0.1186064 | 0.5899 |
| **Bio13** | **-0.4291719** | **0.041** |
| **Bio14** | **0.6681546** | **0.0004932** |
| **Bio15** | **-0.8754941** | **2.70E-06** |
| **Bio16** | **-0.4759081** | **0.02171** |
| **Bio17** | **0.6404553** | **0.0009946** |
| **Bio18** | **0.7478358** | **4.09E-05** |
| **Bio19** | **-0.5849802** | **0.003976** |

| **TABLE S3.10d** Pairwise PERMANOVA post-hoc test results between *H. areolatus* clades. F statistics are shown below the diagonal, with p-values above the diagonal | | | | | |
| --- | --- | --- | --- | --- | --- |
|  | A | B | C | D | E |
| A |  | 0.2221 | **0.0047** | **0.0023** | **0.0222** |
| B | 3.164 |  | 0.2529 | 0.2019 | 0.6663 |
| C | 9.488 | 2.974 |  | 0.0541 | 0.1937 |
| D | 5.036 | 2.565 | 2.413 |  | 0.0883 |
| E | 5.617 | 0.9824 | 3.665 | 2.822 |  |

| **TABLE S3.10c** Standardized climatic variable PERMANOVA results at *H. areolatus* sampling localities | | |
| --- | --- | --- |
|  | populations | regions |
| Permutation N: | 9999 | 9999 |
| Total sum of squares: | 346.3 | 346.3 |
| Within-group sum of squares: | 181 | 272 |
| F: | 4.111 | 5.738 |
| p (same): | **0.0001** | **0.0003** |

| **TABLE S3.10e** Kruskal-Wallis test on the bioclimatic variables at *H. areolatus* sampling localities, with significant values indicated in bold | | | | | | | | |
| --- | --- | --- | --- | --- | --- | --- | --- | --- |
|  |  | H (chi2) | p (same) | A (N = 8) | B (N = 1) | C (N = 3) | D (N = 9) | E (N = 2) |
| Bio1 | Annual Mean Temperature | 3.3478 | 0.5014 | 17.5 | 17.74 | 17.42 | 17.61 | 15.35 |
| Bio2 | Mean Diurnal Range (Mean of monthly (max temp - min temp)) | 8.7624 | 0.06732 | 12.11 | 15.37 | 9.14 | 11.6 | 13.85 |
| **Bio3** | **Isothermality (BIO2/BIO7) (×100)** | **10.389** | **0.03436** | **51.74** | **57.11** | **55.08** | **53.07** | **53.1** |
| **Bio4** | **Temperature Seasonality (standard deviation ×100)** | **13.444** | **0.009296** | **417.09** | **433.69** | **258.44** | **360.22** | **455.21** |
| Bio5 | Max Temperature of Warmest Month | 8.5704 | 0.07278 | 29.97 | 32.1 | 25.6 | 28.65 | 28.89 |
| Bio6 | Min Temperature of Coldest Month | 6.6329 | 0.1566 | 6.34 | 5.19 | 8.48 | 7.27 | 2.86 |
| **Bio7** | **Temperature Annual Range (BIO5-BIO6)** | **10.533** | **0.03235** | **23.47** | **26.91** | **16.6** | **21.37** | **26.04** |
| **Bio8** | **Mean Temperature of Wettest Quarter** | **10.29** | **0.03582** | **12.67** | **12.36** | **18.08** | **13.55** | **10.13** |
| **Bio9** | **Mean Temperature of Driest Quarter** | **10.29** | **0.03582** | **22.24** | **22.76** | **14.96** | **21.49** | **18.31** |
| Bio10 | Mean Temperature of Warmest Quarter | 6.1078 | 0.1912 | 22.25 | 22.86 | 20.79 | 21.89 | 20.76 |
| Bio11 | Mean Temperature of Coldest Quarter | 9.1836 | 0.05667 | 12.44 | 12.36 | 14.32 | 13.15 | 9.74 |
| Bio12 | Annual Precipitation | 9.1063 | 0.0585 | 535.5 | 245 | 586 | 450 | 306 |
| **Bio13** | **Precipitation of Wettest Month** | **10.823** | **0.02862** | **91** | **39** | **67** | **61** | **36.5** |
| **Bio14** | **Precipitation of Driest Month** | **13.679** | **0.008392** | **14** | **5** | **38** | **22** | **16.5** |
| **Bio15** | **Precipitation Seasonality (Coefficient of Variation)** | **15.315** | **0.00409** | **63.96** | **57.1** | **18.48** | **37.71** | **25.69** |
| **Bio16** | **Precipitation of Wettest Quarter** | **10.115** | **0.03853** | **254** | **110** | **185** | **175** | **100** |
| **Bio17** | **Precipitation of Driest Quarter** | **12.914** | **0.01171** | **49.5** | **20** | **128** | **73** | **55** |
| **Bio18** | **Precipitation of Warmest Quarter** | **15.757** | **0.003363** | **49.5** | **25** | **133** | **75** | **58.5** |
| **Bio19** | **Precipitation of Coldest Quarter** | **9.6039** | **0.04766** | **253** | **110** | **145** | **175** | **100** |

**TABLES S3.11** Climate variable statistics for the *Chersina angulata* sampling localities

| **TABLE S3.11a** Standardised climatic variable loadings at *C. angulata* sampling localities for the first two principal components. Strongly-loading variables are indicated in bold | | |
| --- | --- | --- |
|  | PC 1 (42.74%) | PC2 (21.85%) |
| Bio1 | -0.1696 | **0.32821** |
| Bio2 | **-0.31982** | -0.095113 |
| Bio3 | -0.17388 | 0.17064 |
| Bio4 | -0.1657 | -0.2054 |
| Bio5 | -0.28493 | 0.044999 |
| Bio6 | 0.099142 | **0.43099** |
| Bio7 | -0.26161 | -0.18303 |
| Bio8 | -0.028963 | -0.040741 |
| Bio9 | -0.11037 | **0.37235** |
| Bio10 | -0.21976 | 0.14278 |
| Bio11 | -0.02931 | **0.4091** |
| Bio12 | **0.31671** | 0.086598 |
| Bio13 | 0.25483 | 0.17945 |
| Bio14 | **0.31126** | -0.088986 |
| Bio15 | -0.14483 | **0.32824** |
| Bio16 | 0.25978 | 0.18055 |
| Bio17 | **0.30852** | -0.079926 |
| Bio18 | 0.28963 | -0.15586 |

| **TABLE S3.11b** Spearman's correlation between the standardised bioclimatic variables at *C. angulata* sampling localities and longitude, with significant Spearman's rank coefficients (rs) and corresponding p-values indicated in bold | | |
| --- | --- | --- |
|  | rs | p |
| Bio1 | -0.4246753 | 0.05628 |
| Bio2 | -0.3896104 | 0.0818 |
| **Bio3** | **-0.6090909** | **0.004072** |
| Bio4 | 0.1987013 | 0.3862 |
| Bio5 | -0.3077922 | 0.1744 |
| Bio6 | -0.1818182 | 0.4285 |
| Bio7 | -0.0038961 | 0.9887 |
| Bio8 | -0.1415584 | 0.539 |
| Bio9 | -0.3597403 | 0.1098 |
| Bio10 | -0.2571429 | 0.2593 |
| Bio11 | -0.4025974 | 0.07149 |
| **Bio12** | **0.6493506** | **0.001865** |
| Bio13 | 0.4262509 | 0.05401 |
| **Bio14** | **0.8433037** | **1.58E-06** |
| Bio15 | -0.6766234 | 0.001029 |
| Bio16 | 0.4111725 | 0.06406 |
| **Bio17** | **0.8606691** | **5.56E-07** |
| **Bio18** | **0.8470283** | **1.28E-06** |
| Bio19 | 0.3072426 | 0.1755 |

| **TABLE S3.11c** Standardized climatic variable PERMANOVA results at *C. angulata* sampling localities | | |
| --- | --- | --- |
|  | populations | regions |
| Permutation N: | 9999 | 9999 |
| Total sum of squares: | 380 | 380 |
| Within-group sum of squares: | 256.4 | 327.7 |
| F: | 4.341 | 3.032 |
| p (same): | **0.0003** | **0.0219** |

| **TABLE S3.11d** Pairwise PERMANOVA post-hoc test results between *C. angulata* clades. F statistics are shown below the diagonal, with p-values above the diagonal | | | |
| --- | --- | --- | --- |
|  | A | B | C |
| A |  | **0.0014** | 0.0504 |
| B | 5.35 |  | **0.005** |
| C | 2.381 | 7.807 |  |

| **TABLE S3.11e** Kruskal-Wallis test on the bioclimatic variables at *C. angulata* sampling localities, with significant values indicated in bold | | | | | | |
| --- | --- | --- | --- | --- | --- | --- |
|  |  | H (chi2) | p (same) | A (N = 11) | B (N = 6) | C (N = 4) |
| Bio1 | Annual Mean Temperature | 2.7086 | 0.2581 | 16.84 | 19.92 | 17.16 |
| Bio2 | Mean Diurnal Range (Mean of monthly (max temp - min temp)) | 4.8083 | 0.09034 | 12.61 | 13.78 | 10.76 |
| **Bio3** | **Isothermality (BIO2/BIO7) (×100)** | **11.062** | **0.003963** | **53.24** | **62.08** | **54.67** |
| Bio4 | Temperature Seasonality (standard deviation ×100) | 2.6236 | 0.2693 | 421.68 | 333.43 | 314.92 |
| Bio5 | Max Temperature of Warmest Month | 1.7231 | 0.4225 | 29.63 | 30.19 | 27.24 |
| Bio6 | Min Temperature of Coldest Month | 0.99311 | 0.6086 | 6.48 | 7.09 | 7.48 |
| Bio7 | Temperature Annual Range (BIO5-BIO6) | 0.829 | 0.6607 | 24.32 | 23.05 | 19.55 |
| **Bio8** | **Mean Temperature of Wettest Quarter** | **12.667** | **0.001776** | **12.79** | **14.38** | **16.46** |
| Bio9 | Mean Temperature of Driest Quarter | 3.8863 | 0.1433 | 21.91 | 21.52 | 17.32 |
| Bio10 | Mean Temperature of Warmest Quarter | 0.96124 | 0.6184 | 21.95 | 22.05 | 21.03 |
| **Bio11** | **Mean Temperature of Coldest Quarter** | **6.767** | **0.03393** | **12.79** | **13.88** | **13.34** |
| **Bio12** | **Annual Precipitation** | **12.308** | **0.002125** | **338** | **174.5** | **469.5** |
| **Bio13** | **Precipitation of Wettest Month** | **9.5933** | **0.008257** | **59** | **27.5** | **55** |
| **Bio14** | **Precipitation of Driest Month** | **13.11** | **0.001423** | **11** | **4** | **24.5** |
| **Bio15** | **Precipitation Seasonality (Coefficient of Variation)** | **8.0419** | **0.01794** | **59.45** | **61.03** | **23.06** |
| **Bio16** | **Precipitation of Wettest Quarter** | **9.7772** | **0.007532** | **167** | **77** | **145** |
| **Bio17** | **Precipitation of Driest Quarter** | **13.806** | **0.001005** | **40** | **15.5** | **83.5** |
| **Bio18** | **Precipitation of Warmest Quarter** | **12.457** | **0.001973** | **42** | **16** | **84** |
| **Bio19** | **Precipitation of Coldest Quarter** | **9.6312** | **0.008103** | **162** | **76.5** | **128.5** |

**TABLES S3.12** Climate variable statistics for the *Duberria lutrix* sampling localities

| **TABLE** **S3.12a** Standardised climatic variable loadings at *D. lutrix* sampling localities for the first two principal components. Strongly-loading variables are indicated in bold | | |
| --- | --- | --- |
|  | PC 1 (38.32%) | PC2 (33.19%) |
| Bio1 | 0.26466 | 0.11839 |
| Bio2 | 0.11694 | **0.32485** |
| Bio3 | 0.28902 | -0.11474 |
| Bio4 | -0.075641 | **0.36786** |
| Bio5 | 0.092429 | **0.36107** |
| Bio6 | 0.056182 | -0.26919 |
| Bio7 | 0.03493 | **0.36218** |
| Bio8 | **0.31936** | -0.055055 |
| Bio9 | -0.20461 | 0.16954 |
| Bio10 | 0.17599 | 0.29053 |
| Bio11 | 0.26104 | -0.15402 |
| Bio12 | -0.14706 | -0.25809 |
| Bio13 | -0.2726 | -0.16925 |
| Bio14 | 0.21372 | -0.21791 |
| Bio15 | **-0.34055** | -0.00050715 |
| Bio16 | -0.29458 | -0.14348 |
| Bio17 | 0.2135 | -0.21603 |
| Bio18 | 0.24363 | -0.21173 |

| **TABLE** **S3.12b** Spearman's correlation between the standardised bioclimatic variables at *D. lutrix* sampling localities and longitude, with significant Spearman's rank coefficients (rs) and corresponding p-values indicated in bold | | |
| --- | --- | --- |
|  | rs | p |
| **Bio1** | **0.5707692** | **0.003388** |
| Bio2 | 0.2969231 | 0.1493 |
| **Bio3** | **0.7607692** | **1.69E-05** |
| Bio4 | -0.2584615 | 0.2114 |
| Bio5 | 0.1269231 | 0.5439 |
| Bio6 | 0.06 | 0.7754 |
| Bio7 | -0.0207692 | 0.9223 |
| **Bio8** | **0.6992308** | **0.0001506** |
| **Bio9** | **-0.4330769** | **0.03167** |
| Bio10 | 0.2915385 | 0.1571 |
| **Bio11** | **0.6061538** | **0.001637** |
| Bio12 | -0.0857857 | 0.6835 |
| **Bio13** | **-0.6066551** | **0.001304** |
| **Bio14** | **0.7645162** | **8.61E-06** |
| **Bio15** | **-0.9053846** | **2.01E-06** |
| **Bio16** | **-0.6670514** | **0.0002706** |
| **Bio17** | **0.8086992** | **9.98E-07** |
| **Bio18** | **0.8303194** | **2.82E-07** |
| **Bio19** | **-0.8137746** | **7.53E-07** |

| **TABLE S3.12c** Standardized climatic variable PERMANOVA results at *D. lutrix* sampling localities | | |
| --- | --- | --- |
|  | populations | regions |
| Permutation N: | 9999 | 9999 |
| Total sum of squares: | 456 | 456 |
| Within-group sum of squares: | 212.5 | 387.8 |
| F: | 8.019 | 4.043 |
| p (same): | **0.0001** | **0.0034** |

| **TABLE** **S3.12d** Pairwise PERMANOVA post-hoc test results between *D. lutrix* clades. F statistics are shown below the diagonal, with p-values above the diagonal | | | | |
| --- | --- | --- | --- | --- |
|  | A | B | C | D |
| A |  | 0.1398 | **0.0017** | **0.0027** |
| B | 1.827 |  | **0.0026** | **0.0004** |
| C | 8.67 | 6.049 |  | **0.0023** |
| D | 15.76 | 9.314 | 8.335 |  |

| **TABLE** **S3.12e** Kruskal-Wallis test on the bioclimatic variables at *D. lutrix* sampling localities, with significant values indicated in bold | | | | | | | |
| --- | --- | --- | --- | --- | --- | --- | --- |
|  |  | H (chi2) | p (same) | A (N = 6) | B (N = 7) | C (N = 6) | D (N = 6) |
| **Bio1** | **Annual Mean Temperature** | **11.94** | **0.007592** | **16.98** | **16.52** | **17.67** | **17.16** |
| Bio2 | Mean Diurnal Range (Mean of monthly (max temp - min temp)) | 4.2413 | 0.2366 | 10.73 | 10.08 | 11.69 | 10.18 |
| **Bio3** | **Isothermality (BIO2/BIO7) (×100)** | **14.227** | **0.002612** | **52.04** | **52.63** | **53.3** | **57.08** |
| **Bio4** | **Temperature Seasonality (standard deviation ×100)** | **8.2611** | **0.04091** | **361.55** | **322.74** | **370.46** | **268.79** |
| Bio5 | Max Temperature of Warmest Month | 7.3288 | 0.06212 | 27.98 | 25.64 | 28.82 | 26.04 |
| Bio6 | Min Temperature of Coldest Month | 2.1705 | 0.5378 | 7.41 | 7.73 | 7.2 | 8.25 |
| Bio7 | Temperature Annual Range (BIO5-BIO6) | 5.3719 | 0.1465 | 20.6 | 18.99 | 21.42 | 17.75 |
| **Bio8** | **Mean Temperature of Wettest Quarter** | **14.083** | **0.002795** | **12.64** | **12.76** | **13.65** | **17.64** |
| **Bio9** | **Mean Temperature of Driest Quarter** | **9.6026** | **0.02226** | **21.35** | **19.62** | **21.05** | **15.27** |
| **Bio10** | **Mean Temperature of Warmest Quarter** | **8.4945** | **0.03682** | **21.35** | **20.24** | **22.16** | **20.9** |
| **Bio11** | **Mean Temperature of Coldest Quarter** | **12.03** | **0.007281** | **12.64** | **12.76** | **13.27** | **14.3** |
| **Bio12** | **Annual Precipitation** | **14.132** | **0.002731** | **628.5** | **579** | **395** | **663.5** |
| **Bio13** | **Precipitation of Wettest Month** | **16.795** | **0.0007787** | **104.5** | **89** | **47.5** | **77** |
| **Bio14** | **Precipitation of Driest Month** | **15.489** | **0.001443** | **18** | **24** | **20.5** | **38** |
| **Bio15** | **Precipitation Seasonality (Coefficient of Variation)** | **18.983** | **0.0002756** | **63.48** | **47.04** | **30.25** | **20.36** |
| **Bio16** | **Precipitation of Wettest Quarter** | **17.679** | **0.0005121** | **293.5** | **262** | **133.5** | **211.5** |
| **Bio17** | **Precipitation of Driest Quarter** | **16.282** | **0.0009924** | **59.5** | **74** | **68.5** | **116.5** |
| **Bio18** | **Precipitation of Warmest Quarter** | **16.568** | **0.0008671** | **59.5** | **74** | **71** | **172.5** |
| **Bio19** | **Precipitation of Coldest Quarter** | **17.66** | **0.0005168** | **293.5** | **262** | **133.5** | **128** |

**TABLES S3.13** Climate variable statistics for the *Acontias meleagris* sampling localities

| **TABLE** **S3.13a** Standardised climatic variable loadings at *A. meleagris* sampling localities for the first two principal components. Strongly-loading variables are indicated in bold | | |
| --- | --- | --- |
|  | PC 1 (37.63%) | PC2 (23.36%) |
| Bio1 | -0.0066982 | 0.091452 |
| Bio2 | **-0.3512** | 0.053009 |
| Bio3 | -0.055411 | 0.28639 |
| Bio4 | **-0.32006** | -0.1277 |
| Bio5 | **-0.31058** | -0.050692 |
| Bio6 | 0.2985 | -0.03097 |
| Bio7 | **-0.34812** | -0.011874 |
| Bio8 | -0.18467 | **0.3524** |
| Bio9 | 0.15218 | **-0.315** |
| Bio10 | -0.20216 | 0.0088126 |
| Bio11 | 0.21115 | 0.1448 |
| Bio12 | 0.26076 | 0.14487 |
| Bio13 | 0.23866 | -0.064739 |
| Bio14 | 0.1987 | 0.3431 |
| Bio15 | 0.0072841 | **-0.38287** |
| Bio16 | 0.23996 | -0.072914 |
| Bio17 | 0.18921 | **0.34929** |
| Bio18 | 0.01099 | **0.37772** |

| **TABLE** **S3.13b** Spearman's correlation between the standardised bioclimatic variables at *A. meleagris* sampling localities and longitude, with significant Spearman's rank coefficients (rs) and corresponding p-values indicated in bold | | |
| --- | --- | --- |
|  | rs | p |
| Bio1 | 0.1704082 | 0.2409 |
| **Bio2** | **0.2831633** | **0.04901** |
| **Bio3** | **0.4463265** | **0.001452** |
| Bio4 | -0.0360204 | 0.8055 |
| Bio5 | -0.0718367 | 0.6228 |
| **Bio6** | **-0.3079592** | **0.03177** |
| Bio7 | 0.1796939 | 0.216 |
| **Bio8** | **0.6506122** | **8.90E-07** |
| **Bio9** | **-0.6759184** | **2.81E-07** |
| Bio10 | 0.03071429 | 0.8337 |
| Bio11 | 0.1396939 | 0.3373 |
| **Bio12** | **0.4307473** | **0.002004** |
| Bio13 | 0.1087995 | 0.4568 |
| **Bio14** | **0.5914521** | **7.64E-06** |
| **Bio15** | **-0.6654082** | **4.54E-07** |
| Bio16 | 0.08470036 | 0.5628 |
| **Bio17** | **0.6161189** | **2.44E-06** |
| **Bio18** | **0.9657847** | **2.20E-16** |
| **Bio19** | **-0.6103174** | **3.22E-06** |

| **TABLE** **S3.13c** Standardized climatic variable PERMANOVA results at *A. meleagris* sampling localities | | |
| --- | --- | --- |
|  | populations | regions |
| Permutation N: | 9999 | 9999 |
| Total sum of squares: | 912 | 912 |
| Within-group sum of squares: | 491.3 | 740.9 |
| F: | 5.993 | 10.86 |
| p (same): | **0.0001** | **0.0001** |

| **TABLE** **S3.13d** Pairwise PERMANOVA post-hoc test results between *A. meleagris* clades. F statistics are shown below the diagonal, with p-values above the diagonal | | | | | | | |
| --- | --- | --- | --- | --- | --- | --- | --- |
|  | A | B | C | D | E | F | G |
| A |  | **0.0001** | **0.0037** | 0.115 | **0.0017** | 0.0948 | 0.095 |
| B | 8.14 |  | **0.0001** | **0.004** | **0.0002** | 0.3853 | **0.0401** |
| C | 5.947 | 14.18 |  | **0.0016** | **0.0036** | 0.1271 | 0.1266 |
| D | 1.955 | 4.046 | 6.673 |  | **0.0077** | 0.3316 | 0.3339 |
| E | 12.25 | 7.727 | 12.22 | 10.96 |  | 0.595 | 0.6059 |
| F | 2.404 | 1.123 | 3.971 | 1.845 | 1.26 |  | 1 |
| G | 3.384 | 2.495 | 3.452 | 2.937 | 1.039 | 1.039 |  |

| **TABLE** **S3.13e** Kruskal-Wallis test on the bioclimatic variables at *A. meleagris* sampling localities, with significant values indicated in bold | | | | | | | | | | |
| --- | --- | --- | --- | --- | --- | --- | --- | --- | --- | --- |
|  |  | H (chi2) | p (same) | A (N=9) | B (N = 22) | C (N = 7) | D (N = 5) | E (N = 4) | F (N = 1) | G (N = 1) |
| Bio1 | Annual Mean Temperature | 8.2562 | 0.2199 | 17.57 | 17.56 | 16.7 | 17.42 | 18.29 | 18.15 | 17.71 |
| **Bio2** | **Mean Diurnal Range** | **27.503** | **0.0001166** | **12.07** | **13.72** | **9.24** | **9.97** | **9.8** | **10.7** | **9.25** |
| **Bio3** | **Isothermality (BIO2/BIO7) (×100)** | **16.018** | **0.01366** | **56.57** | **56.84** | **52.95** | **53.34** | **58.63** | **57.05** | **55.39** |
| **Bio4** | **Temperature Seasonality (standard deviation ×100)** | **17.057** | **0.009077** | **398.75** | **378.53** | **301.56** | **317.21** | **244.32** | **285.57** | **255.03** |
| **Bio5** | **Max Temperature of Warmest Month** | **17.571** | **0.0074** | **29.71** | **29.34** | **26.33** | **27.21** | **26.08** | **27.02** | **25.66** |
| **Bio6** | **Min Temperature of Coldest Month** | **27.28** | **0.0001283** | **7.23** | **4.81** | **8.98** | **8.28** | **9.65** | **8.25** | **8.95** |
| **Bio7** | **Temperature Annual Range** | **23.631** | **0.0006105** | **22.51** | **23.94** | **17.35** | **18.72** | **16.7** | **18.76** | **16.71** |
| **Bio8** | **Mean Temperature of Wettest Quarter** | **27.798** | **0.0001026** | **13.66** | **20.59** | **13.26** | **13.22** | **18.18** | **19.16** | **15.77** |
| **Bio9** | **Mean Temperature of Driest Quarter** | **29.184** | **5.61E-05** | **21.92** | **12.4** | **20.85** | **20.14** | **16.03** | **15.2** | **20.89** |
| Bio10 | Mean Temperature of Warmest Quarter | 7.8916 | 0.2462 | 21.93 | 21.82 | 20.85 | 21.11 | 21.33 | 21.62 | 20.89 |
| **Bio11** | **Mean Temperature of Coldest Quarter** | **14.433** | **0.02515** | **13.28** | **12.51** | **13.26** | **13.22** | **15.4** | **14.61** | **14.67** |
| **Bio12** | **Annual Precipitation** | **16.438** | **0.01159** | **304** | **449** | **621** | **450** | **665** | **503** | **665** |
| **Bio13** | **Precipitation of Wettest Month** | **14.397** | **0.02551** | **54** | **54.5** | **103** | **55** | **76** | **53** | **73** |
| **Bio14** | **Precipitation of Driest Month** | **25.04** | **0.0003357** | **6** | **17.5** | **16** | **20** | **36** | **33** | **40** |
| **Bio15** | **Precipitation Seasonality (Coefficient of Variation)** | **34.174** | **6.23E-06** | **65.93** | **41.06** | **65.99** | **35.74** | **27.86** | **16.22** | **17.64** |
| **Bio16** | **Precipitation of Wettest Quarter** | **13.932** | **0.03041** | **152** | **151.5** | **249** | **160** | **216** | **149** | **199** |
| **Bio17** | **Precipitation of Driest Quarter** | **25.242** | **0.000308** | **23** | **60.5** | **54** | **69** | **114** | **101** | **134** |
| **Bio18** | **Precipitation of Warmest Quarter** | **36.488** | **2.22E-06** | **23** | **124.5** | **54** | **72** | **190.5** | **128** | **139** |
| **Bio19** | **Precipitation of Coldest Quarter** | **33.003** | **1.05E-05** | **147** | **64.5** | **294** | **160** | **130.5** | **110** | **185** |

**TABLES S3.14** Climate variable statistics for the *Potamonautes brincki* *+ P. parvicorpus* *+ P. tuerkayi* sampling localities

| **TABLE** **S3.14b** Spearman's correlation between the standardised bioclimatic variables at *P. brincki* *+ P. parvicorpus + P. tuerkayi* sampling localities and longitude, with significant Spearman's rank coefficients (rs) and corresponding p-values indicated in bold | | |
| --- | --- | --- |
|  | rs | p |
| Bio1 | -0.2237762 | 0.4849 |
| Bio2 | 0.4335664 | 0.1614 |
| **Bio3** | **0.6363636** | **0.03011** |
| Bio4 | 0.2237762 | 0.4849 |
| Bio5 | 0.2307692 | 0.4709 |
| Bio6 | -0.5594406 | 0.06275 |
| Bio7 | 0.3566434 | 0.256 |
| Bio8 | -0.4755245 | 0.1213 |
| Bio9 | -0.0699301 | 0.8344 |
| Bio10 | -0.1118881 | 0.7328 |
| Bio11 | -0.4755245 | 0.1213 |
| Bio12 | 0.3356643 | 0.2867 |
| Bio13 | 0.05594406 | 0.869 |
| **Bio14** | **0.6930163** | **0.01247** |
| **Bio15** | **-0.8951049** | **5.94E-06** |
| Bio16 | 0.05253949 | 0.8712 |
| **Bio17** | **0.7205724** | **0.008201** |
| **Bio18** | **0.7062937** | **0.01329** |
| Bio19 | 0.05253949 | 0.8712 |

| **TABLE S3.14a** Standardised climatic variable loadings at *P. brincki + P. parvicorpus + P. tuerkayi* sampling localities for the first two principal components. Strongly-loading variables are indicated in bold | | |
| --- | --- | --- |
|  | PC 1 (46.38%) | PC2 (29.09%) |
| Bio1 | **0.30979** | 0.14425 |
| Bio2 | **0.033843** | **0.41863** |
| Bio3 | -0.04123 | 0.1413 |
| Bio4 | 0.073241 | **0.39272** |
| Bio5 | 0.17294 | **0.36266** |
| Bio6 | 0.2248 | **-0.30696** |
| Bio7 | 0.042618 | **0.4183** |
| Bio8 | **0.31651** | -0.095306 |
| Bio9 | 0.24526 | 0.22951 |
| Bio10 | 0.26075 | 0.25402 |
| Bio11 | **0.31651** | -0.095306 |
| Bio12 | -0.29892 | 0.079331 |
| Bio13 | -0.23275 | 0.0025025 |
| Bio14 | -0.2746 | 0.11816 |
| Bio15 | 0.037291 | -0.15877 |
| Bio16 | -0.25028 | 0.023689 |
| Bio17 | -0.26302 | 0.15445 |
| Bio18 | -0.2651 | 0.15235 |

| **TABLE** **S3.14d** Pairwise PERMANOVA post-hoc test results between *P. brincki + P. parvicorpus + P. tuerkayi* clades. F statistics are shown below the diagonal, with p-values above the diagonal | | | |
| --- | --- | --- | --- |
|  | A | B | C |
| A |  | 0.3294 | 0.1814 |
| B | 1.169 |  | 0.0676 |
| C | 1.531 | 3.437 |  |

| **TABLE** **S3.14c** Standardized climatic variable PERMANOVA results at *P. brincki + P. parvicorpus + P. tuerkayi* sampling localities | | |
| --- | --- | --- |
|  | populations | regions |
| Permutation N: | 9999 | 9999 |
| Total sum of squares: | 209 | 209 |
| Within-group sum of squares: | 152.9 | 187.7 |
| F: | 1.649 | 1.135 |
| p (same): | 0.1494 | 0.3601 |

| **TABLE** **S3.14e** Kruskal-Wallis test on the bioclimatic variables at *P. brincki* *+ P. parvicorpus + P. tuerkayi* sampling localities, with significant values indicated in bold | | | | | | |
| --- | --- | --- | --- | --- | --- | --- |
|  |  | H (chi2) | p (same) | A (N = 6) | B (N = 4) | C (N = 2) |
| Bio1 | Annual Mean Temperature | 1.1538 | 0.5616 | 16.05 | 15.7 | 16.48 |
| Bio2 | Mean Diurnal Range (Mean of monthly (max temp - min temp)) | 2.0513 | 0.3586 | 9.99 | 8.54 | 9.91 |
| Bio3 | Isothermality (BIO2/BIO7) (×100) | 3.109 | 0.2113 | 51.98 | 52.21 | 53.59 |
| Bio4 | Temperature Seasonality (standard deviation ×100) | 1.0513 | 0.5912 | 335.06 | 297.79 | 310.6 |
| Bio5 | Max Temperature of Warmest Month | 1.8461 | 0.3973 | 25.73 | 24.38 | 26.23 |
| Bio6 | Min Temperature of Coldest Month | 0 | 1 | 7.67 | 7.65 | 7.72 |
| Bio7 | Temperature Annual Range (BIO5-BIO6) | 1.4936 | 0.4739 | 19.28 | 16.76 | 18.51 |
| Bio8 | Mean Temperature of Wettest Quarter | 1.4167 | 0.4925 | 12.76 | 12.19 | 12.67 |
| Bio9 | Mean Temperature of Driest Quarter | 1.0513 | 0.5912 | 19.59 | 19.17 | 19.72 |
| Bio10 | Mean Temperature of Warmest Quarter | 1.4423 | 0.4862 | 19.63 | 18.17 | 20.21 |
| Bio11 | Mean Temperature of Coldest Quarter | 1.4167 | 0.4925 | 12.76 | 1.19 | 12.67 |
| Bio12 | Annual Precipitation | 2.6667 | 0.2636 | 643.5 | 749 | 574 |
| Bio13 | Precipitation of Wettest Month | 4.9038 | 0.08613 | 106.5 | 115 | 76.5 |
| Bio14 | Precipitation of Driest Month | 1.9956 | 0.3687 | 17 | 25.5 | 24 |
| **Bio15** | **Precipitation Seasonality (Coefficient of Variation)** | **7.8205** | **0.02004** | **64.36** | **55.21** | **41.46** |
| Bio16 | Precipitation of Wettest Quarter | 4.9211 | 0.08539 | 304 | 335.5 | 223.5 |
| Bio17 | Precipitation of Driest Quarter | 0.99117 | 0.6092 | 56 | 78.5 | 77 |
| Bio18 | Precipitation of Warmest Quarter | 1.2628 | 0.5318 | 56.5 | 78.5 | 78 |
| Bio19 | Precipitation of Coldest Quarter | 4.9211 | 0.08539 | 403 | 335.5 | 223.5 |

**TABLES S3.15** Climate variable statistics for the *Potamonautes perlatus* *+ P. barnardi + P. barbarai* sampling localities

| **TABLE** **S3.15a** Standardised climatic variable loadings at *P. perlatus + P. barnardi + P. barbarai* sampling localities for the first two principal components. Strongly-loading variables are indicated in bold | | |
| --- | --- | --- |
|  | PC 1 (44.79%) | PC2 (26.2%) |
| Bio1 | -0.12934 | 0.18027 |
| Bio2 | **-0.32662** | -0.0035098 |
| Bio3 | -0.12299 | 0.24886 |
| Bio4 | -0.29669 | -0.14381 |
| Bio5 | **-0.30582** | -0.016536 |
| Bio6 | 0.20188 | 0.075607 |
| Bio7 | **-0.32001** | -0.049923 |
| Bio8 | -0.079655 | **0.38302** |
| Bio9 | -0.025006 | -0.2504 |
| Bio10 | -0.23938 | 0.09106 |
| Bio11 | 0.049166 | 0.23672 |
| Bio12 | **0.32089** | -0.027766 |
| Bio13 | 0.28784 | -0.18761 |
| Bio14 | 0.23014 | 0.29795 |
| Bio15 | 0.012266 | **-0.40916** |
| Bio16 | 0.27658 | -0.21279 |
| Bio17 | 0.22962 | 0.29722 |
| Bio18 | 0.19732 | **0.32706** |

| **TABLE** **S3.15b** Spearman's correlation between the standardised bioclimatic variables at *P. perlatus* *+ P. barnardi + P. barbarai* sampling localities and longitude, with significant Spearman's rank coefficients (rs) and corresponding p-values indicated in bold | | |
| --- | --- | --- |
|  | rs | p |
| Bio1 | 0.1961144 | 0.2808 |
| Bio2 | -0.0813783 | 0.6568 |
| **Bio3** | **0.5124633** | **0.003068** |
| Bio4 | -0.2155425 | 0.2351 |
| Bio5 | -0.1759531 | 0.334 |
| Bio6 | -0.059023 | 0.7483 |
| Bio7 | -0.1722874 | 0.3443 |
| **Bio8** | **0.6620235** | **5.60E-05** |
| **Bio9** | **-0.5417889** | **0.001603** |
| Bio10 | 0.160022 | 0.3816 |
| Bio11 | 0.1634897 | 0.3698 |
| Bio12 | -0.0139322 | 0.9397 |
| Bio13 | -0.2193087 | 0.2278 |
| **Bio14** | **0.6416716** | **7.56E-05** |
| **Bio15** | **-0.938783** | **2.20E-16** |
| Bio16 | -0.2590047 | 0.1523 |
| **Bio17** | **0.6529116** | **5.11E-05** |
| **Bio18** | **0.709086** | **5.55E-06** |
| **Bio19** | **-0.4692942** | **0.006735** |

| **TABLE** **S3.15c** Standardized climatic variable PERMANOVA results at *P. perlatus + P. barnardi + P. barbarai* sampling localities | | |
| --- | --- | --- |
|  | populations | regions |
| Permutation N: | 9999 | 9999 |
| Total sum of squares: | 589 | 589 |
| Within-group sum of squares: | 448.2 | 509.7 |
| F: | 4.556 | 4.669 |
| p (same): | **0.0003** | **0.0028** |

| **TABLE** **S3.15d** Pairwise PERMANOVA post-hoc test results between *P. perlatus* *+ P. barnardi + P. barbarai* clades. F statistics are shown below the diagonal, with p-values above the diagonal | | | |
| --- | --- | --- | --- |
|  | A | B | C |
| A |  | 0.4155 | **0.0004** |
| B | 0.9035 |  | **0.0033** |
| C | 6.724 | 4.723 |  |

| **TABLE** **S3.15e** Kruskal-Wallis test on the bioclimatic variables at *P. perlatus* *+ P. barnardi + P. barbarai* sampling localities, with significant values indicated in bold | | | | | | |
| --- | --- | --- | --- | --- | --- | --- |
|  |  | H (chi2) | p (same) | A (N = 10) | B (N = 6) | C (N = 16) |
| Bio1 | Annual Mean Temperature | 0.71951 | 0.6978 | 16.18 | 17.06 | 17.19 |
| Bio2 | Mean Diurnal Range (Mean of monthly (max temp - min temp)) | 3.3871 | 0.1839 | 13.2 | 10.97 | 12.31 |
| **Bio3** | **Isothermality (BIO2/BIO7) (×100)** | **12.526** | **0.001905** | **52.14** | **51.94** | **54.02** |
| Bio4 | Temperature Seasonality (standard deviation ×100) | 0.8651 | 0.6489 | 416.56 | 381.92 | 367.94 |
| Bio5 | Max Temperature of Warmest Month | 1.2803 | 0.5272 | 29.27 | 27.36 | 27.51 |
| Bio6 | Min Temperature of Coldest Month | 2.0777 | 0.3539 | 6.36 | 6.46 | 5.67 |
| Bio7 | Temperature Annual Range (BIO5-BIO6) | 1.9932 | 0.3691 | 25.16 | 21.49 | 22.61 |
| **Bio8** | **Mean Temperature of Wettest Quarter** | **17.818** | **0.0001352** | **12.31** | **12.8** | **18.49** |
| **Bio9** | **Mean Temperature of Driest Quarter** | **12.551** | **0.001882** | **20.85** | **20.9** | **12.49** |
| Bio10 | Mean Temperature of Warmest Quarter | 0.87971 | 0.6441 | 21.1 | 20.91 | 21.5 |
| Bio11 | Mean Temperature of Coldest Quarter | 0.064347 | 0.9683 | 12.31 | 12.8 | 12.57 |
| Bio12 | Annual Precipitation | 2.887 | 0.2361 | 299 | 500.5 | 371 |
| Bio13 | Precipitation of Wettest Month | 5.8097 | 0.05476 | 51 | 67.5 | 44 |
| **Bio14** | **Precipitation of Driest Month** | **9.9619** | **0.006868** | **9.5** | **20.5** | **22** |
| **Bio15** | **Precipitation Seasonality (Coefficient of Variation)** | **23.647** | **7.33E-06** | **62.37** | **42.8** | **18.58** |
| **Bio16** | **Precipitation of Wettest Quarter** | **7.5387** | **0.02307** | **136.5** | **197.5** | **112** |
| **Bio17** | **Precipitation of Driest Quarter** | **10.27** | **0.005888** | **34** | **68.5** | **76.5** |
| **Bio18** | **Precipitation of Warmest Quarter** | **12.522** | **0.001909** | **37** | **68.5** | **94.5** |
| **Bio19** | **Precipitation of Coldest Quarter** | **15.443** | **0.0004431** | **136.5** | **197.5** | **101.5** |

**TABLES S3.16** Climate variable statistics for the *Peripatopsis capensis* *+ Per. lawrencei + P. overbergiensis* sampling localities

| **TABLE** **S3.16a** Standardised climatic variable loadings at *Per. capensis* *+ Per. lawrencei + P. overbergiensis* sampling localities for the first two principal components. Strongly-loading variables are indicated in bold | | |
| --- | --- | --- |
|  | PC 1 (40.61%) | PC2 (32.99%) |
| Bio1 | 0.0066119 | **0.37654** |
| Bio2 | **-0.32423** | -0.10326 |
| Bio3 | 0.054654 | 0.040587 |
| Bio4 | **-0.32189** | -0.092554 |
| Bio5 | -0.27121 | 0.16706 |
| Bio6 | 0.23778 | 0.2837 |
| Bio7 | **-0.32309** | -0.1047 |
| Bio8 | -0.017689 | **0.36125** |
| Bio9 | 0.10258 | 0.21045 |
| Bio10 | -0.15561 | **0.32418** |
| Bio11 | 0.1493 | **0.34242** |
| Bio12 | **0.30027** | -0.16781 |
| Bio13 | **0.31922** | -0.10554 |
| Bio14 | 0.036302 | -0.29934 |
| Bio15 | **0.31071** | 0.047838 |
| Bio16 | **0.31955** | -0.10502 |
| Bio17 | 0.018544 | -0.27721 |
| Bio18 | -0.041675 | **-0.30124** |
| Bio19 | **0.31984** | -0.10507 |

| **TABLE S3.16b** Spearman's correlation between the standardised bioclimatic variables at *Per. capensis* *+ Per. lawrencei + P. overbergiensis* sampling localities and longitude, with significant Spearman's rank coefficients (rs) and corresponding p-values indicated in bold | | |
| --- | --- | --- |
|  | rs | p |
| Bio1 | -0.0877193 | 0.7295 |
| **Bio2** | **0.8204334** | **2.86E-05** |
| Bio3 | 0.131063 | 0.6033 |
| **Bio4** | **0.7585139** | **0.0004016** |
| **Bio5** | **0.5108359** | **0.03214** |
| **Bio6** | **-0.5789474** | **0.01334** |
| **Bio7** | **0.7812178** | **0.000195** |
| Bio8 | -0.0092879 | 0.9738 |
| Bio9 | -0.131063 | 0.6033 |
| Bio10 | 0.2363261 | 0.3436 |
| Bio11 | -0.376677 | 0.1241 |
| **Bio12** | **-0.7131063** | **0.001265** |
| **Bio13** | **-0.8739674** | **2.14E-06** |
| Bio14 | 0.1102478 | 0.6632 |
| **Bio15** | **-0.9731682** | **8.067E-06** |
| **Bio16** | **-0.8800432** | **1.47E-06** |
| Bio17 | 0.2653578 | 0.2872 |
| Bio18 | 0.3226481 | 0.1916 |
| **Bio19** | **-0.8800432** | **1.47E-06** |

| **TABLE** **S3.16c** Standardized climatic variable PERMANOVA results at *Per. capensis* *+ Per. lawrencei + P. overbergiensis* sampling localities | | |
| --- | --- | --- |
|  | populations | regions |
| Permutation N: | 9999 | 9999 |
| Total sum of squares: | 323 | 323 |
| Within-group sum of squares: | 138.6 | 47.46 |
| F: | 4.51 | 3.09 |
| p (same): | **0.0002** | **0.0199** |

| **TABLE** **S3.16d** Pairwise PERMANOVA post-hoc test results between *Per. capensis* *+ Per. lawrencei + P. overbergiensis* clades. F statistics are shown below the diagonal, with p-values above the diagonal | | | |
| --- | --- | --- | --- |
|  | A | B | C |
| A |  | **0.0332** | 0.2451 |
| B | 4.013 |  | **0.0009** |
| C | 4.564 | 7.119 |  |

| **TABLE** **S3.16e** Kruskal-Wallis test on the bioclimatic variables at *Per. capensis* *+ Per. lawrencei + P. overbergiensis* sampling localities, with significant values indicated in bold | | | | | | |
| --- | --- | --- | --- | --- | --- | --- |
|  |  | H (chi2) | p (same) | A (N = 2) | B (N = 12) | C (N = 4) |
| Bio1 | Annual Mean Temperature | 2.4257 | 0.2963 | 16.23 | 15.14 | 16.03 |
| **Bio2** | **Mean Diurnal Range (Mean of monthly (max temp - min temp))** | **10.327** | **0.00572** | **8.21** | **10.10** | **11.93** |
| Bio3 | Isothermality (BIO2/BIO7) (×100) | 2.5994 | 0.2726 | 52.23 | 53.04 | 52.70 |
| **Bio4** | **Temperature Seasonality (standard deviation ×100)** | **9.9327** | **0.006968** | **277.31** | **325.76** | **398.91** |
| **Bio5** | **Max Temperature of Warmest Month** | **7.2895** | **0.02613** | **24.42** | **25.14** | **27.65** |
| Bio6 | Min Temperature of Coldest Month | 4.7135 | 0.09473 | 8.71 | 6.08 | 4.10 |
| **Bio7** | **Temperature Annual Range (BIO5-BIO6)** | **9.3889** | **0.009146** | **15.71** | **19.05** | **22.66** |
| Bio8 | Mean Temperature of Wettest Quarter | 4.2573 | 0.119 | 12.90 | 11.13 | 12.68 |
| Bio9 | Mean Temperature of Driest Quarter | 0.5117 | 0.7743 | 19.55 | 19.02 | 18.88 |
| Bio10 | Mean Temperature of Warmest Quarter | 5.4854 | 0.0644 | 19.59 | 19.05 | 20.78 |
| Bio11 | Mean Temperature of Coldest Quarter | 2.3889 | 0.3029 | 12.90 | 11.14 | 11.13 |
| **Bio12** | **Annual Precipitation** | **9.1696** | **0.01021** | **595.5** | **650.67** | **370.25** |
| **Bio13** | **Precipitation of Wettest Month** | **9.1886** | **0.01011** | **100** | **94.58** | **41.25** |
| Bio14 | Precipitation of Driest Month | 5.0519 | 0.07998 | 16.5 | 25.17 | 21.75 |
| **Bio15** | **Precipitation Seasonality (Coefficient of Variation)** | **10.889** | **0.00432** | **65.61** | **47.94** | **20.99** |
| **Bio16** | **Precipitation of Wettest Quarter** | **9.2076** | **0.01001** | **280** | **269** | **112.25** |
| Bio17 | Precipitation of Driest Quarter | 3.3179 | 0.1903 | 54 | 79.42 | 72.25 |
| Bio18 | Precipitation of Warmest Quarter | 3.9902 | 0.136 | 54.5 | 80.17 | 76.75 |
| **Bio19** | **Precipitation of Coldest Quarter** | **9.2076** | **0.01001** | **4.24** | **56.30** | **25.60** |

| TABLE S3.17 Bioclimatic variables displaying significant differences between poplations per taxon | | | | | | | | | | | | | | | | | | | |
| --- | --- | --- | --- | --- | --- | --- | --- | --- | --- | --- | --- | --- | --- | --- | --- | --- | --- | --- | --- |
| Taxon | Bio1 | Bio2 | Bio3 | Bio4 | Bio5 | Bio6 | Bio7 | Bio8 | Bio9 | Bio10 | Bio11 | Bio12 | Bio13 | Bio14 | Bio15 | Bio16 | Bio17 | Bio18 | Bio19 |
| *H. areolatus* |  |  | X | X |  |  | X | X | X |  |  |  | X | X | X | X | X | X | X |
| *C. angulata* |  |  | X |  |  |  |  | X |  |  | X | X | X | X | X | X | X | X | X |
| *D. lutrix* | X |  | X | X |  |  |  |  | X | X | X | X | X | X | X | X | X | X | X |
| *A. meleagris* |  | X | X | X | X | X | X | X | X |  | X | X | X | X | X | X | X | X | X |
| *P. brincki*  + *P. parvicorpus*  *+ P. tuerkayi* |  |  |  |  |  |  |  |  |  |  |  |  |  |  | X |  |  |  |  |
| *P. perlatus*  + *P. barbarai*  *+ P. barnardi* |  |  | X |  |  |  |  | X | X |  |  |  |  | X | X | X | X | X | X |
| *Per. capensis +*  *Per. lawrencei +*  *Per. overbergiensis* |  | X |  | X | X |  | X |  |  |  |  | X | X |  | X | X |  |  |  |

| TABLE S3.18 Spearman’s correlation rank coefficients (rs) between bioclimatic variables and longitude | | | | | | | | | | | | | | | | | | | |
| --- | --- | --- | --- | --- | --- | --- | --- | --- | --- | --- | --- | --- | --- | --- | --- | --- | --- | --- | --- |
| Taxon | Bio1 | Bio2 | Bio3 | Bio4 | Bio5 | Bio6 | Bio7 | Bio8 | Bio9 | Bio10 | Bio11 | Bio12 | Bio13 | Bio14 | Bio15 | Bio16 | Bio17 | Bio18 | Bio19 |
| *H. areolatus* | -0.10 | -0.24 | **0.42** | **-0.46** | **-0.45** | 0.21 | -0.31 | 0.40 | **-0.51** | -0.37 | -0.39 | -0.12 | **-0.43** | **0.67** | **-0.88** | **-0.48** | **0.64** | **0.75** | **-0.58** |
| *C. angulata* | -0.42 | -0.39 | **-0.61** | 0.2 | -0.31 | -0.18 | -0.004 | -0.14 | -0.36 | -0.26 | -0.40 | **0.65** | 0.43 | **0.84** | -0.68 | -0.41 | **0.86** | **0.85** | 0.31 |
| *D. lutrix* | **0.57** | 0.30 | **0.76** | -0.26 | 0.13 | 0.06 | -0.02 | **0.70** | **-0.43** | 0.29 | **0.61** | -0.09 | **-0.61** | **0.76** | **-0.91** | **-0.67** | **0.81** | **0.83** | **-0.81** |
| *A. meleagris* | 0.17 | 0.28 | **0.45** | -0.04 | -0.07 | **-0.31** | 0.18 | **0.65** | **-0.68** | 0.03 | 0.14 | **0.43** | 0.11 | **0.59** | **-0.67** | 0.08 | **0.62** | **0.97** | **-0.61** |
| *P. brincki*  + *P. parvicorpus*  *+ P. tuerkayi* | -0.22 | 0.43 | **0.64** | 0.22 | 0.23 | -0.56 | 0.36 | -0.48 | -0.07 | -0.11 | -0.48 | 0.34 | 0.06 | **0.69** | **-0.90** | 0.05 | **0.72** | **0.71** | 0.05 |
| *P. perlatus*  + *P. barbarai*  *+ P. barnardi* | 0.20 | -0.08 | **0.51** | -0.22 | -0.18 | -0.06 | -0.17 | **0.66** | **-0.54** | 0.16 | 0.16 | -0.01 | -0.22 | **0.64** | **-0.94** | -0.26 | **0.65** | **0.71** | **-0.47** |
| *Per. capensis +*  *Per. lawrencei +*  *Per. overbergiensis* | -0.09 | **0.82** | 0.13 | **0.76** | **0.51** | -0.58 | **0.78** | 0.01 | -0.13 | 0.24 | -0.38 | -0.71 | **-0.87** | 0.11 | **-0.97** | **-0.88** | 0.27 | 0.32 | **-0.88** |

| **TABLE S3.19** Redundancy analysis (RDA) results testing the relationship between genetic variance and sets of abiotic variables. All results are statistically significant (p<0.05) | | | | |
| --- | --- | --- | --- | --- |
| Taxon and locus | Model | Variance (adjusted r2) | Variance (r2) | p-value |
| *H. areolatus* | Full | 0.74914 | 0.831511 | 0.001 |
| ND4 | Climate | 0.2459029 | 0.3060317 | 0.001 |
|  | Distance | 0.0421064 | 0.03702568 | 0.004 |
|  | CFM | 0.1277189 | 0.09143178 | 0.001 |
|  | Distance + CFM | 0.1591073 | 0.1252199 | 0.001 |
|  | Distance + Climate | 0.2983963 | 0.3725704 | 0.001 |
|  | CFM + Climate | 0.5551508 | 0.6134627 | 0.001 |
| *C. angulata* | Full | 0.8550106 | 0.8842745 | 0.001 |
| ND4 | Climate | 0.2240163 | 0.2431241 | 0.001 |
|  | Distance | 0.01651477 | 0.01614489 | 0.011 |
|  | CFM | 0.039940096 | 0.03357609 | 0.001 |
|  | Distance + CFM | 0.05563744 | 0.04992971 | 0.001 |
|  | Distance + Climate | 0.4601369 | 0.4838492 | 0.001 |
|  | CFM + Climate | 0.3820498 | 0.4016433 | 0.001 |
| *D. lutrix* | Full | 0.7036707 | 0.7954911 | 0.001 |
| ND4 | Climate | 0.2532571 | 0.3218555 | 0.001 |
|  | Distance | 0.02472461 | 0.02610723 | 0.026 |
|  | CFM | 0.0283515 | 0.0241395 | 0.017 |
|  | Distance + CFM | 0.06772041 | 0.062119 | 0.005 |
|  | Distance + Climate | 0.344075 | 0.4268755 | 0.001 |
|  | CFM + Climate | 0.6417978 | 0.707192 | 0.001 |
| *A. meleagris* | Full | 0.7205446 | 0.7472752 | 0.001 |
| COI | Climate | 0.3350676 | 0.3537826 | 0.001 |
|  | Distance | 0.04586908 | 0.04431051 | 0.001 |
|  | CFM | 0.0549875 | 0.05111946 | 0.001 |
|  | Distance + CFM | 0.1047661 | 0.09975654 | 0.001 |
|  | Distance + Climate | 0.4036351 | 0.4273956 | 0.001 |
|  | CFM + Climate | 0.5464245 | 0.5659734 | 0.001 |
| *P. brincki + P. parvicorpus*  *+ P. tuerkayi* | Full | 0.9855828 | 0.9880818 | 0.001 |
|  | Climate | 0.09559003 | 0.09368872 | 0.001 |
| COI | Distance | 0.003989066 | 0.003543045 | 0.001 |
|  | CFM | no constrained component | | |
|  | Distance + CFM | 0.003543045 | 0.003989066 | 0.001 |
|  | Distance + Climate | 0.1462599 | 0.1466166 | 0.001 |
|  | CFM + Climate | 0.2494843 | 0.2449459 | 0.001 |
| *P. perlatus + P. barbarai*  *+ P. barnardi* | Full | 0.7215503 | 0.7709527 | 0.001 |
|  | Climate | 0.37328 | 0.4069147 | 0.001 |
| COI | Distance | 0.0547573 | 0.0504166 | 0.001 |
|  | CFM | 0.02707595 | 0.02473607 | 0.001 |
|  | Distance + CFM | 0.1048175 | 0.09549346 | 0.001 |
|  | Distance + Climate | 0.6174671 | 0.6596443 | 0.001 |
|  | CFM + Climate | 0.4812907 | 0.5184391 | 0.001 |
| *Per. capensis + Per. lawrencei*  *+ Per. overbergiensis* | Full | 0.8973944 | 0.9112958 | 0.001 |
|  | Climate | 0.1333809 | 0.1427148 | 0.001 |
| COI | Distance | 0.004067706 | 0.004893027 | 0.001 |
|  | CFM | 0.004071099 | 0.004207768 | 0.002 |
|  | Distance + CFM | 0.008419448 | 0.00942762 | 0.001 |
|  | Distance + Climate | 0.5539215 | 0.5635872 | 0.001 |
|  | CFM + Climate | 0.2695737 | 0.2786728 | 0.001 |

| **TABLE S3.20** Mean mutation rates for the concatenated datasets used in the Ecoevolity analyses, with the weighted mutation rate means for combinations of loci | | | | |
| --- | --- | --- | --- | --- |
|  |  | Length |  | Rate |
| Species and loci | | (bp) |  | (s/s/my) |
| ***H. areolatus*** | | | | |
|  |  |  |  |  |
| ND4 | ND4 | 680 |  | 0.0039616 |
|  | Total | 860 | W/mean | 0.0039616 |
| ND4 + PRLR | ND4 | 680 |  | 0.0040488 |
|  | PRLR | 514 |  | 0.00023208 |
|  | Total | 1194 | W/mean | 0.00240576 |
| ***C. angulata*** | | | | |
| ND4 + cytb | ND4 | 791 |  | 0.00337 |
|  | cytb | 320 |  | 0.0036738 |
|  | Total | 1111 | W/mean | 0.0034575 |
| ***D. lutrix*** | | | | |
| ND4 + cytb | ND4 | 740 |  | 0.0154 |
|  | cyb | 610 |  | 0.0134 |
|  | Total | 1350 | W/mean | 0.0144963 |
| ND4 + cytb + SPTBN1 | ND4 | 740 |  | 0.0133 |
|  | cytb | 610 |  | 0.0136 |
|  | SPTBN1 | 760 |  | 0.0053777 |
|  | Total | 2110 | W/mean | 0.0105332 |
| ***A. meleagris*** | | | | |
| COI | COI | 552 |  | 0.006055 |
|  |  |  | W/mean | 0.006055 |
| COI + EXPH5 | |  |  |  |
|  | COI | 552 |  | 0.006055 |
|  | EXPH5 | 642 |  | 0.001606 |
|  | Total | 1194 | W/mean | 0.00366282 |
| ***P. brincki + P. parvicorpus + P. tuerkayi*** | | | | |
| COI | COI | 614 |  | 0.0194 |
|  | Total | 614 | W/mean | 0.0194 |
| ***P. perlatus + P. barbarai + P. barnardi*** | | | | |
| 16S + COI | 16S | 455 |  | 0.003883 |
|  | COI | 655 |  | 0.03818 |
|  | Total | 1110 | W/mean | 0.02412132 |
| ***Per. capensis + Per. lawrencei + Per. overbergiensis*** | | | | |
| COI | COI | 638 |  | 0.019 |
|  | Total | 638 | W/mean | 0.019 |

**TABLE S3.21** Comparative phylogeographic results for the focal taxa using ecoevolity, showing the summarised posterior distributions for different numbers of divergence events using both flat and independent prior settings. Bayes factors show the support for specific numbers of events. The largest Bayes factor values for each prior are indicated in bold

a. Flat prior

| Number of Events | Posterior probability | Cumulative posterior probability | Prior probability | Bayes factor |
| --- | --- | --- | --- | --- |
| **3** | **0.453** | **0.453** | **0.343** | **1.587** |
| 4 | 0.360 | 0.814 | 0.399 | 0.849 |
| 5 | 0.105 | 0.919 | 0.160 | 0.614 |
| 2 | 0.073 | 0.992 | 0.072 | 1.022 |
| 6 | 0.008 | 1 | 0.024 | 0.336 |
| 1 | <0.001 | 1 | 0.001 | <1.075 |
| 7 | <0.001 | 1 | 0.001 | <1.002 |

b. Independent prior

| Number of Events | Posterior probability | Cumulative posterior probability | Prior probability | Bayes factor |
| --- | --- | --- | --- | --- |
| 7 | 0.335 | 0.335 | 0.501 | 0.501 |
| 6 | 0.324 | 0.659 | 0.316 | 1.038 |
| 5 | 0.217 | 0.876 | 0.124 | 1.965 |
| 4 | 0.09 | 0.966 | 0.041 | 2.298 |
| **3** | **0.03** | **0.996** | **0.013** | **2.302** |
| 2 | 0.004 | 1 | 0.004 | 1.007 |
| 1 | <0.001 | 1 | 0.001 | <1.079 |

**TABLE S3.22** PCF support values for each combination of species included in the PCF analysis of *P. perlatus + P. barbarai + P. barnardi* (P), *A. meleagris* (A), *H. areolatus* (H) and *D. lutrix* (D). Highly supported values (>0.71) are indicated in bold

| *K* | PCF average | Species composition |
| --- | --- | --- |
| 5 | 0.5735 | PACDH |
| 4 | 0.6065 | ACDH |
| 4 | 0.577 | PCDH |
| **4** | **0.716** | **PADH** |
| 4 | 0.557 | PACH |
| 4 | 0.6205 | PACD |
| 3 | 0.4795 | CDH |
| **3** | **0.8075** | **ADH** |
| 3 | 0.526 | ACH |
| 3 | 0.6155 | ACD |
| 3 | 0.6235 | PDH |
| 3 | 0.578 | PCA |
| 3 | 0.661 | PCD |
| 3 | 0.67 | PAH |
| **3** | **0.759** | **PAD** |
| 3 | 0.6355 | PAC |
| **2** | **0.716** | **DH** |
| 2 | 0.518 | CH |
| 2 | 0.5235 | CD |
| **2** | **0.7855** | **AH** |
| **2** | **0.9205** | **AD** |
| 2 | 0.498 | AC |
| 2 | 0.63 | PH |
| **2** | **0.756** | **PD** |
| **2** | **0.7415** | **PC** |
| **2** | **0.7165** | **PA** |
